# The Surface-Topography Challenge: A Multi-Laboratory Benchmark Study to Advance the Characterization of Topography

**DOI:** 10.1007/s11249-025-02014-y

**Published:** 2025-07-26

**Authors:** A. Pradhan, M. H. Müser, N. Miller, J. P. Abdelnabe, L. Afferrante, D. Albertini, D. A. Aldave, L. Algieri, N. Ali, A. Almqvist, T. Amann, P. Ares, B. N. Balzer, L. Baugh, E. A. Berberich, M. Björling, M. S. Bobji, F. Bottiglione, B. Brodmann, W. Cai, G. Carbone, R. W. Carpick, F. Cassin, J. Cayer-Barrioz, M. I. Chowdhury, M. Ciavarella, E. Cihan, D. Huang, E. Delplanque, A. J. Deptula, S. Descartes, A. Dhinojwala, M. Dienwiebel, D. Dini, A. C. Dunn, C. Edwards, M. Eriten, A. Esawi, R. M. Espinosa-Marzal, L. Fang, A. Fatemi, C. Fidd, D. Gabriel, F. Gaslain, G. Giordano, J. Gómez-Herrero, L. Gontard, N. N. Gosvami, G. Greenwood, C. Greiner, T. Grejtak, A. Haroun, M. Hasan, S. Hoppe, L. Isa, R. L. Jackson, S. Jang, O. Johnson, F. Kaiser, M. Kalin, K. Kalliorinne, P. H. Karanjkar, S. H. Kim, S. Kinzelberger, P. Klapetek, B. A. Krick, C. Kumar, N. Kumar, S. Kumar, P. LaMascus, R. Larsson, P. Laux, M. J. Lee, P. M. Lee, W. Lee, C. Leriche, J. Li, Y. Li, Y. -S. Li, T. A. Lubrecht, I. A. Lyashenko, C. Ma, T. Ma, F. Maaboudallah, S. Mahmood, F. Mangolini, M. Marian, D. Mazuyer, Y. Meng, N. Menga, T. Miller, D. M. Mulvihill, M. Najah, D. Nečas, C. I. Papadopoulos, A. Papangelo, M. Pauli, B. N. J. Persson, A. Peterson, A. A. Pitenis, P. Podsiadlo, M. Polajnar, V. L. Popov, T. Požar, A. Prasad, G. Prieto, C. Putignano, M. H. Rahman, S. B. Ramisetti, S. Raumel, I. J. Reyes, N. Rodriguez, M. Rodríguez Ripoll, H. Rojacz, P. Sainsot, A. Samodurova, D. Savio, M. Scaraggi, F. Schaefer, S. W. Scherrer, K. D. Schulze, K. E. Shaffer, M. A. Sidebottom, D. Skaltsas, J. Soni, C. Spies, G. W. Stachowiak, L. Steinhoff, N. C. Strandwitz, K. Sun, S. Tripathi, W. R. Tuckart, S. Ugar, M. Valtr, K. E. Van Meter, J. Vdovak, J. G. Vilhena, G. Violano, G. Vorlaufer, M. Walczak, B. Weber, T. Woloszynski, M. Wolski, A. Yadav, V. A. Yastrebov, M. Yongjian, L. Yuan, J. Yus, J. Zhang, X. Zhang, Q. Zheng, L. Pastewka, T. D. B. Jacobs

**Affiliations:** 1 Department of Mechanical Engineering and Materials Science, University of Pittsburgh, 3700 O’Hara St., Pittsburgh, PA 15261 USA; 2 Department of Materials Science and Engineering, Saarland University, Campus C6 3, 66123 Saarbrücken, Germany; 3 IFISUR, Universidad Nacional del Sur/CONICET, Av. Alem 1253, Bahía Blanca, Buenos Aires, CP 8000 Argentina; 4 Department of Mechanics, Mathematics and Management, Polytechnic University of Bari, Via Orabona 4, Bari, 70125 Italy; 5 CNRS, Université Claude Bernard Lyon 1, INSA Lyon, INL, UMR 5270, Bldg I. Joliot Curie, 1 rue Enrico Fermi, 69622 Villeurbanne, France; 6 Departamento de Fí­sica de la Materia Condensada and Condensed Matter Physics Center (IFIMAC), Universidad Autónoma de Madrid, Facultad de Ciencias, C/ Francisco Tomás y Valiente 7, 28049 Madrid, Spain; 7 Department of Engineering for Innovation, University of Salento, Centro Ecotekne Pal. O - S.P. 6, 73100 Monteroni-Lecce, Italy; 8 Department of Mechanical Science and Engineering, University of Illinois Urbana-Champaign, 1206 W Green St, Urbana, IL 61801 USA; 9 Division of Machine Elements, Department of Engineering Science and Mathematics, Luleå University of Technology, 97187 Luleå, Sweden; 10 Fraunhofer Institute for Mechanics of Materials IWM, Tribology, Wöhlerstr. 11, 79108 Freiburg, Germany; 11 Department of Chemistry and Pharmacy, Institute of Physical Chemistry, University of Freiburg, Albertstr. 21, 79104 Freiburg, Germany; 12 Cluster of Excellence livMatS, Freiburg Center for Interactive Materials and Bioinspired Technologies, University of Freiburg, Georges-Köhler-Allee 105, 79110 Freiburg, Germany; 13 Freiburg Materials Research Center (FMF), University of Freiburg, Stefan-Meier-Str. 21, 79104 Freiburg, Germany; 14 Department of Mechanical Engineering, Auburn University, 354 War Eagle Way, Wiggins Hall, AL Auburn, 36849 USA; 15 Department of Mechanical and Manufacturing Engineering, Miami University, 650 E High St, Oxford, OH 45056 USA; 16 Mechanical Engineering, Indian Institute of Science, C V Raman Ave, Bengaluru, 560012 India; 17 OptoSurf GmbH, Nobelstr. 9-13, 76275 Ettlingen, Germany; 18 School of Food Science and Engineering, Engineering Research Center of Bio-process (Ministry of Education), Hefei University of Technology, Tunxi Road. 193, 230009 Hefei, Anhui Province China; 19 Mechanical Engineering and Applied Mechanics, University of Pennsylvania, 220 S. 33rd St., Philadelphia, PA 19104-6315 USA; 20 LTDS, CNRS UMR5513, Ecole Centrale de Lyon, 36 Avenue Guy de Collongue, 69130 Ecully, France; 21 Department of Materials Science and Engineering, Lehigh University, 5 East Packer Avenue, Bethlehem, PA 18015 USA; 22 Institute for Materials Science and Max Bergmann Center for Biomaterials, TU Dresden, 01069 Dresden, Germany; 23 Center for Advancing Electronics Dresden (cfaed), TU Dresden, 01069 Dresden, Germany; 24 Saint-Gobain Omniseal Solutions, Heiveldekens 22, 2550 Kontich, Belgium; 25 LaMCoS, UMR 5259, INSA Lyon, CNRS, Bldg S. Germain, 27bis Ave. Jean Capelle, 69621 Villeurbanne, France; 26 School of Polymer Science and Polymer Engineering, University of Akron, 170 University Ave, Akron, OH 44325 USA; 27 Mikrotribologie Centrum µTC, Fraunhofer Institute for Mechanics of Materials IWM, Rintheimer Querallee 2b, 76131 Karlsruhe, Germany; 28 Institute for Applied Materials, Karlsruhe Institute of Technology (KIT), Kaiserstraße 12, 76131 Karlsruhe, Germany; 29 Department of Mechanical Engineering, Exhibition Road, Imperial College London, London, SW7 2AZ United Kingdom; 30 Department of Mechanical and Aerospace Engineering, University of Florida, 1064 Center Drive, Building NEB Room 181, Gainesville, FL 32611 USA; 31 Walker Department of Mechanical Engineering, The University of Texas at Austin, 204 E. Dean Keeton, Austin, TX 78712-1591 USA; 32 Department of Mechanical Engineering, University of Wisconsin-Madison, 1513 University Ave, Madison, WI 53706 USA; 33 Department of Mechanical Engineering, The American University in Cairo, AUC Avenue, P.O. Box 74, New Cairo, 11835 Egypt; 34 Department of Materials Science and Engineering, University of Illinois at Urbana-Champaign, 1304 W. Green St., Urbana, IL 61801 USA; 35 Department of Civil and Environmental Engineering, University of Illinois at Urbana-Champaign, 205 N Mathews Ave, Urbana, IL 61801 USA; 36 Robert Bosch GmbH, Robert-Bosch-Campus 1, Renningen, 71272 Germany; 37 FAMU-FSU College of Engineering, Mech. Eng. Dept, 2003 Levy Ave, Tallahassee, FL 32310 USA; 38 Currenta GmbH & Co. OHG, Surface and Solid-State Analytics, CHEMPARK Leverkusen, 51368 Leverkusen, Germany; 39 Centre des matériaux, MINES Paris - PSL, CNRS UMR 7633, 21 allée des Marronniers, 78000 Versailles, France; 40 BD Medical-Pharmaceutical Systems, 11 Rue Aristide-Verges, 38801 Le Pont de Claix, France; 41 Department of Materials Science and Engineering, Indian Institute of Technology Delhi, Hauz Khas, New Delhi Delhi, 110016 India; 42 Materials Science and Technology Division, Oak Ridge National Laboratory, 1 Bethel Valley Road, Oak Ridge, TN 37830 USA; 43 Department of Materials, ETH Zürich, Leopold-Ruzicka-Weg 4, 8093 Zürich, Switzerland; 44 Department of Chemical Engineering and Materials Research Institute, Pennsylvania State University, University Park, PA 16802 USA; 45 Tribology Department, Freudenberg Technology Innovation SE & Co.KG, Höhnerweg 2-4, 69469 Weinheim, Germany; 46 Laboratory for Tribology and Interface Nanotechnology (TINT), Faculty of Mechanical Engineering, University of Ljubljana, Aškerceva cesta 6, 1000 Ljubljana, Slovenia; 47 Czech Metrology Institute, Okružní­ 31, 63800 Brno, Czech Republic; 48 CEITEC, Brno University of Technology, Purkynova 123, 61200 Brno, Czech Republic; 49 Materials and Manufacturing Research Group, James Watt School of Engineering, University of Glasgow, Glasgow, G12 8QQ United Kingdom; 50 Science and Technology Division, Corning Incorporated, 184 Science Center Dr, Painted Post, NY 14870 USA; 51 Fraunhofer Institute for Physical Measurement Techniques IPM, Georges-Köhler-Allee 301, 79110 Freiburg, Germany; 52 Southwest Research Institute, 6220 Culebra Rd, San Antonio, TX 78238 USA; 53 Advanced Research Center for NanoLithography, University of Amsterdam, Science Park 106, 1098 XG Amsterdam, The Netherlands; 54 Department of System Dynamics and Friction Physics, Institute of Mechanics, Technische Universität Berlin, Straße des 17. Juni 135, 10623 Berlin, Germany; 55 Samarkand State University, University blv. 15, 140104 Samarkand, Uzbekistan; 56 Department of Precision Machinery and Precision Instrumentation, University of Science and Technology of China, Jinzhai Road 96, 230226 Hefei, Anhui Province China; 57 Chair of Materials Science and Nanotechnology, Technical University Dresden, BudapesMaterials Department, University of California, Santa Barbara, Santa Barbara, CA 93106, USA ter Str. 27, 01069 Dresden, Germany; 58 State Key Laboratory of Tribology in Advanced Equipment, Tsinghua University, No.1, Section 4, Chengfu Road, 100084 Beijing Haidian District, China; 59 Department of Mechanical Engineering, Université de Sherbrooke, 2500 Bd de l’Université, QC Sherbrooke, J1K 2R1 Canada; 60 Department of Mechanical Engineering, Auburn University, 311 W Magnolia Ave, Gavin Research Laboratory, Auburn, AL 36849 USA; 61 Department of Mechanical and Metallurgical Engineering, School of Engineering, Pontificia Universidad Católica de Chile, Vicuña Mackenna 4860, Macul, Santiago, 6904411 Chile; 62 Characterization Department, Freudenberg Technology Innovation SE & Co.KG, Höhnerweg 2-4, 69469 Weinheim, Germany; 63 School of Naval Architecture and Marine Engineering, National Technical University of Athens, 9 Heroon Polytechniou Street, 15710 Zografos, Greece; 64 Polytec GmbH, Polytecplatz 1-7, 76337 Waldbronn, Germany; 65 Peter Grünberg Institute (PGI-1), Forschungszentrum Jülich, 52425 Jülich, Germany; 66 Department of Plastics Engineering, University of Massachusetts Lowell, 1 University Ave., Ball 213, Lowell, MA 01854 USA; 67 Materials Department, University of California, Santa Barbara, Santa Barbara, CA 93106 USA; 68 Tribology Laboratory, School of Civil and Mechanical Engineering, Curtin University, GPO Box U1987, Perth, WA 6845 Australia; 69 Independent Researcher, Chennai, TN 600041 India; 70 Institute for Micro Production Technology, Leibniz University Hanover, An der Universität 2, 30823 Garbsen, Germany; 71 BD Medical-Pharmaceutical Systems, 1 Becton Drive, Franklin Lakes, NY 07417 USA; 72 AC2T research GmbH, Viktor-Kaplan-Strasse 2/C, 2700 Wiener Neustadt, Austria; 73 Center for Biomolecular Nanotechnologies, Istituto Italiano di Tecnologia, Via Barsanti 14, 73010 Arnesano, Italy; 74 Department of Material Science and Engineering, Saarland University, Campus D2 3 66123 Saarbrücken, Germany; 75 Department of Microsystems Engineering, University of Freiburg, Georges-Köhler-Allee 103, 79110 Freiburg, Germany; 76 Department of Plastics Engineering, University of Massachusetts Lowell, 40 University Ave., ETIC 215, Lowell, MA 01854 USA; 77 Materials Science Institute of Madrid ICMM-CSIC, C/ Sor Juana Inés de la Cruz 3, 28049 Madrid, Spain; 78 CSIR-Central Salt & Marine Chemicals Research Institute, Bhavnagar, Gujrat 364002 India; 79 Carl R. Woese Institute for Genomic Biology & Department of Mechanical Science and Engineering, University of Illinois at Urbana Champaign, 1206 W Gregory Dr, Urbana, IL 61801 USA

**Keywords:** Surface topography, Roughness metrics, Multi-scale topography, Challenge, Open Science

## Abstract

Surface performance is critically influenced by topography in virtually all real-world applications. The current standard practice is to describe topography using one of a few industry-standard parameters. The most commonly reported number is $R$a, the average absolute deviation of the height from the mean line (at some, not necessarily known or specified, lateral length scale). However, other parameters, particularly those that are scale-dependent, influence surface and interfacial properties; for example the local surface slope is critical for visual appearance, friction, and wear. The present Surface-Topography Challenge was launched to raise awareness for the need of a multi-scale description, but also to assess the reliability of different metrology techniques. In the resulting international collaborative effort, 153 scientists and engineers from 64 research groups and companies across 20 countries characterized statistically equivalent samples from two different surfaces: a “rough” and a “smooth” surface. The results of the 2088 measurements constitute the most comprehensive surface description ever compiled. We find wide disagreement across measurements and techniques when the lateral scale of the measurement is ignored. Consensus is established through scale-dependent parameters while removing data that violates an established resolution criterion and deviates from the majority measurements at each length scale. Our findings suggest best practices for characterizing and specifying topography. The public release of the accumulated data and presented analyses enables global reuse for further scientific investigation and benchmarking.

**Supplementary Information:**

The online version contains supplementary material available at 10.1007/s11249-025-02014-y.

## Introduction: The Purpose of this Challenge

Surface topography critically influences surface performance. Pioneering studies demonstrated its significance for contact conductance [1] and adhesion [2]. Over time, it has become evident that fatigue, fracture, fretting, friction, leakage, lubrication, the sound when sliding, tactile feel, visual appearance, and wear—even biocompatibility and mouthfeel—depend on surface topography as well. We now know that relevant topography features span many length scales [3–5], with the highest-bandwidth measurements today covering up to nine decades in length [6–9]. Therefore, the question of “How should surface topography be measured and characterized?” remains of daily relevance to product designers, manufacturers, and researchers, yet it lacks a universally accepted answer.

At present, the most common approach for specifying surface topography is to use a roughness parameter, as computed by a surface-analysis software package, according to international standards [10, 11]. Most commonly, this is $R$a – the average absolute deviation from the mean line – but other parameters are also used, such as the root-mean-square deviation *R*q and the peak-to-valley height difference *R*z. These parameters are typically obtained from a single topography measurement, most commonly using a stylus or optical profilometer in manufacturing, or often an atomic force microscope in research settings. In some cases, these roughness metrics will correlate with a property of interest, but frequently there is no simple relationship. This is because surfaces contain features across a wide array of length scales [3–9], and different properties are sensitive to different scales. Examples of crude guidelines are that leakage is most affected by large-scale topography, adhesion by intermediate-scale roughness, and the contact area of soft, elastic materials by small-scale surface slopes [12]. This highlights the need to understand and characterize surface topography as a scale-dependent property, which cannot be captured by a single number. Yet, researchers and manufacturers often lack an effective, standardized way to measure, report, and compare their topography data.

Among modelers and theoreticians, the understanding of topography-dependent properties has developed tremendously over the last 50 years, with roughly three categories of models for mechanical properties: independent-asperity models, like those by Greenwood-Williamson [13] and Bush-Gibson-Thomas [14]; the multi-scale contact theory developed by Persson [12, 15, 16]; and brute-force numerical approaches that solve the contact problem without making assumptions about how to statistically represent the rough topography [17–21]. In 2015, to help sort through the wide array of computational approaches and validate analytic theories, Martin Müser launched the Contact Mechanics Challenge [22], in which he publicly released a computer-generated virtual surface, and then invited the scientific community to compute its mechanical properties in a way that enabled comparison across disparate strategies. Although this prior Challenge was successful in its aims, it started from the assumption of complete knowledge of the topography of a surface, which is typically unavailable for real-world surfaces.

From the preceding discussion, it is clear that high-fidelity topography data are critical for predicting functional properties of rough interfaces. The Surface-Topography Challenge was launched to help address the lack of agreement on how to measure, report, and compare topography. Here, two distinct surfaces were chosen, and then hundreds of nominally identical samples were created for each surface. These samples were shipped free of charge to any group that volunteered to measure them. The goals and objectives were published in the original problem statement [23] and are quoted, with only minor wording modifications, as follows:

**The overall goal of the present Challenge** is for our community to move ourselves toward better understanding and agreement on how to measure, report, and analyze surface topography. This goal will be achieved through three objectives:

**Objective 1: Compare the advantages and disadvantages of different techniques for measuring surface topography.** The measurement of a single material using a wide variety of techniques and metrics enables the comparison and contrasting of results. This in turn elucidates the strengths and limitations of each technique.

**Objective 2: Generate the single most comprehensive description of a surface yet created.** By combining all results into a single statistical description of the material’s surface topography, we attempt to overcome the individual problems that are inherent to any single technique, such as instrument artifacts, noise, and limitations in scanning size or resolution. This fully comprehensive surface description provides a benchmark surface and a publicly available real-world dataset that can be used as an input to analytic or numerical calculations.

**Objective 3: Aid the development of next-generation surface descriptors.** Most of the commonly used statistical descriptions of surface topography use simplifying assumptions. For example, the distributions of surface height or surface slope are often approximated as Gaussian. By collecting and publishing the raw topography data for an extremely well-characterized surface, this Challenge enables the evaluation of the accuracy of these assumptions and may facilitate the generation of wholly new descriptors that more accurately describe topography.

## Methods for this Challenge: Creating Samples and Collecting Results

**Fig. 1:**
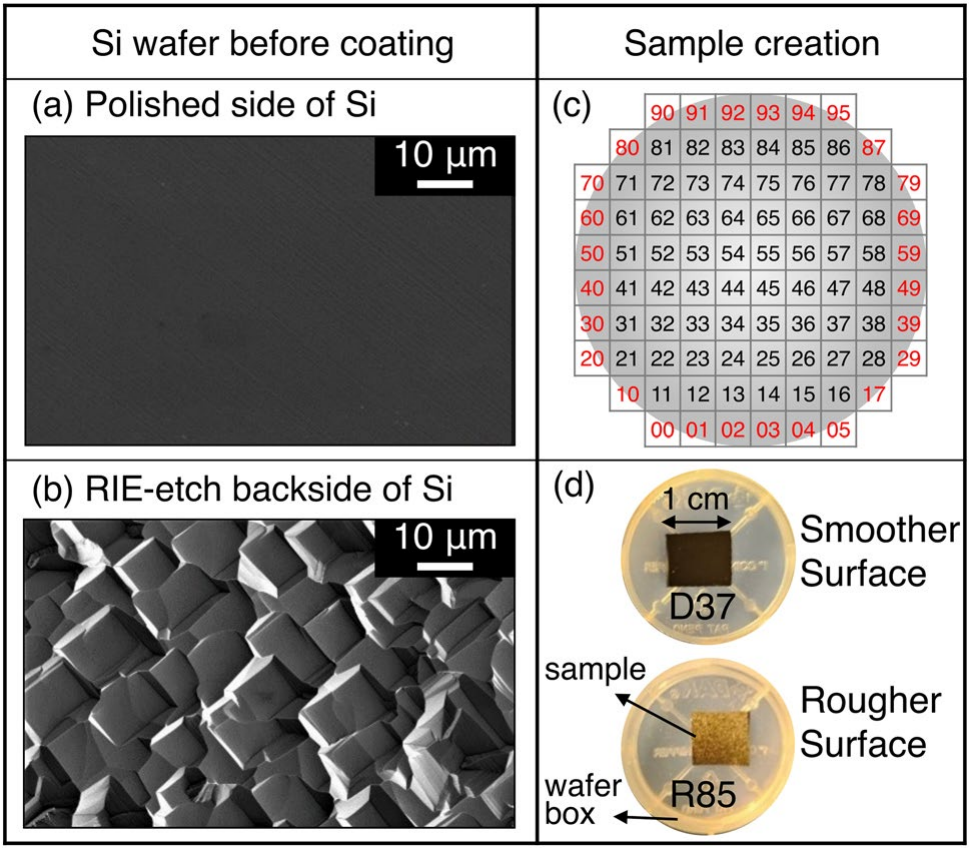
Hundreds of statistically identical samples were created for two surfaces. SEM image of **a** a polished silicon wafer, and **b** the reactive-ion-etched backside of another wafer. Both the “Smoother Surface” and the “Rougher Surface” were coated with CrN, a technologically relevant wear-resistant coating. For consistency, coatings were deposited in a single batch onto a group of 10-cm silicon wafers. **c** These wafers were sectioned into 1-cm samples, and given a unique index for tracking. Incomplete samples with missing edges, as indicated using red labels in Panel c, were excluded from distribution. Finally, **d** samples of each surface were packed in plastic wafer boxes and mailed out to participating groups

To create a large number of nominally identical samples, we leveraged techniques that are common in semiconductor manufacturing. Like with microchips, the samples comprised silicon wafers that were processed in a single large-scale batch and then sectioned for distribution to many different recipients. As the material for study, we chose chromium nitride, a wear- and corrosion-resistant coating material that is used in the automotive industry, and is used to coat cutting tools, molds, and dies across a wide range of metals manufacturing [24–26]. Specifically, the CrN coating was deposited using plasma-assisted magnetron sputtering. (Details of sample creation are described in Appendix A.) To minimize variation, all samples were fabricated in the same chamber, in a single processing step. Two different surfaces were created (Fig. 1):

- **The “Smoother Surface”:** a coating of CrN was deposited on prime-grade polished silicon wafers.
- **The “Rougher Surface”:** the same coating of CrN was deposited on the unpolished “backside” of other single-side-polished silicon wafers, which had been subjected to isotropic reactive-ion etching before deposition.

Once samples were created, we announced this Challenge in a publication [23] and at various surface-focused conferences. The sign-up opened in July of 2022, with an original deadline for measurement submission of August of 2023 (later extended to March 15, 2024). Two samples of each surface were mailed to each participating group, and groups were asked to measure the samples using any and all techniques that they would commonly use in the course of their research or manufacturing. Participants were asked to upload all raw data taken on a particular sample as a single *Digital Surface Twin* on the freely available, open-source topography-analysis platform contact.engineering [27]. This software platform homogenizes analysis workflows and ensures that all data were analyzed identically, removing user- and software-specific variations. Participants were also asked to submit a concise description of the methods and results; in cases where a report was submitted and consent was granted, these reports are included in the data deposition (see Data Availability). Later, they were asked to submit a supplementary-information form with relevant information about their group. These self-reported information forms are reproduced *verbatim* in the Supplementary Material of this article.

## Challenge Submissions: Measurements of the Same Materials by 153 People

**Fig. 2:**
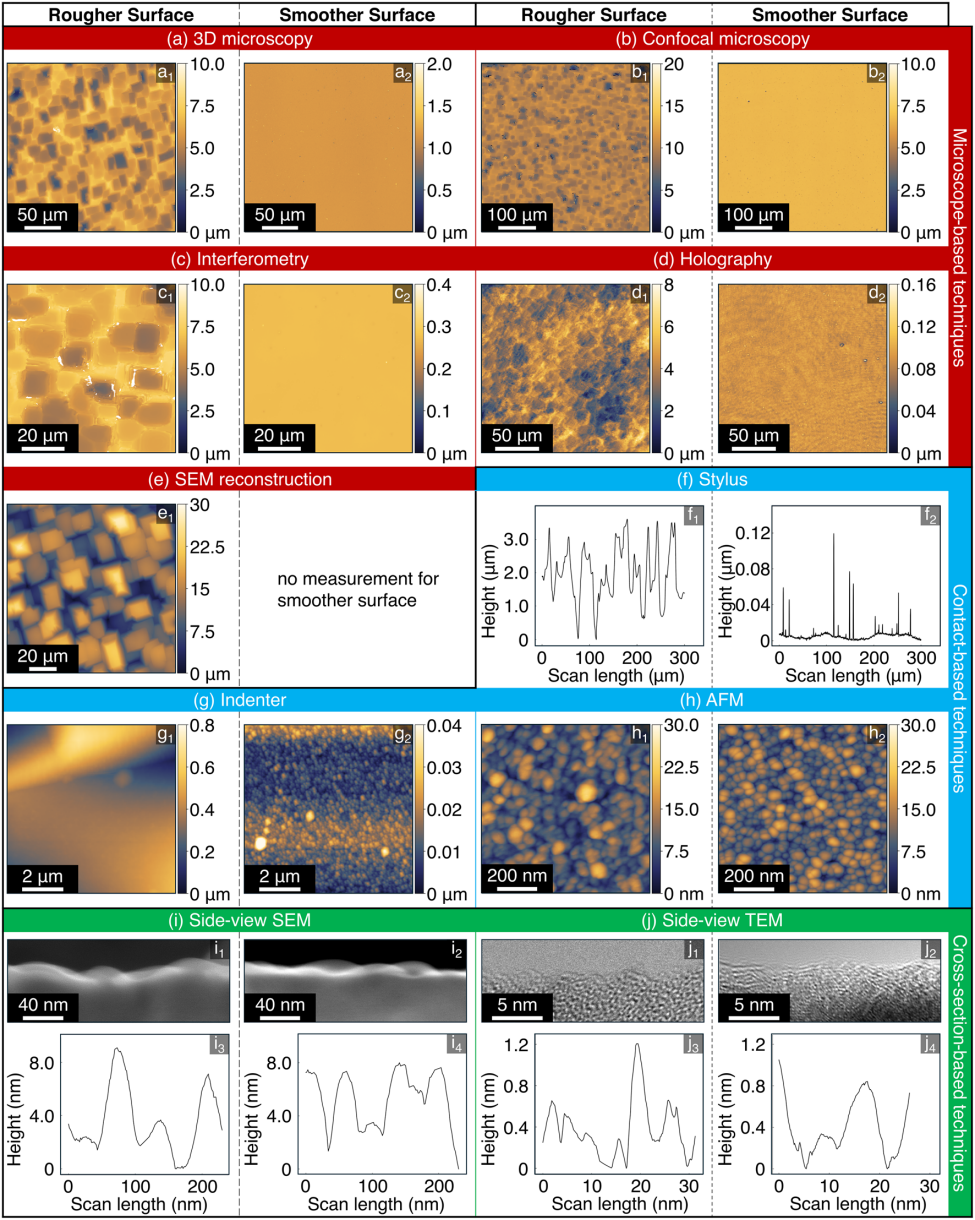
Illustrative examples are shown from each of the submitted techniques. Each pair of measurements shows the Rougher Surface (left) and the Smoother Surface (right). For visualization purposes, the height scale was limited to avoid an image being “washed out” by a tall peak; however, all raw data are available as described in the Data Availability section. The techniques are grouped according to the categories defined in the main text. The “short names” are used in order to correspond with legends, while the full name of each technique can be found in Section 3

The submissions to this Challenge consist of 2088 individual measurements, from 153 people, representing 64 different companies and research groups around the world. Figure 2 shows illustrative examples of different techniques applied to these samples. Individual measurements are categorized into three groups: microscope-based techniques; contact-based techniques; and cross-section-based techniques. Microscope-based techniques are any that use visible light (or electrons or x-rays or other) to analyze a surface from the top down in a non-contact fashion. Contact-based techniques are any that make (or get near to) physical contact with the sample in order to measure it. Finally, cross-section techniques analyze a surface using a profile view (or “side-view”) of the surface.

For those unfamiliar with any of the metrology techniques employed for the present study, the following sections contain a brief description of each. For clarity and specificity, each section lists the specific instrument models that participants reported for each technique. Of course a wide variety of manufacturers and models exist and we intend no endorsement of any kind. A brief name is given to each technique, for use throughout the manuscript and in legends. For easy reference, the start of each paragraph is formatted as: “Brief name”: Full name.

### Microscope-based Techniques

Microscopes use the interaction of light or electrons with an interface to generate an image of it. While a traditional optical microscope provides no topographic information in its image, there are a variety of working principles that allow the extraction of heights.

#### “3D microscopy”: Digital 3D Optical Microscopy, or Focus-Variation Microscopy

Digital 3D microscopes use standard microscope optics and compute the height at every pixel, most commonly using focus variation or fringe projection. In focus-variation microscopy, a narrow depth of field is swept vertically across the object, and a three-dimensional height map is constructed by recording the vertical position at which each pixel came into focus [28]. In fringe-projection profilometry, a pattern of lines is projected onto the sample, often with varying pitch and varying angle. Then, by analyzing the deformation of the projected lines on interaction with the sample, the surface topography can be reconstructed. The tools for 3D microscropy that were used in this investigation are the Bruker Alicona and the Keyence VR, VK, VKX, and VHX. The scan lengths for 3D microscopy range from approximately 100 $\upmu$m to several centimeters. The maximum lateral resolution will vary with lens configuration: it may be limited to tens of micrometers at low resolution, while at the highest possible magnification, it will be limited to a few micrometers. The vertical resolution is commonly on the order of 10 nm.

#### “Confocal microscopy”: Confocal Scanning Microscopy, or Laser Scanning Confocal Microscopy

This technique is similar in concept to focus-variation microscopy, but uses lasers and/or highly specialized optics to significantly reduce the depth of field, thus improving the vertical resolution. Most commonly, a single point is illuminated, and this point is raster-scanned in the two lateral dimensions while sweeping over the vertical dimension. These techniques are widely used and reviewed, see for example Ref. [29] for best practices for this technique. There were many different tools used for confocal microscopy in this Challenge: Keyence VK and VKX; CSM ConScan; Zeiss Smartproof; Olympus LEXT OLS; LEICA LSM and DCM; Sensofar Neox; Confovis TOOLinspect; RTEC UP-Lamda; NanoFocus Microsurf. Scan lengths and lateral resolution are similar to 3D microscopy (described above); the vertical resolution is on the order of 1 nm.

#### “Interferometry”: Scanning white-light Interferometry, or Phase-shifting Interferometry

Here, a beam of light is split into a reference beam, which goes straight to the detector, and a sample beam, which reflects off the sample and then goes to the detector [30]. The sample and reference beams will interfere with each other constructively or destructively, thus indicating whether the additional pathlength of the sample beam was an integer multiple of the wavelength of light. A single color (frequency) of light can be used to measure relative differences in height or, in white-light interferometry, a variety of colors create a coherence envelope that uniquely specifies a single height. To characterize a larger height variation, the sample is commonly scanned vertically [31]. For optical interferometry, the tools used were: Bruker Contour and NPFLEX; Filmetrics Profilm3D; RTEC MFT; Sensofar Neox; Polytec TopMap; Veeco Wyko NT; Zygo NewView, NexView, and ZeGage. Scan lengths and lateral resolution will be similar to 3D microscopy (above); the vertical resolution depends on the mode, and can range from 1 Å to tens of nanometers.

#### “Holography”: Digital Holographic Microscopy

A multi-wavelength digital holographic sensor reconstructs the surface height using phase information of light waves [32]. As in classic interferometric sensors, light is separated into a reference and object beam. Here, temporal phase-shifting is used to shift the phase of the reference or object wave. The system captures three interferograms per wavelength, reconstructing the complex object wave after extraction of the actual phase steps and thus compensating phase-shift deviations. Multi-wavelength digital holography combines the phase information of different wavelengths to achieve a large measurement range while maintaining precision [33]. The two reported tools for digital holographic microscopy were the Fraunhofer HoloTop [34] and the Lyncee Tec DHM. Lateral resolution will be similar to 3D microscopy, while vertical resolution can be on the order of single-digit nanometers.

#### “SEM reconstruction”: Scanning-electron-microscopy Reconstruction, or Stereo-SEM

From two (or more) SEM images of the same surface at different angles, the topography can be stereoscopically reconstructed [35, 36], similar to the depth perception of the human eyes. There are multiple ways of doing this, including taking multiple sequential images at a variety of surface-inclination angles, or taking simultaneous images using multiple detectors that have different orientations with respect to the sample [37]. Either way, a variety of numerical techniques can be applied to extract a three-dimensional topography from the multiple two-dimensional images. Common examples include standalone software packages that perform the calculations from SEM images, or 3D reconstruction packages offered by SEM manufacturers; the specific instrument used in this investigation was a ThermoFisher FEI Nova, coupled with a custom multi-detector analysis routine. The scan length of SEM can range from 100 nm to 100 $\upmu$m. The lateral resolution depends on the configuration of the SEM, but can be as small as single-digit nanometers. The vertical resolution is hard to determine, and may depend on the angle separating the original images.

### Contact-based Techniques

Contact-based instruments measure the vertical deflection of a sharp needle that is scanned across the surface as a function of its horizontal position. The scanning procedure of contact-based techniques produces line scans, but 3D topographic maps can be reconstructed from individual line scans if their relative position is known. However, perpendicular to the scan direction there are often artifacts associated with nonideal alignment of line scans. This group is called “contact-based”—even though some subsets are considered “non-contact” (such as scanning tunneling microscopy)—because all take place in a near-to-contact regime where there is a physical interaction between a tip and the surface.

#### “Stylus”: Stylus Profilometry, or Tactile Microscopy

Stylus profilometry employs sharp, needle-like tips with radii in the range of 1-10 $\upmu$m [38, 39]. Stylus profilometer instruments used in this investigation were the Bruker Dektak; Taylor Hobson TalySurf; Mitutoyo Surftest SJ; and Jenoptik Hommel-Etamic Waveline. The scan lengths can range from 100 $\upmu$m up to centimeters. The lateral resolution is limited by the end-radius of the scanning tip, which is typically on the order of single-digit micrometers. The vertical resolution is of order 1 nm.

#### “Indenter”: Scanning Nanoindenter

While nanoindenters are chiefly designed for measurements of mechanical properties such as hardness or modulus, they can be raster scanned to create a topographic image of the indentation [40]. This same mode can be used to generate topographic maps of any surface [41]. The scanning nanoindenter used in this investigation was the Bruker Hysitron TriboIndenter. The scan length, lateral, and vertical resolution are similar to stylus profilometry (described above); however, the end-radius of indenter tips are commonly larger.

#### “AFM”: Atomic Force Microscopy, or Scanning Probe Microscopy

AFMs use extremely sharp tips (in the range of 10-100 nm) combined with precise and rapid feedback about the tip position [42]. A wide variety of different modes of AFM exist, including contact mode (which is analogous to stylus profilometry), tapping mode (where the tip oscillates and makes only intermittent contact), and non-contact mode (where the tip senses surface forces without making physical contact) [43]. Many different AFM tools were used for this investigation: Oxford Instruments Jupiter, WITech Alpha, NanoWizard, and Asylum MFP3D and Cypher; Bruker (formerly Digital Instruments and Veeco) Dimension Icon, MultiMode, and Innova; Park Systems XE or NX; NT-MDT NTEGRA; SIOS Nanopositioning and Nanomeasuring Machine; NanoSurf Core and Drive; NanoTec Cervantes; and CSI Instruments NanoObserver. Scan lengths range from approximately 100 nm up to 10-100 $\upmu$m. Specialty designs, such as the Nanopositioning and Nanomeasurement Machine [44], can have much larger scan ranges, up to the cm scale. The lateral resolution can be limited by scan parameters, such as scan speed and accuracy of the feedback system; at best it is limited by tip-radius artifacts, and is therefore of order 10 nm for surfaces that are not atomically smooth. The vertical resolution is of order 1 Å.

### Cross-section-based Techniques

Instead of using a profilometer or microscope to measure the surface in a top-down configuration, it is possible to cross-section the surface and take a side-view image. By digitizing the contour of interest in the image, using edge-finding or manual-point-selection software routines, a quantitative line profile is extracted.

#### “Side-view SEM”: Scanning Electron Microscopy in Profile

This technique uses a standard SEM, plus post-processing image-analysis software, such as ImageJ or Matlab, to detect the boundary of the material, which constitutes the topography of the surface [45]. The three SEM tools used in this investigation were the ThermoFisher FEI Nova, the JEOL JIB, and the Zeiss Sigma. Scan lengths depend on SEM configuration, but typically range from 100 nm to 100 $\upmu$m. The lateral resolution is commonly affected by charging effects at the sharp corner of the cross-section, and therefore it is more commonly limited to approximately 10 nm. Unlike many of the techniques described above, the vertical resolution is identical to the lateral resolution; thus the maximum vertical resolution is 10 nm, but the resolution will be far coarser for large scan-size images. A more comprehensive description of the technique can be found in Ref. [46].

#### “Side-view TEM”: Transmission Electron Microscopy in Profile

This technique is similar to side-view SEM, but requires more sample preparation due to the special requirements of TEM [47]: The sample must be electron transparent ($<100$ nm in thickness) in the region of interest, and the entire sample must fit between the electromagnetic lenses, a gap on the order of 1 mm. A more thorough description can be found in Ref. [48]. The only TEM used in this investigation was a JEOL 2100F. Typical scan lengths range from 10 nm to 10 $\upmu$m. The lateral resolution depends on the lens configuration, but can be as low as single-digit Angstroms for high-resolution images. Once again, the vertical resolution is identical to the lateral resolution.

### Scattering Techniques

Scattering techniques characterize topography by analyzing the scattering of light or other types of waves off the surface. Height fluctuations on a rough surface will cause second-order scattering that will modify the reflected beam [49]. By varying the angle of the beam with respect to the surface normal, information can be gathered about a range of lateral wavelengths of topography variation.

#### “XRR”: X-ray Scattering

In XRR, X-rays are specularly reflected off of the surface, because of the low-wavelength typically with a grazing incidence angle. The intensity of the reflected beam is normalized by incidence intensity and plotted as a function of scattering angle. This technique is commonly used on multilayer films, simultaneously gathering information about each layer’s thickness, roughness, and density [50]. Specifically, the decay of intensity with angle can be modeled to extract the topography. The only XRR measurements submitted to this Challenge used a Malvern Panalytical Empyrean. Scan lengths of XRR are limited by the beam size and can be in the range of micrometers to millimeters. Lateral resolution is limited in theory by the diffraction limit of the light used (on the Angstrom-scale), but in practice is further limited by the approximations of the models used.

#### “ARS”: Angle-Resolved Optical Scattering

In ARS, light is reflected off of a surface, typically with perpendicular incidence. The sensor also captures the intensity of the scattered light as a function of angle. All ARS measurements in this Challenge were carried out with the Optosurf OS 500 sensor [51] and a wavelength of incident light of 670 nm. Scan lengths of ARS are limited by the beam size and can be up to centimeters. The limit of lateral resolution is the diffraction limit of light, around 1 $\upmu$m.

More generally, we note that the classes of techniques in the preceding three sub-sections, scattering techniques do not yield a topographic map. Instead the reflected light contains useful information characterizing the overall statistics of the topography. However, as described in Ref. [49], it is not straightforward to convert the measured data into standard statistical representations, such as RMS height or the power spectral density. For these reasons, scattering techniques are not included in the bulk of the analysis of the present paper, but are presented and discussed separately in Appendix C.

### File Formats

Participants were requested to submit data in the rawest form possible, not using any post-processing or file conversion. This resulted in submission in a variety of file formats, many of them without open, publicly available documentation. The platform contact.engineering [27] implements support for many of them, relying on the implicit documentation available in the open-source code of the widely used tool Gwyddion [52] for understanding some of these specific formats. Around 1/3 of submissions were received in generic text formats, as MATLAB files or as Gwyddion [52] data files. There is at least one format that has been standardized (X3P, whose metadata are described in ISO 5436-2 and ISO 25178), but it is not widely used. An overview of all data formats from the present submissions are listed in Appendix B. The organizers of this Challenge believe that the inherent inaccessibility of topography data are holding back more rapid progress in the field of surface metrology. To promote accessibility and interoperability of file formats in the spirit of the *Open Science* movement [53], we encourage manufacturers to more widely adopt open (non-binary), well-documented formats. Additionally, we encourage scientists and engineers to require such open file formats when purchasing instruments, and to report out data in these standard formats.

## Data Analysis and Discussion

The data were analyzed by the four main organizers of this Challenge (A. Pradhan, M. H. Müser, L. Pastewka and T. D. B. Jacobs). In this analysis section, the word “we” refers to these organizers. Because there are many ways to analyze topography data, it is important to be specific about data-analysis procedures (see Appendices D and E). Furthermore, by reporting the raw data, it can be subsequently reanalyzed with different sets of tools.

### Preparing the Data: Removing Tilt and Curvature

Our data analysis starts from the raw topographic data provided to us by participants. In most cases, this means we are reading the instrument-native, binary data format (see Appendix B). Exceptions are made for custom instrumentations and a few cases where documentation or reverse-engineering of the binary format was not possible. We assume that the data provided to us is not preprocessed, but we cannot exclude that some instruments apply filters before writing topography information to a file. All submitted data provided is tilt- and curvature-corrected before further analysis to account for tilt in the mounted samples and curvature artifacts that can be added by piezoelectric actuators and lens aberrations. Specifically, we subtracted a curve of the form $t(x)=z_0 + \alpha x + \beta x^2$ for line scans and a respective quadratic function *t*(*x*, *y*) for area scans. More details are given in Appendix D.

### Single-scale Parameters: Evaluating the Root-mean-square Height

**Fig. 3:**
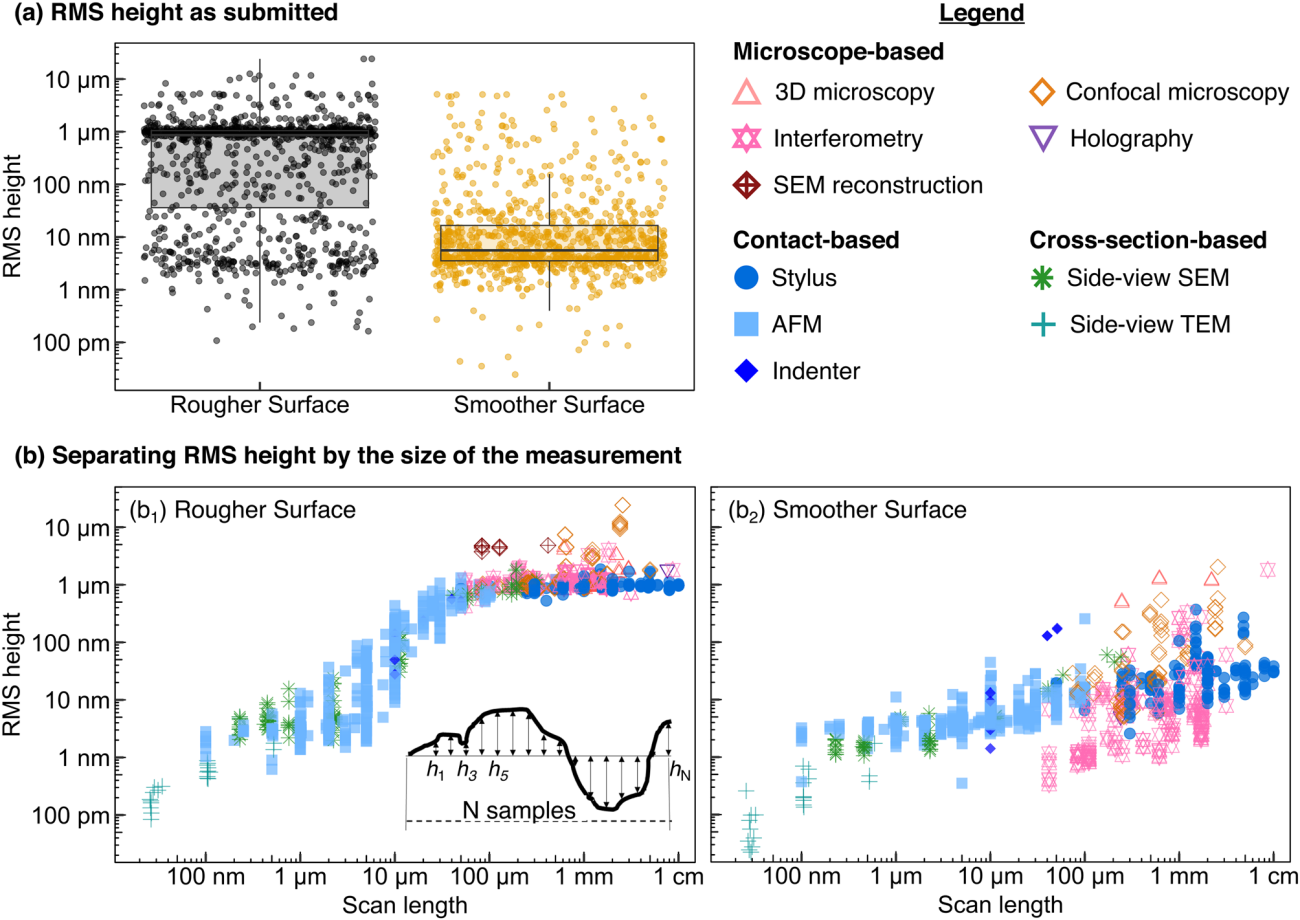
As-submitted, the values of the measured topography parameters for each surface spanned six orders of magnitude; much but not all of which was attributable to scale-dependent variation. **a** The root-mean-square height was calculated for each submission as submitted, on both the Rougher Surface and the Smoother Surface. **b** In presenting the data as a function of scan length, one significant source of this variability is revealed. **b**_**1**_ Shown here are the values for the Rougher Surface. **b**_**2**_ A similar plot of the Smoother Surface shows even greater variation at some key length scales. Throughout all figures, data points from microscope-based techniques will be presented using colors in the red family and hollow symbols, contact-based data appears in blues with solid symbols, and cross-section-based data appears in greens with lined symbols

To reduce each measurement to a single number, we compute the root-mean-square (RMS) height $h_\text {rms}$ (see Appendix D for details). We chose RMS height, instead of mean absolute deviation (*R*a) or other parameters, because the RMS height has useful advantages when describing a distribution, including maintaining the additive property of the variance. We note that, for area maps, the RMS height can differ when calculated for the entire area as compared to a line-by-line calculation. Because many metrology techniques produce only line scans, we carry out *all* analysis on line scans. For area scans, we compute the statistical parameter(s) of interest sequentially for each individual line scan, and then report their average (see Appendix D). For AFM measurements, the direction of analysis is the scanning direction, also called the “fast-scan” direction. Scattering techniques, which produced neither area scans nor line scans were excluded from the present analysis, and are discussed in Appendix C. Note that all the source-code to compute all parameters is available open-source, see Ref. [27].

The raw RMS height values are shown in Fig. 3a, where the box plot shows quartiles. There is a clear difference between mean values for the Rougher Surface and the Smoother Surface, yet this difference is overwhelmed by the deviations within a single surface. In their as-submitted state, the raw data for both surfaces yield RMS height variations from below 100 pm to 10 $\upmu$m, i.e., spanning six orders of magnitude! To understand the extent of the variation, we plot the RMS height as a function of the size of the measurement (Fig. 3$b_1$,$b_2$). The plots categorize the data by technique, indicated by symbol and color.

The first source of deviation becomes immediately clear: the magnitude of the topography variation depends on the size of the measurement. A 1-mm measurement captures larger height variations than a 1-$\upmu$m scan. This finding will not be surprising to the topography expert, but is worth explicitly pointing out to the casual user of roughness parameters. This reflects the fact, well-known in the topography community [3, 4], that topography is multi-scale, and should be thought of as bumps on top of bumps on top of bumps. Any statistical value extracted from a single measurement has little relevance in isolation: The statement “This sample has a roughness of 100 nm” is meaningless without specifying the length scale over which it was measured or calculated, and may still be insufficient if topography on multiple length scales is contributing to the property of interest.

### Establishing Consensus: Including only the Middle 50% of Measurements from Each Technique

Significant differences in measured results persist, even when the scale-dependence is taken into account. For example, measurements of the Rougher Surface with scan lengths of 10 $\upmu$m show a 100-fold difference in RMS height; when the smoother sample is measured with a lateral size of 1 mm, the RMS height varies by a factor of 1000.

The significant variability in $h_\text {rms}$ requires an objective way to reflect the “consensus” view among the participants. We sorted the data by technique, and then decided on a purely statistical basis, with no inspection nor subjective decision on individual measurements, to remove the top- and bottom-25% of all measurements to find the representative data for a given technique. This method removes all extreme values and, unlike the moments of a distribution function, it is insensitive to the specific values of these extremes; it falls into the class of *robust* statistics [54]. By eliminating all measurements that lie in the top 25% or bottom 25% of all $h_\text {rms}$ values for a specific technique, this leaves only measurements within the interquartile range (IQR) in the dataset.

**Fig. 4:**
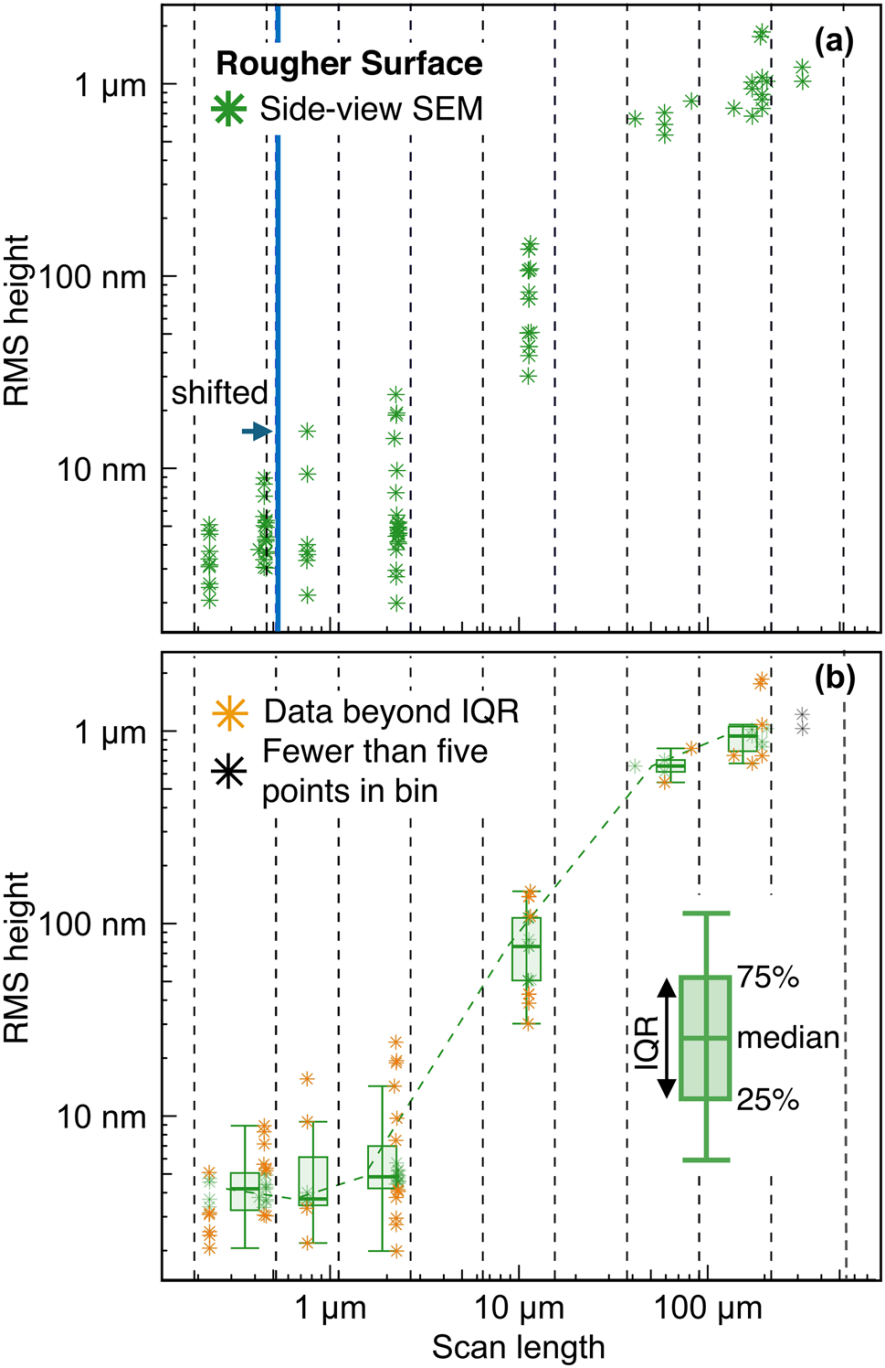
To establish consensus within each technique, the measurements are grouped by scan length and data beyond the interquartile range is removed. Panel **a** shows how the various measurements are binned by scan length, with SEM data for the Rougher Surface shown as an illustrative example. Log-spaced bins were created for each technique, as indicated by black dashed lines; in cases where a bin boundary coincides with one or more data points, then the bin boundary was shifted slightly rightward, as shown by the blue line. Panel **b** shows the same data, but now including box plots with median and IQR (i.e., all data lying between the 25th and 75th percentile). Points beyond the IQR are considered outermost values and denoted in yellow. For some bins, the number of measurements were less than five data points; in these cases, the median and IQR were not calculated (black data points)

We carried out this elimination of outermost measurements as a function of scale, by grouping all measurements from a specific technique using log-spaced bins. As an illustrative example, Fig. 4a shows the RMS height over scan length for one technique (side-view SEM) applied to one surface (the Rougher Surface). The smallest and largest scans determine the x-axis limits, then this region was divided into equispaced log bins (bounded by the dashed black lines). Inevitably, a few data points lie close to a bin boundary (within a relative tolerance $<0.01$); in these cases the bin boundary was shifted slightly to the right, as indicated by the blue line. Fig. 4b shows box plots for each bin with measurements outside the interquartile range indicated by yellow symbols. For some measurements, there are simply not enough data points within a bin to categorize data as within the IQR; this limit was chosen as five data points, and all data in length scales containing fewer data points are indicated by black color. Overall, this method allows us to find representative data within each technique and to remove variations within a given scan length without applying a subjective criterion.

**Fig. 5:**
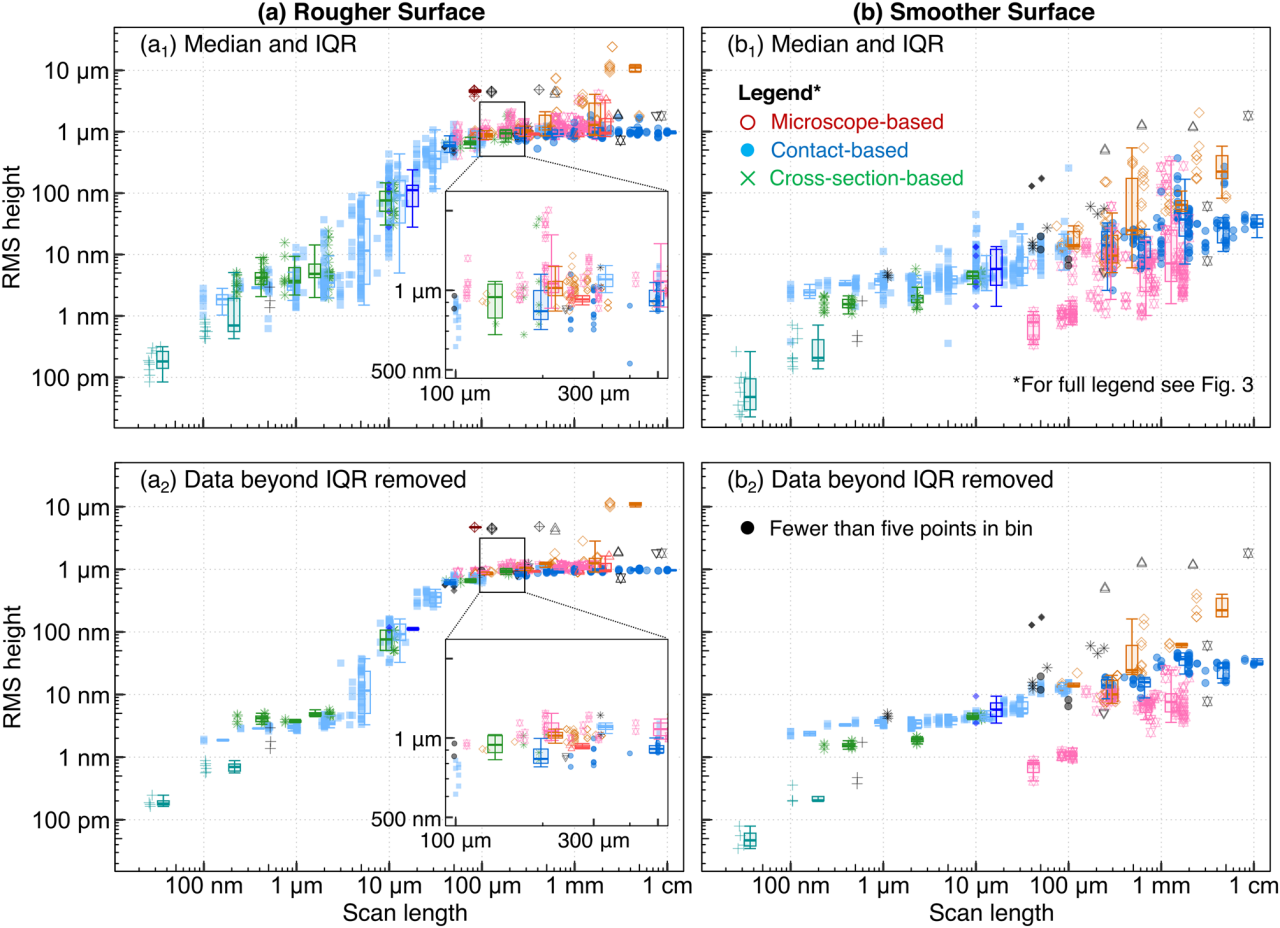
Large variation in data above and below the interquartile range is removed to find the representative measurements for each technique. **a** The RMS height measurement for the Rougher Surface is shown for the data as submitted. Plot **a**_**1**_ includes box plots signifying median and interquartile range, calculated by binning as shown in Fig. 4. The inset in **a**_**1**_ shows a zoomed-in view of RMS height from 100 $\upmu$m to 500 $\upmu$m of lateral scan length. Plot **a**_**2**_ shows the Rougher Surface after values beyond the IQR are removed. The variation in RMS height is now more closely aligned with the uncertainty that is inherent to each technique. **b** RMS height for the Smoother Surface shown before **b**_**1**_ and after **b**_**2**_ removing data beyond the IQR. For certain measurement techniques, there were insufficient data points to calculate the IQR; these are indicated by black symbols. All data points correspond to the legend shown in Fig. 3

Figure 5 shows the results of this analysis for all techniques. Panels $a_1$ (Rougher Surface) and $b_1$ (Smoother Surface) show all data points, highlighting median and IQR through solid lines and box plots. Figure 5$a_2$ and $b_2$ show the median and IQR of the remaining data after removal of the measurements with $h_\text {rms}$ in the top/bottom 25% of all points for a specific technique.

### Scale-dependent Parameters: The Power Spectral Density Reveals Contributions from Different Length Scales

**Fig. 6:**
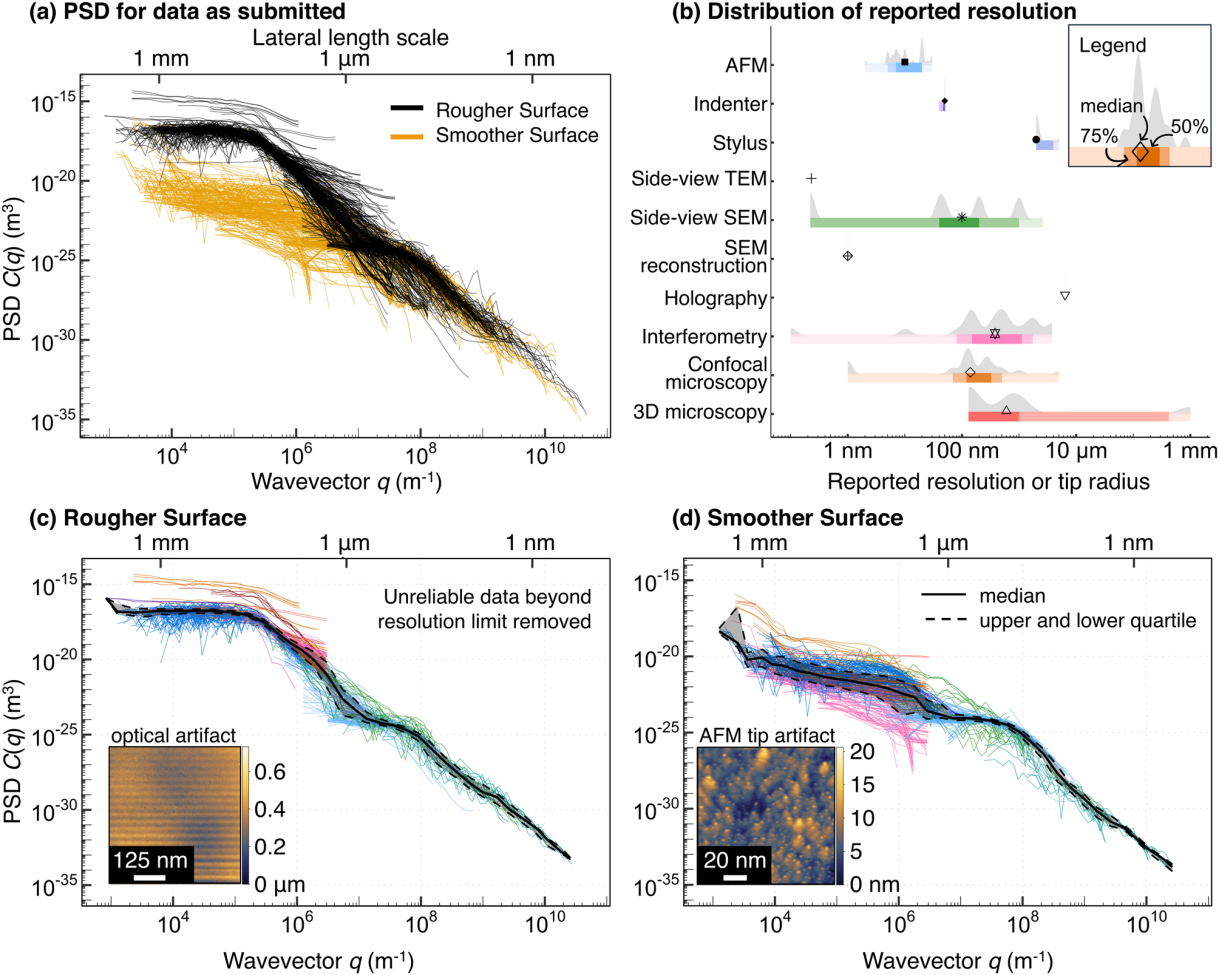
The power spectral density is used to eliminate unreliable data from within a single measurement. **a** The power spectral density (PSD) for the Rougher and Smoother Surfaces has large variation, even for the “consensus” data (based on IQR analysis, see main text). One reason for this is that plot (a) includes unreliable data beyond the resolution limits of the measurements. **b** Distribution of resolution or tip-size values reported by participants. For groups that did not report, we assigned values of 2 $\upmu$m, 2 $\upmu$m, and 20 nm for optical-microscope resolution, stylus tip size, and AFM tip size, respectively. For side-view SEM and TEM measurements, the resolution was estimated and a maximum-size cutoff was imposed. We then removed unreliable data from the PSD (see Appendix E) to produce **c**, **d** resolution-corrected PSDs for the Rougher and Smoother Surfaces. The solid black line is the median PSD from all the measurements combined, and the dashed line represents the IQR. The insets in (c,d) show examples of visible artifacts

While the RMS height is useful, it produces only a single number for each measurement, and does not reveal the multi-scale content that exists in each single measurement. The power spectral density (PSD) yields a roughness amplitude *C*(*q*) as a function of wavevector *q*. This spectral analysis separates out contributions from different length scales, as each wavevector corresponds to a different wavelength $\lambda =2\pi /q$. We here use the common convention of plotting the PSD in terms of wavevectors *q* (with shortest wavelength on the right-hand side of the plots), but note that some authors plot the PSD in terms of the wavelength $\lambda$ rather than *q* [55–57]. To accommodate both perspectives, the wavelength can be found on the top x-axis. The PSD for all data are shown in Fig. 6a. The PSD shown here was computed using the best practices described in Ref. [58], more details are given in Appendix E. The PSDs show that, overall, the two surfaces differ in roughness at large scales but have similar roughness at small scales. However, there was significant variation across submissions in the measured values at any given length scale; at some scales they vary by five orders of magnitude or more.

The first step in establishing consensus in the PSD was to remove all measurements that lie outside of the IQR in RMS height, as defined in the previous sub-section. The second step was to identify artifacts and eliminate data that may be unreliable because of artifacts. This process is described in the following sub-section.

### Eliminating Unreliable Data from Each Measurement: Accounting for Tip-based Artifacts and Resolution Limits

It is widely understood that the accuracy of topographic measurements is limited by the resolution of the technique. Broadly, all microscope-based techniques will be limited either by the diffraction limit of the imaging medium (visible light, electrons, etc.), or by the quality of the lenses [59, 60]. Likewise, all contact-based techniques will be limited by the radius of the portion of the tip that interacts with the sample [61, 62]. Any contributions to topography from scales below these resolution limits must be artifacted and therefore unreliable.

To account for these limitations, we removed unreliable data as follows: For microscope-based techniques, we excluded all data above the critical wavevector corresponding to the smallest discernible wavelength (sometimes termed the “resolution” of the measurement). For contact-based techniques, we used a tip-artifact-detection routine described in Ref. [63] (and inspired by the seminal works of Refs. [61, 62]), which is implemented in the open-source software platform contact.engineering [27].

In order to apply these corrections, we asked each submitting group to estimate the maximum lateral resolution of their instrument or the tip size of the probe. The distribution of reported values is shown in Fig. 6b. In cases where participants estimated the tip radius for contact-based techniques, we eliminated the unreliable data using this estimate as described in Ref. [63]. However, some participants did not report the tip size, due to the difficulty of measuring it. In such instances, we assumed that their tip size was equal to the average tip size from reporting groups (20 nm for AFM and 2 $\upmu$m for stylus profilometer and indenter measurements).

There was a larger spread in the reported resolution for microscope-based techniques as compared to contact-based techniques. For example, participants using interferometry reported lateral resolutions ranging from 100 pm to 5 $\upmu$m. However, it is known that optical microscopy techniques cannot measure accurately at lateral resolutions below the diffraction limit of visible light. Therefore, to be above this limit, we imposed a uniform cutoff value of 2 $\upmu$m for all optical techniques.

Finally, a maximum-size cutoff was imposed for side-view SEM and side-view TEM. As mentioned in the prior section, the vertical and lateral resolution of surfaces using these techniques are identical, therefore when a low magnification is used to capture a large scan length, then the “vertical” resolution (as defined relative to the original surface) is significantly diminished. For this reason, a maximum scan length of 500 $\upmu$m was imposed for cross-section-based techniques.

The resolution-corrected PSDs are shown in Fig. 6c,d for the Rougher and Smoother Surface. After resolution correction, the measurements are combined into one PSD by computing the median. The IQR range is shown by the shaded ribbon. Instead of using the average value, the median is chosen because it is a statistical parameter that is not as sensitive to the outermost measurements. When using the mean value, a single outlier datapoint varying by two orders of magnitude above the others will overwhelm the other values and unfairly draw the mean upward; however, when using the median, the same outlier will have no such effect. Therefore, we consider the median and IQR of the combination of PSDs more representative of the present surfaces as compared to the mean and standard deviation.

### Establishing a Single Descriptor for Each Surface: Removing Disagreements Between Techniques by a “Majority-rule” Approach

Finally, we attempted to extract a single description of topography that describes each surface as accurately and as completely as possible. Even after the data-correction procedures in the prior sub-sections, cases remain where two techniques simply disagree about the topography - as is evident in the real-space image, the RMS height, and the power spectral density. In order to arrive at a single description, a method was required to “break the tie” and remove one set of data. While we acknowledge that experts would be able to identify unreliable measurements from domain knowledge about a specific technique, the organizers of this Challenge did not wish to be in the position of determining whether some measurements were “good” and others were “bad”. So instead, we simply applied a majority-rule approach. Therefore, when a certain scale was identified where a majority of techniques yielded values with a certain PSD and a minority of techniques yielded values differing by more than a factor of two, then we simply removed the minority techniques. To avoid subjectivity, we chose not to remove only the length-scales where disagreement occurred, rather we removed the entire technique from consideration of that particular surface. These removals, along with some discussion of cause, are described in the following paragraphs.

For the Rougher Surface, it is apparent that the SEM reconstruction technique captures the details of the surface as seen in Fig. 2 but measures an RMS height and PSD that are higher than all other techniques at the same scale. In this case, the disparity was traced back to an incorrect calibration of height for the technique while combining images, which was discovered by the submitting group because of comparison to other submissions in the present Challenge. The submitting group corrected their data, but only after the Challenge was closed, so the data included in this manuscript is the original version. Similarly, holography is another technique that had a very small sample size of 4 measurements for the Rougher Surface. The present data deviated from other measurements, but there were simply not enough measurements to determine how successful it is at capturing roughness. The other technique showing a significant difference from consensus is the confocal microscopy, with significant deviations especially at large scan lengths (5X, 10X magnification). The sides of the plateau-like features were not accurately captured due to their high slopes, resulting in a significant fraction of missing data. While many instruments explicitly report which part of the data could not be acquired and is “missing”, other instruments automatically interpolate values between the plateaus. While many confocal measurements, especially those at smaller scan lengths agreed with consensus, the entire technique was removed from consideration to avoid subjectivity of choices about exclusion or inclusion.

For the Smoother Surface, a similar problem was observed for confocal microscopy at the largest scan lengths, and thus it was also removed. The Smoother Surface could not be measured by the groups that used holography and SEM reconstruction techniques. In fact, all microscope-based techniques including digital microscopy and interferometry deviated from the consensus, based on contact-based and cross-section-based techniques. This deviation is mainly attributed to the mismatch between the lateral and vertical resolution of optical methods: While optical techniques are capable of detecting height variations as small as 1 Å on surfaces with steps and wide plateaus, these techniques are not well suited to detecting height variations that occur within the lateral size of a single pixel (of order 1 $\upmu$m). While many groups were able to accurately measure topography with optical techniques, a sufficient number contained non-trivial deviations that these entire techniques were removed in order to eliminate subjectivity.

It is important to note that these choices about which techniques to include or exclude in the final determination are based solely on the submissions we received. In some cases, there was only a single submission representing an entire technique. Furthermore, it was common that some of the submissions on a given surface were in agreement with the majority opinion, while others were not. For all of these reasons, this process should not be misconstrued as making a judgement about the quality of a certain technique overall. Instead, the purpose of this sub-section is purely to arrive at a single, consensus topography for our two surfaces. The final outcome of this procedure is shown and discussed in the following section.

## Lessons Learned

The collection, analysis, and harmonization of this large number of measurements has demonstrated some of the challenges associated with topography measurement. Three lessons emerged from this two-year international collaboration. Each of the three lessons can be associated with a Topography Best Practice.

### Lesson 1: Using Two or more Surface-measurement Techniques Reveals Inconsistencies Across Techniques

**Fig. 7:**
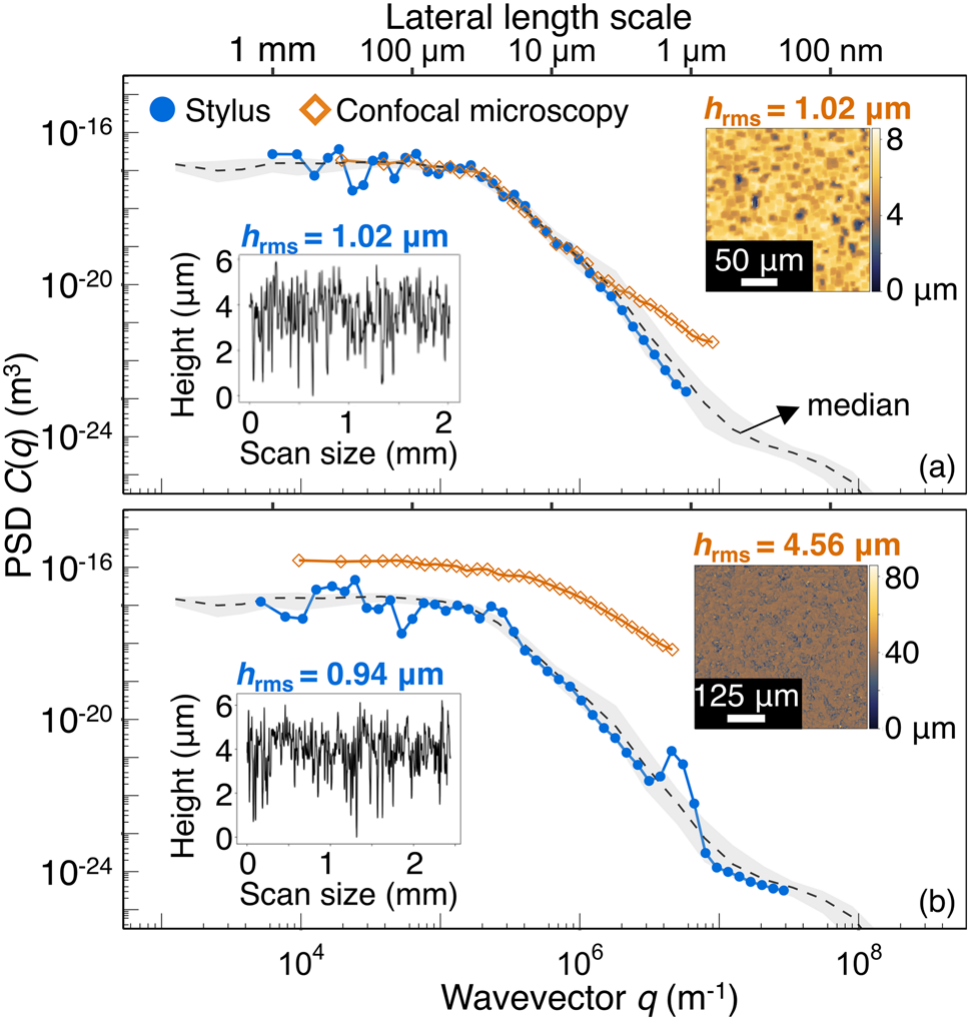
Lesson 1. Using two or more surface-measurement techniques reveals inconsistencies across techniques. **a** One group used both confocal microscopy and stylus to measure the Rougher Surface; both techniques produced similar values for the RMS height (despite different scan lengths) and similar PSDs. This similarity can be taken as evidence that the measured results are likely to be accurate. **b** A different group repeated the same protocol; using confocal microscopy and stylus to measure the Rougher Surface. However, this second group measured two vastly different results with the two different techniques. The inconsistency of their results can be taken as evidence that at least one of their measurements is inaccurate. The insets in **a**,**b** display the measurement data for the PSD corresponding to (left) stylus profilometry and (right) confocal microscopy. For reference, the “consensus” data from all groups is shown for the Rougher Surface; the black dashed line represents the median PSD and the gray ribbon indicates the interquartile range

It is not always easy to recognize artifacts or problems with our measurements. In this Challenge, we created a comprehensive statistical description of the surfaces by pooling many measurements from diverse sources. This allows us to use the IQR and majority-rule approach to determine the most accurate measurement of the topography. Of course when a user is characterizing a typical surface, there is a far smaller number of measurements and it is unclear whether the results would have fallen into the middle 50%. Therefore, to generalize from this experience, we propose a strategy to minimize the risk of error: measure a surface using multiple types of techniques.

For example, Fig. 7 compares two power spectral densities measured by two different submitting groups, all performed on the Rougher Surface. In one case (Fig. 7a), stylus profilometry and confocal microscopy produce near-identical results. In isolation, the person who measured this could not know that these measurements match the consensus view; however, the agreement between two disparate techniques would enable a high degree of confidence in the results. By contrast, in Fig. 7b, the same process was repeated by a different group: stylus and confocal microscopy were applied to the Rougher Surface. For this second group, the two techniques had virtually no agreement between either their PSDs nor their root-mean-square heights. While individual PSDs can vary due to local topography, this cannot explain the order-of-magnitude difference observed here. In isolation, the person who measured this could not have known which measurement would have agreed with the “consensus” measurement (shown in gray), but they would have understood that at least one of the measurements was likely to be unreliable.

The particular causes of artifacts in each technique are beyond the scope of this paper, but are discussed elsewhere (see, for example, Refs. [43, 59, 64]). Furthermore, while an expert user would sometimes be able to identify a particular measurement as artifacted and eliminate it from consideration, it is common to see artifacted measurements published in otherwise high-quality scientific articles. Therefore, it is clear that “expert knowledge” is not always adequate to produce reliable topography measurement. Instead, the agreement between two or more techniques produces high confidence, because there is a low probability that two unrelated measurements both fall outside the IQR, by a similar amount, and in the same direction.

#### Topography Best Practice 1: Combine Multiple Types of Measurements of the Same Surface

At a minimum, repeat measurements multiple times in multiple locations and with multiple orientations on the same sample, to understand the amount of fluctuation in results. Better yet, vary the scan length and pixel size (and possibly other parameters) of the measurement device. Better still is to apply several different measurement techniques to the same surface. All of these many measurements can be combined, without any adjustable fitting parameters, using multi-scale metrics. As shown in Fig. 7, the results immediately reveal any regions of disparity, and also enable the calculation of the mean or median result along with the amount of variation that is observed.

### Lesson 2: A Consistent Statistical Description Emerges when Lateral Length Scales are Accounted for, and Each Technique is Appropriately Corrected for Artifacts and Resolution limits

**Fig. 8:**
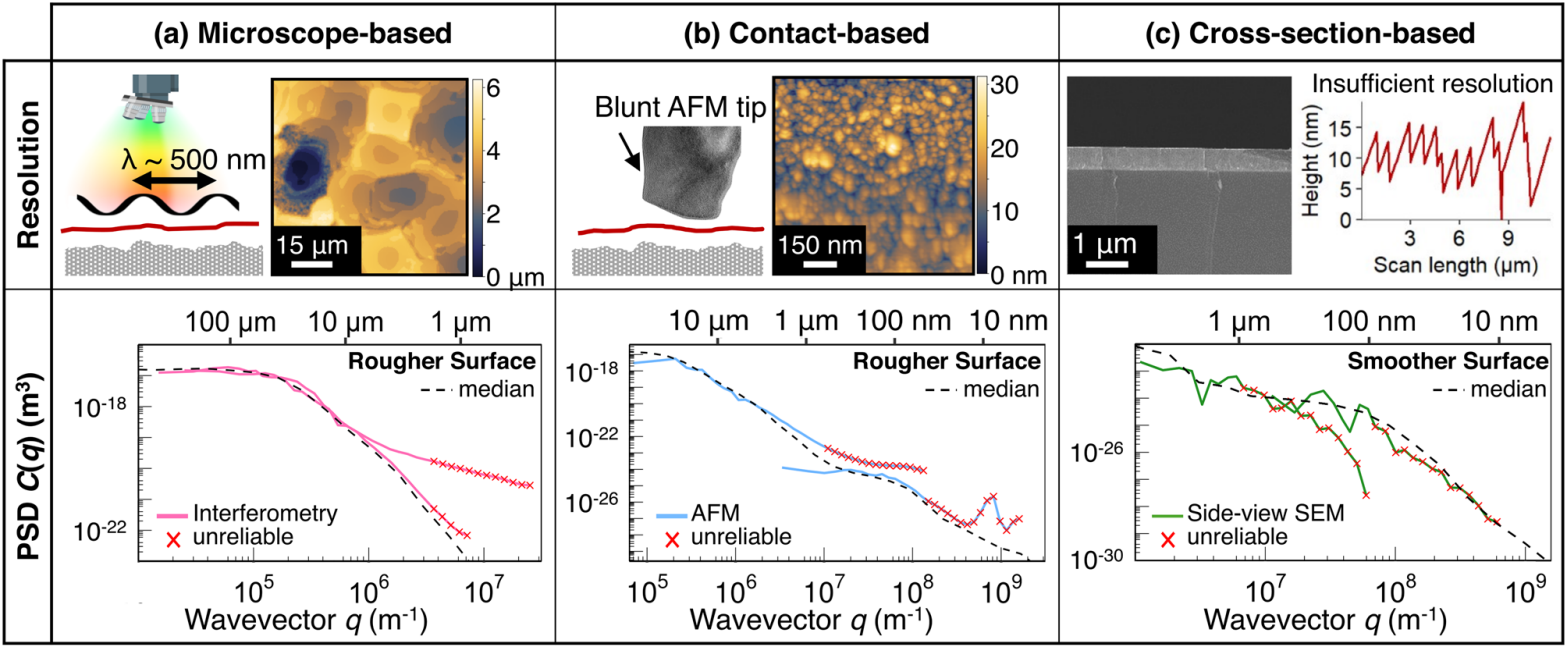
Lesson 2. A consistent statistical description emerges when lateral length scales are accounted for, and each technique is appropriately corrected for artifacts and resolution limits. Scale-dependent parameters enable the explicit correction or removal of unreliable portions of the topography, while leaving the reliable portions unaltered. When different techniques are corrected appropriately, then a more accurate result emerges. In the top row, a visual example of the technique is given for all three categories of techniques. **a** Microscope-based measurements can include unreliable data due to resolution limits or other known artifacts specific to the technique, such as diffraction effects near sharp edges of plateaus for interferometry. **b** Contact-based techniques like AFM include, for example, well-known rounding or apparent smoothening of topography features due to wearing of tip. **c** Similarly, for cross-section-based techniques such as side-view SEM, the vertical resolution is reduced at low magnification when the user is capturing a large scan length image of the Smoother Surface, which has only nanometer-scale features. The result is a pixelated trace of the surface profile

Each individual measurement simultaneously contains both reliable and unreliable data, as shown in Fig. 8. For example, a stylus profilometer tool can be set to collect data with a lateral point spacing of 10 nm, yet a 5-$\upmu$m end-radius on the needle will compromise some of the measured topography. At the small length scale, the measured “topography” will be dominated by instrument noise and also by “kinks” that appear when the tip cannot descend all the way into a narrow crevice. Therefore, while the lateral *pixel spacing* of the raw image may be on the nanometer scale, the lateral *resolution* is determined by the tip and is likely greater than 5 $\upmu$m.

Furthermore, this Challenge revealed that there appears to be some confusion on what is meant by “resolution” of a technique, as shown in Fig. 6b. Many groups reported lateral resolution of optical techniques that is smaller than the wavelength of the light used. This likely arises by quoting the manufacturers’ maximum “resolution” of the tool, which may be the vertical resolution, rather than the lateral resolution.

Yet, instead of dismissing whole measurements because of artifacts at certain scales, we can detect and eliminate unreliable portions within a measurement. Based on a metrology device’s physical principle, and the configuration used, each measurement can have unreliable data filtered out based on the estimated resolution of that measurement. For instance, probe-based techniques (including stylus profilometry and atomic force microscopy) can be corrected using a criterion [27, 58, 61] or, more simply, just cut off at the tip size. Microscope-based and cross-section-based techniques can be cut off according to an estimate of resolution, such as the diffraction limit of the imaging medium or, more accurately, a larger estimate based on the particular lens configuration.

More generally, the lessons from these measurements can be applied to standard roughness analysis, which already includes filtering. For example, in ISO 21920, [11] the S-filter removes smallest-scale “noise” by eliminating all contributions to topography at length scales below a cutoff set by the parameter $\lambda _\text {s}$. Also, for any reported roughness parameter (or R-parameter), the L-filter removes larger-scale “waviness” by eliminating all contributions to topography at length scales above a cutoff set by the parameter $\lambda _\text {c}$. However, this kind of one-size-fits-all filtering takes place in the background, is typically not fully understood by users, and is not customized to the measurement configuration. At a minimum, these two filter sizes must be reported alongside any reported roughness parameter. Better yet, those filter cutoffs can be manipulated to align with the factors discussed here. For example, $\lambda _\text {s}$ can be explicitly set to the resolution of the instrument. In the same way, the RMS height reported in this investigation would be equal to the standard parameter *R*q if $\lambda _\text {c}$ were set equal to the total scan length of the measurement. If filtering were to be used, then “filter length” would need to replace “scan length,” for instance in Fig. 5. However, the overall lessons and best-practices apply equally well when filtering is used.

#### Topography Best Practice 2: Compute and Report Scale-dependent Parameters

Ideally, surface topography would be expressed as a curve instead of a single number. Multi-scale descriptors such as the scale-dependent RMS height provide a fuller description of the surface than any scalar value. They easily facilitate the combination of multiple measurements into a single descriptor of the surface, and they readily expose artifacts within or between techniques, such as when topography is being “measured” on scales below the resolution limit of the instrument. The PSD and other scale-dependent parameters (such as the scale-dependent-roughness parameters (SDRPs) [63] and the height-difference autocorrelation function (ACF) [65]) also achieve these purposes. In cases where a single number is still preferred over a curve then, at a minimum, the relevant length-scale must be reported alongside the roughness metric.

### Lesson 3: A Single Number Cannot Describe a Surface

**Fig. 9:**
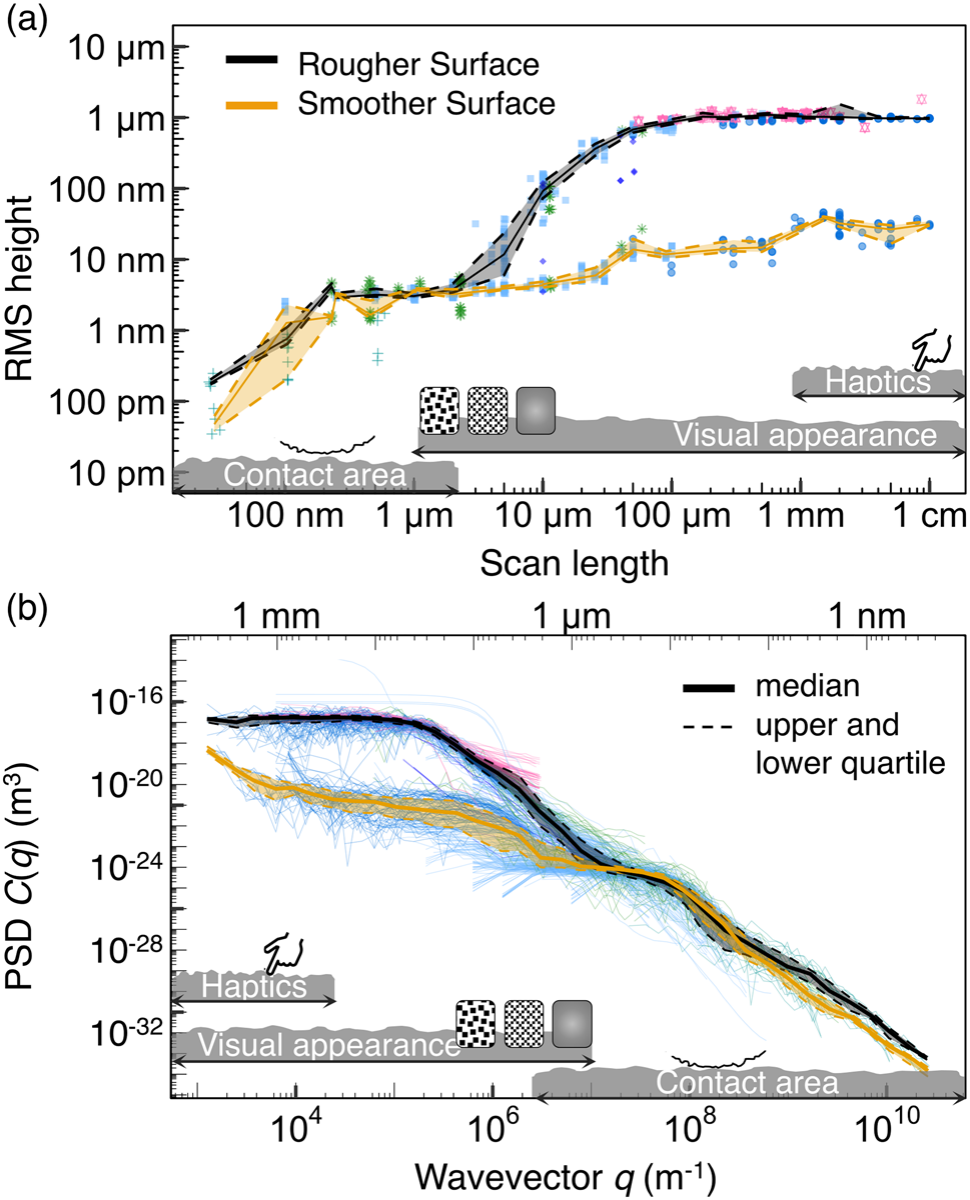
Lesson 3: A single number cannot describe a surface. **a** Root-mean-square height as a function of scan length. If filtering had been used, then “filter length” would replace “scan length” on the x-axis (as discussed in the main text). **b** Power spectral density (PSD) after correcting for extreme values and instrumental artifacts. These two plots enable scale-dependent comparisons between surfaces, which yields far more insight than can be provided by any single roughness metric

By working together as a community, this international collaboration has achieved the most comprehensive topography characterization ever performed. The complete and accurate description of both surfaces is shown in Fig. 9, both in the form of scale-dependent RMS height, as well as the power spectral density.

These complete surface measurements demonstrate that the topography of a surface cannot meaningfully be captured by any single number. Figure 9 demonstrates that the topography of the two samples differs at large scales, but that they have identical topography at scales below roughly 3 $\upmu$m. In originally naming the surfaces, we designated them as the “Rougher Surface” and the “Smoother Surface”, biased by their visual appearance. On a lateral scale of 100 $\upmu$m, the Rougher Surface has an RMS height of 900 nm and the Smoother Surface has an RMS height of 10.5 nm. However, on the scale of 1 $\upmu$m, both the Rougher Surface and the Smoother Surface have the same RMS height of 3 nm. This is precisely to be expected, given how these surfaces were made: the silicon wafers were intentionally varied in their pre-coating (large-scale) topography, but then both were coated with a thin layer of the identical CrN surface coating (which defines topography at the small scale). This does not imply that *all* surfaces are the same at small scales; indeed, prior experience demonstrates that they are not.

This scale-dependent similarity and difference means that these two surfaces will likely behave quite differently from each other in applications where the large-scale topography matters, such as sealing and leakage [66] or haptic properties [67, 68]. Yet they may behave identically in cases where nanoscale topography primarily governs performance, for example in certain biomedical applications [69]. In Fig. 9, we have indicated representative length scales that matter most for haptics [67, 68], visual appearance [70] and the true area of elastic contact [19–21].

Furthermore, the computation of any parameter, even a scale-dependent parameter, makes context-dependent and sometimes empirical choices, such as the precise imputation scheme for missing data (Appendix E2) or details on the choice of windowing for Fourier analysis (Appendix E3). While it is important to document the choices that were made in any calculation, a more robust solution is to always save and report the raw topography data. The raw data allows subsequent context-dependent analyses to be performed with differing methods, including corrections to errors made in prior analyses. In this investigation, all raw topography data has been shared and made publicly available, as described in the Data Availability section.

#### Topography Best Practice 3: Save, Analyze, and Report Raw Topography Measurements, not just Computed Parameters

The most common current practice in describing surfaces is to measure and report only roughness parameters; this investigation shows the value of saving, analyzing, and reporting the *raw* topography data. The use of raw topography data ensures that measurements from different contexts and different instruments can be meaningfully compared using the same analysis routines. When all of these disparate datasets are combined together, as is done in Fig. 9, then they can be used to reconstruct the true surface topography, in a way that is more accurate and comprehensive than any individual measurement or parameter can possibly be.

There are many ways to achieve these “Topography Best Practices”. For convenience, all can be easily implemented in the freely available, open-source, topography-analysis platform contact.engineering [27], which was used in the present analysis. Here, many different measurements can be combined into a single Digital Surface Twin that describes a sample, which can be shared with collaborators and, if desired, can also be published and referenced through a digital object identifier (DOI). This platform also implements the topography analysis calculations described here, and many others. Of course, these calculations can also be performed using a variety of other commercial and open-source software solutions, or manually calculated as described in this paper and the relevant references.

## Achievements and Outlook

This paper reports on what are possibly the two best-characterized samples of two different surfaces yet. This may well be an achievement in itself. The 153 authors on this paper made 2088 measurements, allowing us to show how much any individual measurement is prone to inconsistency and artifacts. Yet, when all are looked at together, and corrected accordingly, a consistent statistical picture of each surface emerges – a statistical description of what might be called the “true” topography.

The second achievement is the creation of a type of roughness standard. We envision that these well-characterized surfaces can be used, for example, in training, tool assessment, or for future investigation into topography or even surface properties. For anyone who measures surfaces in their work, we will continue shipping out samples, at least until the current supply is exhausted. Even those who did not participate in this Challenge should feel free to request samples at https://contact.engineering/challenge.

Finally, the lessons and best practices described in detail in the previous section are a third achievement. In summary, these best practices are: *(1) Combine multiple types of measurements of the same surface.* Even for a single surface, there were orders-of-magnitude variations in calculated parameters; we showed that comparing techniques is a robust way to catch and correct these. *(2) Compute and report scale-dependent parameters.* At a minimum, measurement and filter sizes should be reported alongside scalar metrics like $R$a; better yet, parameters should be presented as a function of scale, like the scale-dependent root-mean-square height reported here (Fig. 9). *(3) Save, analyze, and report raw topography measurements, not just computed parameters.* It is commonplace to describe topography using a single number; this investigation demonstrates the importance of saving, describing, and combining raw topography measurements for a more complete description of surfaces.

We envision that by following the three best-practices above for reporting topography, the community can move faster towards improvements in surface performance. For example, the move away from a simple value of $R$a and toward scale-dependent metrics will enable improved correlations with performance, eventually leading to predictive performance improvements. While we acknowledge that this Challenge examined only a limited selection of materials (CrN coated on rough and smooth wafers), we believe that the insights generated will generalize to the characterization of other hard-material surfaces. We hope that this additional knowledge and understanding about topography will advance the state of the art in the science and engineering of surfaces.

## Supplementary Information

Below is the link to the electronic supplementary material.

Supplementary file1 (PDF 8409 KB)

## Figures and Tables

**Fig. 10:**
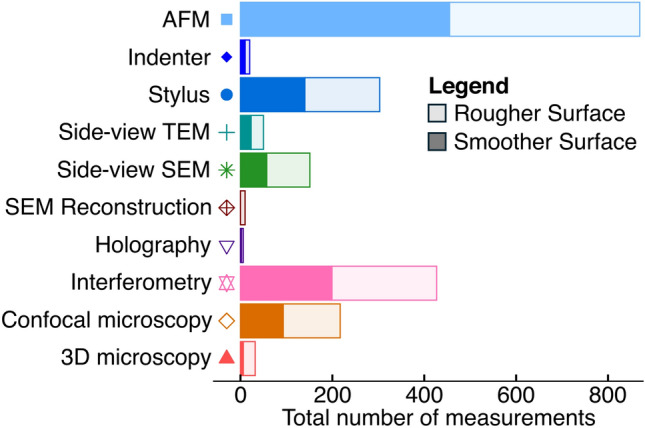
Number of measurements for each technique

**Fig. 11:**
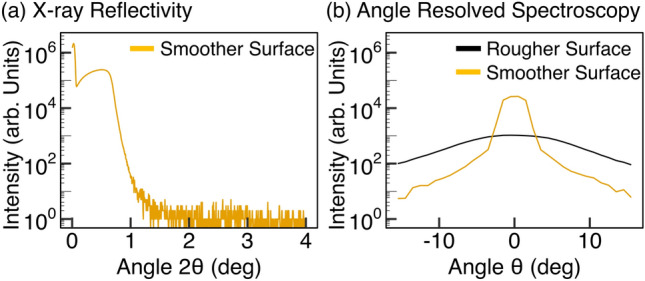
Results from scattering techniques. **a** For XRR, the raw data consists of the intensity of the scattered light versus scattering angle. **b** For ARS, the same plot is produced, but for a wider range of scattering angles. The XRR measurement was only taken on the Smoother Surface

## Data Availability

All data and analysis presented in this paper is available for download; we actively encourage its reuse and re-analysis. Specifically, we have created a repository containing all of the submitted measurements, as well as the reports (described in Section 3) that were submitted and where consent to publish was granted. This repository is available as a standalone download via 10.5281/zenodo.15341939. All data processing (such as tilt correction) and calculation of parameters (such as RMS height and PSD) was performed using contact.engineering, which can be accessed via web application at https://contact.engineering or downloaded open-source and run locally. In addition to the paper-wide data availability described above, each submitting group was encouraged (but not required) to publish the digital surface twin of their submissions on the website https://contact.engineering. To be clear, each group was *required* to create *private* digital surface twins and to share these with the organizers of this Challenge — this was the mechanism of submission. However, they were only *encouraged* to make these digital surface twins *public*. The publication links to all of the individual submissions can be found at https://contact.engineering/challenge.

## Appendix A: Sample Fabrication

All samples consisted of CrN deposited on two different kinds of silicon wafers. To create samples of the Smoother Surface, the CrN was deposited on prime-grade polished silicon wafers. These were single-side-polished wafers, with the coating applied to the polished side (often called the “frontside”).

To create samples of the Rougher Surfaces, we applied reactive-ion-etching to the unpolished “backsides” of a separate set of single-side-polished wafers, and then applied the same coating. The processing of the wafer backsides can vary across manufacturers, but most commonly includes grinding and lapping to reduce the marks from the wafer saw, followed by isotropic etching. In our case, we subjected the backside samples to an additional reactive-ion-etch (RIE) step to impart extra topography to the ultra-flat plateaus of the as-received samples. Specifically, the RIE was performed in a Trion Phantom III LT reactive ion etcher on prime single-side-polished Si wafers. The reaction chamber was pumped down to 50 mTorr. The reaction mixture of 25 sccm of oxygen (O$_2$) and sulfur hexafluoride (SF$_6$) was introduced into the chamber. An RF of 100 W was applied to the chamber once the chamber pressure and gas flow rates stabilized. The samples were exposed to the reaction mixture for 1 min, at which point the chamber was back-filled with nitrogen until it was returned to atmospheric pressure. The wafers were etched two at a time, set in the center of the chamber.

The CrN was deposited with bipolar high-power impulse magnetron sputtering (HiPIMS, performed by IBC Materials, Lebanon, IN). A 3-hour deposition was performed in a single batch for all wafers, after a 15-min Cr-ion etch to pre-treat the surface. The HiPIMS pulse was 100 $\upmu$s at 265 Hz, followed by a 100-V positive pulse. The chamber was maintained at a pressure of 8.5 mTorr, and the substrate was biased with 50 V DC. The coating was deposited on all wafers in a single processing batch to eliminate the potential for batch-to-batch variation.

## Appendix B: Data Formats

Of the ten different techniques that are shown in Fig. 2, Fig. 10 shows the number of individual submissions that were received for each type. Furthermore, Table 1 contains a list of all of the different data formats in which we received submissions. We attempted to parse the native (often proprietary) format of the respective instrument, to avoid post-processing by third-party software. The only standardized format for topography data that we are aware of is the *XML surface profile* (.x3p) whose metadata are described in ISO 5436-2 [72]. For many formats, formal documentation exists in instrument manuals. This is beneficial, but often those manuals are not openly available to the community and are typically distributed at the manufacturer’s discretion. In many cases, informal documentation has been compiled by researchers, and there exist reference implementations for readers. All of the readable formats submitted to the present Challenge are listed in Table 1.

Around one-third of submissions were received in generic text formats, as MATLAB files or as Gwyddion [52] data files. These generic formats were common for home-built instruments, but also measurements with instruments whose data files we could not read directly, and where manufacturers did not provide documentation upon request (e.g., for Filmetrics’ native formats).

**Table 1:** File formats of submitted datasets. $^1$Format has metadata in some human-readable text format (including markup languages like XML). $^2$Format uses a documented self-describing container that simplifies reading the data. $^3$Text formats come in many flavors, some including metadata in the header of the files. All listed formats can be read by the open-source software packages Gwyddion (GPL license, Ref. [52]) and SurfaceTopography, which is part of Contact.Engineering (MIT license, Ref. [27]). Abbreviations: HDF5 – Hierarchical Data Format version 5; IBW – Igor Binary Wave; TIFF – Tagged Image File Format; XML – Extensible Markup Language

Name	File extensions	Standardized	Documented	Human readable	Submissions
**Standardized formats**					
XML surface profile	.x3p	ISO5436-2	Yes	Yes	25
**Accessible formats used by instrument manufacturers**					
Alicona Imaging	.al3d	No	Yes	No	6
Dektak OPDx	.opdx	No	No	No	337
Digital Instruments	.001, .002, .spm	No	Partially	Partially$^1$	274
Digital Surf SUR	.sur	No	Yes	No	82
Igor binary wave	.ibw	IBW$^2$	Yes	No	218
Image Metrology SPIP	.bcr, .bcrf	No	Yes	Partially$^1$	150
JPK image scan	.jpk	TIFF$^2$	Yes	No	40
Keyence VK3/4	.vk3, .vk4	No	Partially	No	64
Keyence VK6/7	.vk6, .vk7	ZIP$^2$	Partially	No	22
Keyence ZON	.zon	ZIP$^2$	No	Partially (XML)$^1$	4
Microprof FRT	.frt	No	No	No	2
Nanosurf easyScan	.ezd, .nid	No	Yes	Partially$^1$	69
Olympus LEXT	.lext	TIFF$^2$	No	Partially (XML)$^1$	28
Olympus Packed OIR	.poir	ZIP$^2$	No	Partially (XML)$^1$	24
Park Systems TIFF	.tiff	TIFF$^2$	Partially	No	84
Sensorfar SPM	.plu	No	Yes	No	12
Sensorfar SPM XML	.plux	ZIP$^2$	Yes	Partially (XML)$^1$	12
WSxM	.stp, .top	No	No	No	20
Wyko OPD	.opd	No	No	No	154
Zygo DATX	.datx	HDF5$^2$	No	Partially$^1$	108
Zygo Metropro DAT	.dat	No	Yes	No	28
**Generic formats**					
MATLAB	.mat	No	Yes	No	54
Numpy NPY	.npy	No	Yes	No	4
Text$^3$	.asc, .txt, .xyz	-	-	Yes	380
**Accessible formats used only by metrology software**					
Gwyddion	.gwy	No	Yes	No	227

Overall, our impression is that more emphasis should be placed on core principles of *Open Science* [53] in surface metrology. We have implemented support for all file formats in Table 1 for the Surface-Topography Challenge into contact.engineering [27]; however, this effort would not be required if topography measurements were interoperable. Such technical hurdles to open and read data are binding valuable resources, especially considering that the formats listed in Table 1 represent only a subset of the formats used by topography-measurement instruments. We urge the community to agree on a clearly standardized format for reporting topography data right from the point of measurement, such as X3P, and to request such standardization in the procurement of instruments. An intermediate solution is to agree on human-readable metadata (JSON, XML) embedded in standardized container formats (e.g., HDF5) that some of the more accessible formats in Table 1 already use.

## Appendix C: Results and Discussion on Scattering Techniques

As described in the main text, scattering techniques cannot produce a topographic map and therefore generate data that is qualitatively different from all other submitted measurements. The raw data for XRR and ARS is displayed in Fig. 11 and takes the form of intensity as measured at each scattering angle (which can be converted to a scattering vector through the grating equation).

It is possible, though not straightforward, to extract common roughness parameters such as the root-mean-square height of the interface or the power spectral density from the raw data. The most commonly applied formulations are those of Rayleigh-Rice [70, 73–75] and Kirchoff [76]. In general, the former is considered applicable to smoother surfaces; the latter to rougher surfaces [49].

The group that submitted XRR data relied on the in-built analysis models of the measuring instrument, which reported an *R*q of 4.386 nm. This can be compared with the results of Fig. 9a, under the assumption of a “scan length” corresponding to the whole sample, approximately 1 cm. This compares reasonably favorably with the consensus value, of order 10 nm at this scan length. However, the software reports this value as *R*q, which implies that it is a filtered parameter, and therefore it is not necessarily equivalent to the RMS height reported in this investigation.

The group that submitted ARS data provided an example of using Rayleigh-Rice theory to compute the PSD, as described in Ref. [77]. However, they acknowledged that the computation of the PSD requires knowledge of the surface polarization coefficient, and other material properties, many of which are not known in many real-world cases of general topography characterization. Furthermore, as discussed in Ref. [49], it can be shown that multiple PSDs can produce the same scattering profile, therefore it is not typically possible to extract precise values.

For these reasons, the ARS and XRR submissions were not compared with other techniques, for instance in Fig. 9. However, these are important techniques in surface characterization, and therefore merit inclusion in this investigation. They are especially useful in cases where the material properties of the measured surface are very well characterized, or for relative comparisons across similar materials that vary only in their topography.

## Appendix D: Statistical Parameters and Detrending

The simplest statistical roughness parameter is the root-mean-square deviation $h_\text {rms}$ of the height *h*(*x*) from some reference line *t*(*x*),

D1 $$\begin{aligned} h_\text {rms}^2 = \frac{1}{L}\int _0^L\textrm{d}x\, \left[ h(x)-t(x)\right] ^2 \equiv \left\langle \left[ h(x)-t(x)\right] ^2\right\rangle _x, \end{aligned}$$

where *L* is the scan length and $\langle \cdots \rangle _x$ a short-hand for the average over *x* in Eq. (D1). We interpret topographic maps (area scans) as arrays of consecutive profiles; this enables the identical analysis to be applied to data from instruments that yield both line scans and those that yield area scans. If evaluated on a discrete set of equally spaced grid points (see inset to Fig. 3b), then interpreting the height as piecewise constant yields

D2 $$\begin{aligned} h_\text {rms}^2 = \frac{1}{N}\sum _{k=1}^N \left[ h_k-t(x_k)\right] ^2, \end{aligned}$$

where $x_k$ is the location of the *k*-th grid point and $h_k$ the (measured) height at that point. This is the expression also found in standards for *R*q [10, 11], but those standards often mandate or recommend the application of filters, whereas $h_\text {rms}$ is computed on the unfiltered data. For line scans that are measured on a nonuniform grid (as typically obtained in cross-section-based techniques), we interpolate linearly between grid points and compute the RMS height of this interpolated profile using Eq. (D1).

Finding an optimal reference line *t*(*x*) to correct for misalignment, instrument drift, and other undesired artifacts remains a somewhat open question, as the best choice certainly depends on the nature of the artifact, the instrument, and the roughness—e.g., whether or not it contains a deterministic component. We chose *t*(*x*) to be the function that minimizes $h^2_\text {rms}$. Specifically, we use a quadratic function $t(x)=z_0 + \alpha x + \beta x^2$ and search for the parameters $z_0$, $\alpha$ and $\beta$ that minimize $h_\text {rms}$. This procedure is commonly called detrending or curvature correction. Note that for topographic maps we detrend by minimzing $h^2_\text {rms}$ computed for the map,

D3 $$\begin{aligned} h_\text {rms}^2 = \frac{1}{L_xL_y}\int _0^{L_x}\textrm{d}x\int _0^{L_y}\textrm{d}y\, \left[ h(x,y)-t(x,y)\right] ^2, \end{aligned}$$

where $t(x,y)=z_0 + \alpha _x x + \alpha _y y + \beta _{xx} x^2 + \beta _{yy} y^2 + \beta _{xy} xy$ is now a trend plane and we again interpret a discrete data set as piece-wise constant. The final $h_\text {rms}$ values reported in this paper are computed from averages of $h_\text {rms}^2$ over consecutive lines in area scans. This is compatible with the computation of the PSD (see next section) that is also carried out in a line-by-line fashion.

We note that detrending introduces artifacts in scale-dependent statistical measures, particularly in the PSD described in Appendix E. The reference line *t*(*x*) removes long-wavelength contributions to the topography, which can cause a visible downtick in the PSD at small wavevectors. We do not correct for these long-wavelength artifacts, but they are visible in our PSDs (e.g., Fig. 9). More information on this type of artifact can be found in Ref. [78].

Some measurements, mainly optical methods, can have missing data. We simply exclude missing data points from the averages that are described in Eqs. (D1) to (D3).

## Appendix E: Power Spectral Density (PSD)

To characterize the scale-dependence of topographic features within individual measurements we employ the PSD *C*(*q*). The PSD is commonly used in surface metrology. *C*(*q*) is often the direct input to analytic theories of contact, most notably the theory of Persson for the probability distribution of pressure in the contact [12, 15, 16]. Additionally, the PSD allows various scalar roughness descriptors to be computed from it, in particular the root-mean-square height of Appendix D, but also RMS slope and RMS curvature (plus the RMS of all higher-order derivatives), which should all be regarded as explicitly depending on scale. Unfortunately, the definition of *C*(*q*) is not unique, because the Fourier transform, which is needed to define it, is itself defined only up to a multiplicative factor that may or may not have a unit. As a consequence, height or other spectra reported by different groups cannot always be directly compared to each other.

### 1. Convention for Fourier Transforms and Sums

The prefactor in the definition of the Fourier transform is arbitrary, except that it must differ from zero and when transforming back the original function must be restored (which typically means a factor of $2\pi$ overall). In this work, we use the convention

E1 $$\begin{aligned} \tilde{h}(q_n) \equiv \int _L h(x) e^{-\textrm{i} q_n x}\,\textrm{d}x, \end{aligned}$$

where *L* is the domain of integration, $q_n = 2\pi n/L$ with $n \in \mathbb {Z}$, while *h*(*x*) is the real-valued height function, already containing the trend corrections described in Appendix D. In the context of topography, the coordinate system should be chosen such that the mean height *h*(*x*) vanishes, which is always fulfilled after the detrending described in Appendix D. In practice, *h*(*x*) is not known as a continuous function, but rather is defined on a grid with mesh-size $\Delta x$. In this case, spatial integrals like that in Eq. (E1) are approximated with (Riemann) sums by replacing $\int _L I(x) \textrm{d}x$ with $\sum _n I(x_n) \Delta x$, where *I*(*x*) is the integrand and $x_n = n \Delta x$.

The convention of Eq. (E1) yields the inverse Fourier transform

E2 $$\begin{aligned} h(x) = \sum _{n=-\infty }^\infty \frac{\Delta q}{2\pi }\, \tilde{h}(q_n) e^{\textrm{i} q_n x} \end{aligned}$$

with $\Delta q = 2\pi /L$ and $q_n = n\Delta q$. Theoretical or analytical treatments are mostly formulated in terms of Fourier integrals rather than sums,

E3 $$\begin{aligned} h(x) = \frac{1}{2\pi }\int _{-\infty }^\infty \tilde{h}(q) e^{\textrm{i} qx}\, \textrm{d}q, \end{aligned}$$

where $\Delta q/(2\pi )$ was substituted with $\textrm{d}q/(2\pi )$ and where $\tilde{h}(q_n)$ or rather the expectation value of its (square) magnitude is assumed to evolve smoothly (except at isolated points such as wavevectors associated with cut-offs or roll-offs) with the index *n*. We only describe one-dimensional transforms here, as we interpret each area scan as a series of line scans, such that they can be combined with data from instruments that only measure line scans.

### 2. Imputation of missing data

While missing data can simply be ignored for the computation of $h_\text {rms}^2$ (which is the average square deviation from the centerline *t*(*x*)), we cannot simply ignore missing data in Eq. (E1) or its two-dimensional generalization. Ignoring the respective term in Eq. (E1) amounts to setting the value at $h(x_n)$ to zero, which has consequences for the spectrum. There are two strategies to circumvent this problem: Filling-in (imputation) of missing data points or computing the real-space autocorrelation function [22] and converting it into a PSD. We here use the former strategy, the latter is described in detail in Ref. [79].

We fill in missing data points using a method described in Ref. [27]. Briefly, we solve the Laplace equation, $\nabla ^2 h_\text {imp}(x,y)=0$, within the missing region, subject to the boundary condition that $h_\text {imp}(x,y)=h(x,y)$ on the boundary, using a canonical second-order central-differences scheme. This fills each island of missing values with a harmonic function, which has the property that the RMS gradient

E4 $$\begin{aligned} g_\text {rms}^2 = \int _{A_\text {imp}} \textrm{d}x\textrm{d}y\, \left| \nabla h(x,y)\right| ^2 \end{aligned}$$

is minimal over the filled-in region $A_\text {imp}$. This construction has the property that the mean of the interpolated field is equal to the mean of the height over the edge (by virtue of the mean value property of harmonic functions). For line scans, this is simply a linear interpolation of missing data, which means the imputation is exact for linear fields. For area scans, this leads to well-behaved interpolations without jumps, even in cases where large patches are missing

### 3. Windowing

Equation (E1) and its generalization to two spatial dimensions should only be applied directly to periodically repeated functions. For any non-periodic surface, caution has to be taken, in particular when the height spectra that are deduced from *h*(*q*) serve as input for further data processing or modeling. The reason is that a representation of a surface height in terms of a Fourier sum implicitly treats the surfaces as periodic and continuously repeating. This makes *h*(*x*, *y*) discontinuous at the boundaries between the original domain and its periodically repeated neighbor. This discontinuity is artificial, and would not have appeared if the height profile had been measured over a larger domain. As a consequence, RMS gradients or curvatures can be grossly exaggerated when Eq. (E1) is used on experimental data without further processing. The solution to this issue is well-studied by the signal processing community: A window function *w*(*x*) is applied to the heights, such that the Fourier transform is computed on the windowed function $h_\text {w}(x)=w(x)h(x)$ rather than the bare *h*(*x*). We use a Hann window for all PSDs reported in this paper.

Windowing changes the effective topography from *h*(*x*) to $h_\text {w}(x)$, but it should affect the deduced statistical properties of a stationary random process in the least possible way. There is no unique way to achieve this, as the correction depends on which statistical property should remain unaffected. We here require that the mean-square height of the windowed topography

E5 $$\begin{aligned} \left\langle h_\text {w,rms}^2 \right\rangle _\text {e} = \left\langle \left\langle h_\text {w}^2(x) \right\rangle _x \right\rangle _\text {e} = \left\langle \left\langle w^2(x) h^2(x)\right\rangle _x \right\rangle _\text {e} \end{aligned}$$

be equal to $\langle h_\text {rms}^2 \rangle _\text {e}$ when averaged over (ideally infinitely) many independent random realizations for *h*(*x*), sometimes called a disorder or ensemble average, here indicated by $\langle \cdots \rangle _\text {e}$. The idea is that this condition yields minimally changed values for *C*(*q*). One way to achieve this is to normalize the window function such that

E6 $$\begin{aligned} \left\langle w^2(x) \right\rangle _x = 1, \end{aligned}$$

which ensures that $\langle h^2(x) \rangle _\text {e}=\langle h_\text {rms}^2 \rangle _\text {e}$. Another way to keep $h_\text {rms}$ unaffected by windowing is to apply a post-hoc correction to the amplitude of $h_w(x)$. In all calculations reported in the paper, we use the normalized window function.

### 4. Power spectral density

We are now in a position to introduce the power spectral density, which we define here using the most common definition

E7 $$\begin{aligned} C(q) \equiv \frac{1}{L} \left\langle \left| \tilde{h}(q) \right| ^2 \right\rangle _\text {e}. \end{aligned}$$

Note that the choice of the multiplicative factor in the Fourier transform does affect the precise numerical values of *C*(*q*). And correction factors may need to be applied when comparing between calculations that use different conventions. This topic is discussed further in Ref. [58]. Furthermore, as mentioned previously, the PSD of area scans is computed using the disorder average $\langle \cdots \rangle _\text {e}$ over all adjacent horizontal lines ( e.g., the fast-scan direction for scanning probe techniques).

One of the most common topography descriptors is the mean-square height. It can be deduced from the PSD using

E8 $$\begin{aligned} h_\text {rms}^2 = \left\langle h^2(x) \right\rangle _x = \frac{1}{\pi }\int _{0}^\infty C(q) \, \textrm{d}q \end{aligned}$$

assuming that the mean height vanishes. Note that a spatial average in addition to a disorder average is assumed to be taken in Eq. (E8).
