# Supplementary material for: The Surface-Topography Challenge: A Multi-Laboratory Benchmark Study to Advance the Characterization of Topography
Source: Tribol Lett. 2025 Jul 26;73(3):110. doi: 10.1007/s11249-025-02014-y (PMC12296861; doi:10.1007/s11249-025-02014-y)
Supplement: Supplementary file 1 — Supplementary file1 (PDF 8409 KB) [file 11249_2025_2014_MOESM1_ESM.pdf]

Supporting Information for the paper titled:

The Surface-Topography Challenge: A Multi-Laboratory Benchmark Study to Advance the Characterization of Topography

As described in the main text, the corresponding author from each group (research group, lab, or company) was asked to submit a supplementary-information form with relevant information about their group. These forms are self-reported, and the accuracy of all information is verified by the submitter. An example of the blank template that was sent to all participants is included as the final page of the present document. These forms are reproduced (with yellow highlighting removed) in the following pages.

As shown in the fields below, each group was invited to include information about all authors from their groups, all relevant funding information, and a small amount of key information about each technique that was included in their submission. Note that there was not sufficient space in this document to include a comprehensive description of each method, technique, and parameter-set used.

Participants were encouraged to include *full* details of their submissions in the "Description" field of their submission on the Contact.Engineeering site. All measurements for a single sample were uploaded as a single *digital surface twin* and therefore sample-wide details (such as cleaning and handling procedures) could be described in the Description of the digital surface twin. By contrast, the parameters that are unique to each measurement (instrument used and instrument parameters) could be described in the Description fields of the individual measurements.

All participants were encouraged (though not required) to publish their individual contributions on the contact.engineering site. Doing so would make all details of their submissions publicly available.

Once again, the remainder of this Supplemental-Information document contains all individual forms from each group. These forms are published *verbatim* in the following pages, without any text modification.

AUTHOR AND TECHNIQUE INFORMATION FOR THE SURFACE-TOPOGRAPHY CHALLENGE

Submit to: SurfaceTopographyChallenge@gmail.com ***(***Deadline: 29 Feb 2024***)***

- We created this template to standardize the information we get from each group.
- Information submitted will be published *verbatim* in the Supplementary Information section.
- ***Please fill out all yellow-highlighted fields to the best of your ability.***

AUTHORSHIP INFORMATION (*Text only, please. We cannot accept citations in this document.*)

- **Author information (please ONLY include authors that directly contributed)**
  - **Author 1:** Fang, Lu
    - **ORCID ID:** 0000-0002-0175-0802
    - **Address, Line 1:** Mechanical Engineering and Applied Mechanics, University of Pennsylvania
    - **Address, Line 2:** 220 S. 33rd St., Philadelphia, PA, USA, 19104-6315
  - **Author 2:** Yuan, Li
    - **ORCID ID:** 0009-0001-2791-0923
    - **Address, Line 1:** Mechanical Engineering and Applied Mechanics, University of Pennsylvania
    - **Address, Line 2:** 220 S. 33rd St., Philadelphia, PA, USA, 19104-6315
  - **Author 3:** LaMascus, Parker
    - **ORCID ID:** 0000-0002-7945-849X
    - **Address, Line 1:** Mechanical Engineering and Applied Mechanics, University of Pennsylvania
    - **Address, Line 2:** 220 S. 33rd St., Philadelphia, PA, USA, 19104-6315
  - **Author 4:** Carpick, Robert W.
    - **ORCID ID:** 0000-0002-3235-3156
    - **Address, Line 1:** Mechanical Engineering and Applied Mechanics, University of Pennsylvania
    - **Address, Line 2:** 220 S. 33rd St., Philadelphia, PA, USA, 19104-6315

  - *(NOTE: For three or more authors, please copy/paste additional Author lines)*

- **Funding information, if any** *(how it should appear in the acknowledgements)*
  - **Funder:** National Science Foundation; **Grant number: CMMI-2041662**
  - **Funder:** National Science Foundation; **Grant number: CHE-2303044**
  - **Funder:** Department of Energy; **Grant number: DE-EE0010211**

    - *(NOTE: For more than one funder, please copy/paste additional Funding lines)*

DESCRIPTION OF TECHNIQUES USED FOR DATA COLLECTION

(For reproducibility purposes, the publishing journal requires that we specify all details of each technique used. If you have any questions or concerns, contact SurfaceTopographyChallenge@gmail.com)

- **Number of techniques used:** 3

TECHNIQUE 1: [NOTE: Please copy/paste this section and repeat for each technique used]

(*Text only, please. We cannot accept citations in this document.*)

- **Common name of technique:** Confocal Microscopy
- **Type of technique:** Confocal laser scanning microscope
- **Instrument model:** Keyence VK X-3000
- **Tip size (if known):** N/A
- **Expected maximum lateral resolution:** 1 nm
- **Data post-processing:** N/A
- **Sample preparation, if any:**
  - Sonicate the samples with isopropanol for 10 mins
  - Blow dry the samples with air
  - Scan two different locations on each sample with different magnifications (5X, 10X, 20X, 50X, and 150X)

AUTHOR AND TECHNIQUE INFORMATION FOR THE SURFACE-TOPOGRAPHY CHALLENGE

Submit to: SurfaceTopographyChallenge@gmail.com ***(***Deadline: 29 Feb 2024***)***

TECHNIQUE 2: [NOTE: Please copy/paste this section and repeat for each technique used]

(*Text only, please. We cannot accept citations in this document.)*

- **Common name of technique:** Atomic Force Microscopy
- **Type of technique:** Atomic force microscope
- **Instrument model:** Asylum MPF3D
- **Tip size (if known):** 10 nm
- **Expected maximum lateral resolution:** 10~100 nm
- **Data post-processing:** Correction procedure (in Gwyddion):
  - Level data by mean plane subtraction.
  - Add masks to exclude chunks on the surface for corrections and statistical analysis.
  - Align rows using 0-order polynomial.
  - Shift minimum value to zero.
  - Set color range to enhance image contrast.
- **Sample preparation, if any:** N/A
  - No cleaning was performed on the samples before taking AFM images.

TECHNIQUE 3 (if applicable): [NOTE: Please copy/paste this section and repeat for each technique used]

(*Text only, please. We cannot accept citations in this document.)*

- **Common name of technique:** White Light Interferometry
- **Type of technique:**white light interferometer chromatic aberration
- **Instrument manufacturer:** Zygo
- **Instrument model:** NewView 6300
- **Tip size (if known):** n/a
- **Expected maximum lateral resolution:** 200-500 nm
- **Data post-processing:** Correction procedure (in Gwyddion):
  - Add masks to interpolate outliers, if needed
  - Level data by mean plane subtraction
  - Row-by-row polynomial subtraction (order 2) on samples A16-A17 to remove a curvature artifact
- **Sample preparation, if any:** Cleaned gently with N_2 airgun.

AUTHOR AND TECHNIQUE INFORMATION FOR THE SURFACE-TOPOGRAPHY CHALLENGE

Submit to: SurfaceTopographyChallenge@gmail.com ***(***Deadline: 29 Feb 2024***)***

- We created this template to standardize the information we get from each group.
- Information submitted will be published *verbatim* in the Supplementary Information section.
- ***Please fill out all yellow-highlighted fields to the best of your ability.***

AUTHORSHIP INFORMATION (*Text only, please. We cannot accept citations in this document.)*

- **Author information (please ONLY include authors that directly contributed)**
  - **Author 1:** Laux, Patrick

    - **ORCID ID:** 0009-0003-0127-082X
    - **Address, Line 1:** Fraunhofer Institute for Physical Measurement Techniques IPM
    - **Address, Line 2:** Georges-Köhler-Allee 301, 79110 Freiburg, Germany

DESCRIPTION OF TECHNIQUES USED FOR DATA COLLECTION

(For reproducibility purposes, the publishing journal requires that we specify all details of each technique used. If you have any questions or concerns, contact SurfaceTopographyChallenge@gmail.com)

- **Number of techniques used:** 1

TECHNIQUE 1: [NOTE: Please copy/paste this section and repeat for each technique used]

(*Text only, please. We cannot accept citations in this document.)*

- **Common name of technique:** Digital Holography
- **Type of technique:** Holographic measurement
- **Instrument manufacturer:** Fraunhofer IPM, Germany
- **Instrument model:** HoloTop
- **Tip size (if known):** Does not apply.
- **Expected maximum lateral resolution:** Optical resolution 6.4 µm (1.9 µm pixel sampling)
- **Data post-processing:** Multiple wavelength digital holography with temporal phase shifting is used (For further information see https://doi.org/10.37188/lam.2021.015). To reduce noise in the measurement data and improve the object reconstruction we use a moving average filter kernel with a size of 3 px during reconstruction. The pixel size is 1.90 µm. The process is described in more detail in https://doi.org/10.1364/AO.48.0000H1.
- **Sample preparation, if any:**

AUTHOR AND TECHNIQUE INFORMATION FOR THE SURFACE-TOPOGRAPHY CHALLENGE

Submit to: SurfaceTopographyChallenge@gmail.com ***(***Deadline: 29 Feb 2024***)***

- We created this template to standardize the information we get from each group.
- Information submitted will be published *verbatim* in the Supplementary Information section.
- ***Please fill out all yellow-highlighted fields to the best of your ability.***

AUTHORSHIP INFORMATION (*Text only, please. We cannot accept citations in this document.)*

- **Author information (please ONLY include authors that directly contributed)**
  - **Author 1:** Cai, Wanhao
    - **ORCID ID:** 0000-0002-3466-8530
    - **Address, Line 1:** School of Food Science and Engineering, Engineering Research Center of Bio-process (Ministry of Education), Hefei University of Technology
    - **Address, Line 2:** Tunxi Road. 193, 230009 Hefei, PR China
  - **Author 2:** Balzer, Bizan N.
    - **ORCID ID:** 0000-0001-6886-0857
    - **Address, Line 1:** Institute of Physical Chemistry, Cluster of Excellence *liv*MatS @ FIT - Freiburg Center for Interactive Materials and Bioinspired Technologies and Freiburg Materials Research Center (FMF), University of Freiburg
    - **Address, Line 2:** Albertstr. 21, 79104 Freiburg, Germany

- **Funding information, if any** *(how it should appear in the acknowledgements)*
  - **Funder:** Deutsche Forschungsgemeinschaft (DFG, German Research Foundation); **Grant number:** Germany's Excellence Strategy – EXC-2193/1 – 390951807
  - **Funder:** Deutsche Forschungsgemeinschaft (DFG, German Research Foundation); **Grant number:** HU 997/13-1

DESCRIPTION OF TECHNIQUES USED FOR DATA COLLECTION

(For reproducibility purposes, the publishing journal requires that we specify all details of each technique used. If you have any questions or concerns, contact SurfaceTopographyChallenge@gmail.com)

- **Number of techniques used:** 2 (2 different AFMs)

TECHNIQUE 1: [NOTE: Please copy/paste this section and repeat for each technique used]

(*Text only, please. We cannot accept citations in this document.)*

For samples:

- **Common name of technique:** AFM
- **Type of technique:** atomic force microscope, intermittent-contact mode (AC mode)
- **Instrument manufacturer:** Asylum Research, an Oxford Instruments Company, USA
- **Instrument model:** Cypher ES
- **Tip size (if known):** 5 nm radius (nominal value), model: Scout 70 RAl (NuNano, Bristol, UK)
- **Expected maximum lateral resolution:** 5 nm
- **Data post-processing:**
  - For original ibw files: none, we indicated during the upload process: Filters: Detrending - *Remove tilt*, Undefined/missing data - *Do not fill undefined data points*
  - For Gwy files: the height data of both trace and retrace images were analyzed with Gwyddion Free SPM analysis software [Nečas, D.; Klapetek, P., Gwyddion: an open-source software for SPM data analysis. *Cent. Eur. J. Phys.* **2012,** *10*, 181-188.]. The images were evaluated using the following tools: *align rows* via the polynomial method (degree: 5) and *fix zero*. Then, the root mean square (RMS) roughness of the images was calculated via the *statistical quantities tool*. If the images show dust particles, the following additional steps were performed: after using *mask of outliers* (masking all data values exceeding the confidence interval of 3 sigma), *align rows* via the polynomial method (degree: 5) and *fix zero* were applied.
- **Sample preparation, if any:** All the samples were rinsed extensively with pure H_2O (Purelab Chorus 1, Elga LabWater, Celle, Germany, 18.2 MΩ cm) and dried with N_2, then used immediately for the AFM imaging.
- **Additional imaging parameters:** in air, 25°C, 1024×1024 pixels, a scan rate of 1 Hz, scan angle of 90° (fast scan direction perpendicular to the cantilever axis) and scan sizes of 30×30, 10×10, 5×5, 1×1 μm^2, respectively.

AUTHOR AND TECHNIQUE INFORMATION FOR THE SURFACE-TOPOGRAPHY CHALLENGE

Submit to: SurfaceTopographyChallenge@gmail.com ***(***Deadline: 29 Feb 2024***)***

TECHNIQUE 2: [NOTE: Please copy/paste this section and repeat for each technique used]

(*Text only, please. We cannot accept citations in this document.)*

For samples:

- **Common name of technique:** AFM
- **Type of technique:** atomic force microscope, intermittent-contact mode (tapping mode)
- **Instrument manufacturer:** Park Systems, Korea
- **Instrument model:** XE7
- **Tip size (if known):** 5 nm radius (nominal value), model: Scout 70 RAl (NuNano, Bristol, UK)
- **Expected maximum lateral resolution:** 5 nm
- **Data post-processing:**
  - For original ibw files: none, we indicated during the upload process: Filters: Detrending - *Remove tilt*, Undefined/missing data - *Do not fill undefined data points*
  - For Gwy files: the height data of both trace and retrace images were analyzed with Gwyddion Free SPM analysis software [Nečas, D.; Klapetek, P., Gwyddion: an open-source software for SPM data analysis. *Cent. Eur. J. Phys.* **2012,** *10*, 181-188.]. The images were evaluated using the following tools: *align rows* via the polynomial method (degree: 5) and *fix zero*. Then, the root mean square (RMS) roughness of the images was calculated via the *statistical quantities tool*. If the images show dust particles, the following additional steps were performed: after using *mask of outliers* (masking all data values exceeding the confidence interval of 3 sigma), *align rows* via the polynomial method (degree: 5) and *fix zero* were applied.
- **Sample preparation, if any:** All the samples were rinsed extensively with pure H_2O (Purelab Chorus 1, Elga LabWater, Celle, Germany, 18.2 MΩ cm) and dried with N_2, then used immediately for the AFM imaging.
- **Additional imaging parameters:** in air, 25°C, 1024×1024 pixels, a scan rate of 1 Hz, scan angle of 0° (fast scan direction perpendicular to the cantilever axis) and scan sizes of 30×30, 10×10, 5×5, 1×1 μm^2, respectively.

AUTHOR AND TECHNIQUE INFORMATION FOR THE SURFACE-TOPOGRAPHY CHALLENGE

Submit to: SurfaceTopographyChallenge@gmail.com ***(***Deadline: 29 Feb 2024***)***

- We created this template to standardize the information we get from each group.
- Information submitted will be published *verbatim* in the Supplementary Information section.
- ***Please fill out all yellow-highlighted fields to the best of your ability.***

AUTHORSHIP INFORMATION (*Text only, please. We cannot accept citations in this document.)*

- **Author information (please ONLY include authors that directly contributed)**
  - **Author 1:** Kalliorinne Kalle
    - **ORCID ID:** 0000-0002-4085-8306
    - **Address, Line 1:** Division of Machine Elements, Luleå University of Technology
    - **Address, Line 2:** Luleå University of Technology, Luleå, SE97187, Sweden
  - **Author 2:** Almqvist Andreas
    - **ORCID ID:** 0000-0001-7029-1112
    - **Address, Line 1:** Division of Machine Elements, Luleå University of Technology
    - **Address, Line 2:** Luleå University of Technology, Luleå, SE97187, Sweden
  - *(NOTE: For three or more authors, please copy/paste additional Author lines)*

- **Funding information, if any** *(how it should appear in the acknowledgements)*
  - **Funder:** The Swedish Research Council; **Grant number:** 2019-04293
    - *(NOTE: For more than one funder, please copy/paste additional Funding lines)*

DESCRIPTION OF TECHNIQUES USED FOR DATA COLLECTION

(For reproducibility purposes, the publishing journal requires that we specify all details of each technique used. If you have any questions or concerns, contact SurfaceTopographyChallenge@gmail.com)

- **Number of techniques used:** 1.

TECHNIQUE 1: [NOTE: Please copy/paste this section and repeat for each technique used]

(*Text only, please. We cannot accept citations in this document.)*

- **Common name of technique:** White light interferometer.
- **Type of technique:** white light interferometer chromatic aberration.
  - [atomic force microscope; white light interferometer chromatic aberration; digital 3D optical microscope; confocal laser scanning microscope; holographic measurement; angle-resolved spectroscopy; scanning electron microscope; reconstruction from scanning electron microscope; stylus profilometer/tactile microscope; transmission electron microscope; triboindenter; other (specify)]
- **Instrument manufacturer:** Zygo Corporation, Middlefield, Connecticut.
- **Instrument model:** Zygo NewView 9000.
- **Tip size (if known):** N/A.
- **Expected maximum lateral resolution:** 1.735 um.
- **Data post-processing:** Form removal (Plane).

Sample preparation, if any: Ethanol washing and wiping with KIMTECH Science ”Delicate Task

- Wipes”.

AUTHOR AND TECHNIQUE INFORMATION FOR THE SURFACE-TOPOGRAPHY CHALLENGE

Submit to: SurfaceTopographyChallenge@gmail.com ***(***Deadline: 29 Feb 2024***)***

- We created this template to standardize the information we get from each group.
- Information submitted will be published *verbatim* in the Supplementary Information section.
- ***Please fill out all yellow-highlighted fields to the best of your ability.***

AUTHORSHIP INFORMATION (*Text only, please. We cannot accept citations in this document.)*

- **Author information (please ONLY include authors that directly contributed)**
  - **Author 1:** Amann, Tobias
    - **ORCID ID:** https://orcid.org/0000-0002-3683-0183
    - **Address, Line 1:** Fraunhofer Institute for Mechanics of Materials IWM, Tribology
    - **Address, Line 2:** Woehlerstr. 11 | 79108 Freiburg, Germany

DESCRIPTION OF TECHNIQUES USED FOR DATA COLLECTION

(For reproducibility purposes, the publishing journal requires that we specify all details of each technique used. If you have any questions or concerns, contact SurfaceTopographyChallenge@gmail.com)

- **Number of techniques used:** 2

TECHNIQUE 1: [NOTE: Please copy/paste this section and repeat for each technique used]

(*Text only, please. We cannot accept citations in this document.)*

- **Common name of technique:** Optical surface analysis
- **Type of technique:** digital 3D optical microscope
- **Instrument manufacturer:** Keyence Corporation, Osaka, Japan
- **Instrument model:** Color 3D Laser Microscope, VK-9710K
- **Tip size (if known):** N/A
- **Expected maximum lateral resolution:** 0.13 µm
- **Data post-processing:** No data processing
- **Sample preparation, if any:** The samples were touched only with tweezers. The surface was cleaned before measurement with a paper towel provided with isopropanol.

TECHNIQUE 2 (if applicable): [NOTE: Please copy/paste this section and repeat for each technique used]

(*Text only, please. We cannot accept citations in this document.)*

- **Common name of technique:** Optical surface analysis
- **Type of technique:** confocal laser scanning microscope
- **Instrument manufacturer:** confovis GmbH, Jena, Germany
- **Instrument model:** TOOLinspect 3D surface measuring device
- **Tip size (if known):** N/A
- **Expected maximum lateral resolution:** 0.28 µm
- **Data post-processing:** No data processing
- **Sample preparation, if any:** The samples were touched only with tweezers. The surface was cleaned before measurement with a paper towel provided with isopropanol.

AUTHOR AND TECHNIQUE INFORMATION FOR THE SURFACE-TOPOGRAPHY CHALLENGE

Submit to: SurfaceTopographyChallenge@gmail.com ***(***Deadline: 29 Feb 2024***)***

- We created this template to standardize the information we get from each group.
- Information submitted will be published *verbatim* in the Supplementary Information section.
- ***Please fill out all yellow-highlighted fields to the best of your ability.***

AUTHORSHIP INFORMATION (*Text only, please. We cannot accept citations in this document.)*

- **Author information (please ONLY include authors that directly contributed)**
  - **Author 1:** Aldave, Diego Alonso
    - **ORCID ID:** 0000-0002-6967-157X
    - **Address, Line 1:** Departamento de Física de la Materia Condensada and Condensed Matter Physics Center (IFIMAC), Universidad Autónoma de Madrid
    - **Address, Line 2:** Facultad de Ciencias, C/ Francisco Tomás y Valiente 7, Madrid, 28049, Spain
  - **Author 2:** Ares, Pablo
    - **ORCID ID:** 0000-0001-5905-540X
    - **Address, Line 1:** Departamento de Física de la Materia Condensada and Condensed Matter Physics Center (IFIMAC), Universidad Autónoma de Madrid
    - **Address, Line 2:** Facultad de Ciencias, C/ Francisco Tomás y Valiente 7, Madrid, 28049, Spain
  - **Author 3:** Gómez-Herrero, Julio
    - **ORCID ID:** 0000-0001-8583-8061
    - **Address, Line 1:** Departamento de Física de la Materia Condensada and Condensed Matter Physics Center (IFIMAC), Universidad Autónoma de Madrid
    - **Address, Line 2:** Facultad de Ciencias, C/ Francisco Tomás y Valiente 7, Madrid, 28049, Spain
  - **Author 4:** Vilhena, J. G.
    - **ORCID ID:** 0000-0001-8338-9119
    - **Address, Line 1:** Institute of Material Science of Madrid (ICMM-CSIC)
    - **Address, Line 2:** C/ Sor Juana Inés de la Cruz 3, Madrid, 28049, Spain

- **Funding information, if any** *(how it should appear in the acknowledgements)*
  - **Funder:** Comunidad de Madrid & Universidad Autónoma de Madrid; **Grant number:** SI3-PJI-2021-00479.
  - **Funder:** Comunidad de Madrid; **Grant number:** 2020-T1/ND-20306 (Talento Program).
  - **Funder:** MCIN/AEI/10.13039/501100011033; **Grant numbers:** TED2021-132219A-I00, PID2022-142331NB-I00, PID2022-138908NB-C32, PID2020-113722RJ-I00, Ramón y Cajal fellowship RYC2020-030302-I, "María de Maeztu" Programme for Units of Excellence in R&D CEX2018-000805-M

DESCRIPTION OF TECHNIQUES USED FOR DATA COLLECTION

(For reproducibility purposes, the publishing journal requires that we specify all details of each technique used. If you have any questions or concerns, contact SurfaceTopographyChallenge@gmail.com)

- **Number of techniques used:** 1

TECHNIQUE 1: [NOTE: Please copy/paste this section and repeat for each technique used]

(*Text only, please. We cannot accept citations in this document.)*

- **Common name of technique:** AFM
- **Type of technique:** Atomic Force Microscope, Contact mode
- **Instrument manufacturer:** Nanotec Electronica S.L., Madrid, Spain
- **Instrument model:** Cervantes AFM
- **Tip size (if known):** 10 nm radius
- **Expected maximum lateral resolution:** 10 nm
- **Data post-processing:** We employed tilt correction, and 2nd degree polynomial background removal for scans 7.5 and 3.8 µm of the rough samples.
- **Sample preparation, if any:** All samples were cleaned with a gentle stream of N_2 gas prior to AFM imaging.

AUTHOR AND TECHNIQUE INFORMATION FOR THE SURFACE-TOPOGRAPHY CHALLENGE

Submit to: SurfaceTopographyChallenge@gmail.com ***(***Deadline: 29 Feb 2024***)***

- We created this template to standardize the information we get from each group.
- Information submitted will be published *verbatim* in the Supplementary Information section.
- ***Please fill out all yellow-highlighted fields to the best of your ability.***

AUTHORSHIP INFORMATION

- **Author information (please ONLY include authors that directly contributed)**
  - **Author 1:** Cihan, Ebru
    - **ORCID ID:** 0000-0002-1747-3838
    - **Address, Line 1:** Institute for Materials Science and Max Bergmann Center for Biomaterials, TU Dresden
    - **Address, Line 2:** Budapester Str 27, Room 107, 01069 Dresden, Germany

DESCRIPTION OF TECHNIQUES USED FOR DATA COLLECTION

- **Number of techniques used:** 2

TECHNIQUE 1: [NOTE: Please copy/paste this section and repeat for each technique used]

- **Common name of technique:** AFM
- **Type of technique:** Atomic Force Microscope, tapping mode
- **Instrument manufacturer:** Digital Instruments (now Bruker), Santa Barbara, California, USA
- **Instrument model:** Bioscope
- **Tip size (if known):** r < 30 nm
- **Expected maximum lateral resolution:** 30 nm
- **Data post-processing:** Line correction in instrument software (Nanoscope) during image acquisition
- **Sample preparation, if any:** Cleaning dust particles of the surface using dry nitrogen

---

TECHNIQUE 2 (if applicable): [NOTE: Please copy/paste this section and repeat for each technique used]

- **Common name of technique:** AFM
- **Type of technique:** Atomic Force Microscope, contact mode
- **Instrument manufacturer:** Digital Instruments (now Bruker), Santa Barbara, California, USA
- **Instrument model:** Bioscope
- **Tip size (if known):** r < 25 nm
- **Expected maximum lateral resolution:** 25 nm
- **Data post-processing:** Line correction in instrument software (Nanoscope) during image acquisition
- **Sample preparation, if any:** Cleaning dust particles of the surface using dry nitrogen

AUTHOR AND TECHNIQUE INFORMATION FOR THE SURFACE-TOPOGRAPHY CHALLENGE

Submit to: SurfaceTopographyChallenge@gmail.com ***(***Deadline: 29 Feb 2024***)***

- We created this template to standardize the information we get from each group.
- Information submitted will be published *verbatim* in the Supplementary Information section.
- ***Please fill out all yellow-highlighted fields to the best of your ability.***

AUTHORSHIP INFORMATION (*Text only, please. We cannot accept citations in this document.)*

- **Author information (please ONLY include authors that directly contributed)**

  - **Author 1:** Descartes, Sylvie
    - **ORCID ID:** 0000-0003-0611-1820
    - **Address, Line 1:** INSA Lyon, CNRS, LaMCoS, UMR5259
    - **Address, Line 2:** Bldg S. Germain, 27bis Ave. Jean Capelle, Villeurbanne, 69621, France
  - **Author 2:** Albertini, David
    - **ORCID ID:** --
    - **Address, Line 1:** CNRS, Université Claude Bernard Lyon 1, INSA Lyon, INL, UMR 5270
    - **Address, Line 2:** Bldg I. Joliot Curie, 1 rue Enrico Fermi, Villeurbanne, 69622, France

- **Funding information, if any** *(how it should appear in the acknowledgements)*
  - **Funder:** LABEX iMUST ; **Grant number:** ANR-10-LABX-0064

  Please note that the sentence should be: "LABEX iMUST (ANR-10-LABX-0064) of Université de Lyon, within the program "Investissements d'Avenir" (ANR-11-IDEX-0007) operated by the French National Research Agency (ANR)."

DESCRIPTION OF TECHNIQUES USED FOR DATA COLLECTION

(For reproducibility purposes, the publishing journal requires that we specify all details of each technique used. If you have any questions or concerns, contact SurfaceTopographyChallenge@gmail.com)

- **Number of techniques used: 3**

TECHNIQUE 1: [NOTE: Please copy/paste this section and repeat for each technique used]

(*Text only, please. We cannot accept citations in this document.)*

- **Common name of technique:** SEM
- **Type of technique:_** Scanning Electron Microscopy
- **Instrument manufacturer:** Thermo Fisher Scientific
- **Instrument model:** QUANTA 600
- **Tip size (if known):** N/A
- **Expected maximum lateral resolution:** 3 nm at 30kV (SE)
- **Data post-processing:** *Note: SEM was used for reference only, not for the quantification of topography.*
- **Sample preparation, if any:** samples were rinsed with ethanol and dried with clean compressed air, before first imaging

---

TECHNIQUE 2: [NOTE: Please copy/paste this section and repeat for each technique used]

(*Text only, please. We cannot accept citations in this document.)*

- **Common name of technique:** Optical/Laser profiler
- **Type of technique:_**white light interferometer
- **Instrument manufacturer:** Zygo Corporation, Middlefield, USA
- **Instrument model:** ZeGage Pro
- **Tip size (if known):** N/A
- **Expected maximum lateral resolution:** 0.52 µm (50X objective) and 0.95 µm (10X objective)
- **Data post-processing:** Tilt correction (method of least squares) and form removal (polynomial of degree 5) for all scans and all samples
- **Sample preparation, if any:** samples were rinsed with ethanol and dried with clean compressed air, before first imaging

AUTHOR AND TECHNIQUE INFORMATION FOR THE SURFACE-TOPOGRAPHY CHALLENGE

Submit to: SurfaceTopographyChallenge@gmail.com ***(***Deadline: 29 Feb 2024***)***

TECHNIQUE 3 : [NOTE: Please copy/paste this section and repeat for each technique used]

(*Text only, please. We cannot accept citations in this document.)*

- **Common name of technique:** AFM
- **Type of technique:** atomic force microscope, tapping mode
- **Instrument manufacturer:** Bruker
- **Instrument model:** Dimension Icon
- **Tip size (if known):** < 7 nm
- **Expected maximum lateral resolution:** < 7 nm
- **Data post-processing:** Tilt correction (method of least squares) and form removal (polynomial of degree 5) for all scans
- **Sample preparation, if any:** -- (*NOTE: analyses were done just after white light interferometer*)

AUTHOR AND TECHNIQUE INFORMATION FOR THE SURFACE-TOPOGRAPHY CHALLENGE
Submit to: SurfaceTopographyChallenge@gmail.com ***(***Deadline: 29 Feb 2024***)***

- We created this template to standardize the information we get from each group.
- Information submitted will be published *verbatim* in the Supplementary Information section.
- ***Please fill out all yellow-highlighted fields to the best of your ability.***

AUTHORSHIP INFORMATION (*Text only, please. We cannot accept citations in this document.)*

- **Author information (please ONLY include authors that directly contributed)**
  - **Author 1:** Stefan Kinzelberger
    - **ORCID ID:** nan
    - **Address, Line 1:** Mikrotribologie Centrum µTC, Fraunhofer-Institute for Mechanics of Materials IWM, Rintheimer Querallee 2b, 76131 Karlsruhe, Germany
    - **Address, Line 2:**
  - **Author 2:** Martin Dienwiebel
    - **ORCID ID:** 0000-0001-7682-0441
    - **Address, Line 1: :** Mikrotribologie Centrum µTC, Fraunhofer-Institute for Mechanics of Materials IWM, Rintheimer Querallee 2b, 76131 Karlsruhe, Germany
    - **Address, Line 2:**Karlsruhe Institute of Technology, Institute for Applied Materials IAM, Straße am Forum 7, 76131 Karlsruhe, Germany

- **Funding information, if any** *(how it should appear in the acknowledgements)*
  - **Funder:** Federal Ministry for Economic Affairs and Climate Action; **Grant number:** 03EN4006A

DESCRIPTION OF TECHNIQUES USED FOR DATA COLLECTION
(For reproducibility purposes, the publishing journal requires that we specify all details of each technique used. If you have any questions or concerns, contact SurfaceTopographyChallenge@gmail.com)

- **Number of techniques used:** 2

TECHNIQUE 1: [NOTE: Please copy/paste this section and repeat for each technique used]
(*Text only, please. We cannot accept citations in this document.)*

- **Common name of technique:** Digital Holographic Microscopy DHM
- **Type of technique:** holographic measurement
  - [atomic force microscope; white light interferometer chromatic aberration; digital 3D optical microscope; confocal laser scanning microscope; holographic measurement; angle-resolved spectroscopy; scanning electron microscope; reconstruction from scanning electron microscope; stylus profilometer/tactile microscope; transmission electron microscope; triboindenter; other (specify)]
- **Instrument manufacturer:** Lyncée Tec SA, Lausanne, Switzerland
- **Instrument model:** Custom made DHM R2100, 20x objective lens (Leica N Plan EPI 20/0.4)
- **Tip size (if known):** N/A
- **Expected maximum lateral resolution:** 800 nm
- **Data post-processing:** The measurement data was converted from native Lyncée Tec Koala software was converted to an XYZ file using the Bruker Vision64 software (.sdf file) and from there to XYZ data using Gwyddion The tilt was removed online with the analyzing tools offered by "contact.engineering". No further post-processing was applied.
- **Sample preparation, if any:** none

TECHNIQUE 2 (if applicable): [NOTE: Please copy/paste this section and repeat for each technique used]
(*Text only, please. We cannot accept citations in this document.)*

- **Common name of technique:** White Light Interferometry WLI
- **Type of technique:** white light interferometer
  - [atomic force microscope; white light interferometer chromatic aberration; digital 3D optical microscope; confocal laser scanning microscope; holographic measurement; angle-resolved spectroscopy; scanning electron microscope; reconstruction from scanning electron microscope; stylus profilometer/tactile microscope; transmission electron microscope; triboindenter; other (specify)]
- **Instrument manufacturer:** Bruker
- **Instrument model:** Contour GT, 50x lens
- **Tip size (if known):** n/a

AUTHOR AND TECHNIQUE INFORMATION FOR THE SURFACE-TOPOGRAPHY CHALLENGE

Submit to: SurfaceTopographyChallenge@gmail.com ***(***Deadline: 29 Feb 2024***)***

- **Expected maximum lateral resolution:** 1 µm
- **Data post-processing:** The measurement was saved as an surface-data-frame via Vision64 and changed into a .XYZ-file with Gwyddion. The tilt was removed online with the analyzing tools offered by "contact.engineering". No further post-processing was applied
- **Sample preparation, if any:** none

AUTHOR AND TECHNIQUE INFORMATION FOR THE SURFACE-TOPOGRAPHY CHALLENGE

Submit to: SurfaceTopographyChallenge@gmail.com ***(***Deadline: 29 Feb 2024***)***

- We created this template to standardize the information we get from each group.
- Information submitted will be published *verbatim* in the Supplementary Information section.
- ***Please fill out all yellow-highlighted fields to the best of your ability.***

AUTHORSHIP INFORMATION (*Text only, please. We cannot accept citations in this document.)*

- **Author information (please ONLY include authors that directly contributed)**
  - **Author 1:** Daniele Dini
    - **ORCID ID:** 0000-0002-5518-499X
    - **Address, Line 1:** Department of Mechanical Engineering
    - **Address, Line 2:** Exhibition Road, Imperial College London, London, UK SW7 2AZ
  - **Author 2:** Jie Zhang
    - **ORCID ID:** 0000-0001-7572-7077
    - **Address, Line 1:** Department of Mechanical Engineering
    - **Address, Line 2:** Exhibition Road, Imperial College London, London, UK SW7 2AZ
  - *(NOTE: For three or more authors, please copy/paste additional Author lines)*

- **Funding information, if any** *(how it should appear in the acknowledgements)*
  - **Funder:** UK Engineering and Physical Sciences Research Council (EPSRC); **Grant number** EP/V038044/1
  - **Funder:** UK Royal Academy of Engineering, Scheme: Research Chair (Prof. Dini); **Grant number:** RCSRF2122-14-143
    - *(NOTE: For more than one funder, please copy/paste additional Funding lines)*

DESCRIPTION OF TECHNIQUES USED FOR DATA COLLECTION

(For reproducibility purposes, the publishing journal requires that we specify all details of each technique used. If you have any questions or concerns, contact SurfaceTopographyChallenge@gmail.com)

- **Number of techniques used:** 3

TECHNIQUE 1: [NOTE: Please copy/paste this section and repeat for each technique used]

(*Text only, please. We cannot accept citations in this document.)*

- **Common name of technique:** confocal
- **Type of technique:** confocal laser scanning microscope
- **Instrument manufacturer:** Olympus, Tokyo, Japan
- **Instrument model:** LEXT OLS5100 3D Measuring Laser Microscope
- **Tip size (if known):** N/A
- **Expected maximum lateral resolution:** 0.12 µm
- **Data post-processing:** Tilt correction
- **Sample preparation, if any:** Rinsed with Toluene and isopropanol then dried with compressed air

---

TECHNIQUE 2 (if applicable): [NOTE: Please copy/paste this section and repeat for each technique used]

(*Text only, please. We cannot accept citations in this document.)*

- **Common name of technique:** WLI
- **Type of technique:** white light interferometer
- **Instrument manufacturer:** Bruker, MA, USA
- **Instrument model:** Bruker ContourGT
- **Tip size (if known):** N/A
- **Expected maximum lateral resolution:** 0.13 µm
- **Data post-processing:** Tilt correction
- **Sample preparation, if any:** Rinsed with Toluene and isopropanol then dried with compressed air

---

TECHNIQUE 3 (if applicable): [NOTE: Please copy/paste this section and repeat for each technique used]

(*Text only, please. We cannot accept citations in this document.)*

- **Common name of technique:** AFM
- **Type of technique:** atomic force microscope, contact mode
- **Instrument manufacturer:** WiTec, Ulm, Germany
- **Instrument model:** WiTec alpha300RA – Raman-AFM Microscope

AUTHOR AND TECHNIQUE INFORMATION FOR THE SURFACE-TOPOGRAPHY CHALLENGE

Submit to: SurfaceTopographyChallenge@gmail.com ***(***Deadline: 29 Feb 2024***)***

- **Tip size (if known):** <10 nm
- **Expected maximum lateral resolution:** ~0.2 µm
- **Data post-processing:** Tilt correction
- **Sample preparation, if any:** Rinsed with Toluene and isopropanol then dried with compressed air

AUTHOR AND TECHNIQUE INFORMATION FOR THE SURFACE-TOPOGRAPHY CHALLENGE

Submit to: SurfaceTopographyChallenge@gmail.com ***(***Deadline: 29 Feb 2024***)***

- We created this template to standardize the information we get from each group.
- Information submitted will be published *verbatim* in the Supplementary Information section.
- ***Please fill out all yellow-highlighted fields to the best of your ability.***

AUTHORSHIP INFORMATION (*Text only, please. We cannot accept citations in this document.)*

- **Author information (please ONLY include authors that directly contributed)**
  - **Author 1:** Dunn, Alison C.
    - **ORCID ID:** 0000-0002-4841-1293
    - **Address, Line 1:** Department of Mechanical and Aerospace Engineering, University of Florida
    - **Address, Line 2:** 1064 Center Drive, Building NEB Room 181, Gainesville, FL 32611, USA
  - **Author 2:** Rahman, Md Habibur
    - **ORCID ID:** 0000-0003-1936-1917
    - **Address, Line 1:** Department of Mechanical Science and Engineering, University of Illinois Urbana-Champaign
    - **Address, Line 2:** 1206 W Green St, Urbana, IL 61801, USA
  - **Author 3:** Ali, Nabila
    - **ORCID ID:** 0009-0009-9021-2050
    - **Address, Line 1:** Department of Mechanical Science and Engineering, University of Illinois Urbana-Champaign
    - **Address, Line 2:** 1206 W Green St, Urbana, IL 61801, USA
  - *(NOTE: For three or more authors, please copy/paste additional Author lines)*

- **Funding information, if any** *(how it should appear in the acknowledgements)*
  - **Funder:** National Science Foundation (USA); **Grant number:** 2219787
    - *(NOTE: For more than one funder, please copy/paste additional Funding lines)*

DESCRIPTION OF TECHNIQUES USED FOR DATA COLLECTION

(For reproducibility purposes, the publishing journal requires that we specify all details of each technique used. If you have any questions or concerns, contact SurfaceTopographyChallenge@gmail.com)

- **Number of techniques used:** 1

TECHNIQUE 1: [NOTE: Please copy/paste this section and repeat for each technique used]

(*Text only, please. We cannot accept citations in this document.)*

- **Common name of technique:** Optical
- **Type of technique:** Confocal laser scanning microscope
- **Instrument manufacturer:** Keyence, 500 Park Boulevard, Suite 200, Itasca, IL 60143, U.S.A.
- **Instrument model:** Keyence VK-X1000 Profile-analyzing Laser Microscope
- **Tip size (if known):** n/a
- **Expected maximum lateral resolution:** 2.69 µm (5X), 1.369 µm (10X), 0.684 µm (20X), 0.276 µm (50X)
- **Data post-processing:** Tilt correction and surface shape correction with waveform removal method [cutoff wavelength 800 µm (5X), 250 µm (10X), 200 µm (20X), 80µm (50X)]
- **Sample preparation, if any:** Samples were cleaned with Ethanol before placing under the microscope.

AUTHOR AND TECHNIQUE INFORMATION FOR THE SURFACE-TOPOGRAPHY CHALLENGE

Submit to: SurfaceTopographyChallenge@gmail.com ***(***Deadline: 29 Feb 2024***)***

- We created this template to standardize the information we get from each group.
- Information submitted will be published *verbatim* in the Supplementary Information section.
- ***Please fill out all yellow-highlighted fields to the best of your ability.***

AUTHORSHIP INFORMATION (*Text only, please. We cannot accept citations in this document.)*

- **Author information (please ONLY include authors that directly contributed)**
  - **Author 1:** Soni, Jitendra
    - **ORCID ID:** 0000-0002-8427-3860
    - **Address, Line 1:** Department of Materials Science and Engineering, Indian Institute of Technology Delhi
    - **Address, Line 2:** Hauz Khas, New Delhi, 110016, India
  - **Author 2:** Gosvami, Nitya Nand
    - **ORCID ID:** 0000-0003-4082-9887
    - **Address, Line 1:** Department of Materials Science and Engineering, Indian Institute of Technology Delhi
    - **Address, Line 2:** Hauz Khas, New Delhi, 110016, India
  - *(NOTE: For three or more authors, please copy/paste additional Author lines)*

- **Funding information, if any** *(how it should appear in the acknowledgements)*
  - **Funder:** Indian Institute of Technology Delhi **Grant number:** MI02564G
  - **Funder:** SERB **Grant number:** CRG/2020/002062

DESCRIPTION OF TECHNIQUES USED FOR DATA COLLECTION

(For reproducibility purposes, the publishing journal requires that we specify all details of each technique used. If you have any questions or concerns, contact SurfaceTopographyChallenge@gmail.com)

- **Number of techniques used:** 2

TECHNIQUE 1: [NOTE: Please copy/paste this section and repeat for each technique used]

(*Text only, please. We cannot accept citations in this document.)*

- **Common name of technique:** AFM
- **Type of technique:** atomic force microscope, contact mode
- **Instrument manufacturer:** Nanosurf, Switzerland
- **Instrument model:** DriveAFM
- **Tip size (if known):** ~40 nm
- **Expected maximum lateral resolution:** 20 nm
- **Data post-processing:** No
- **Sample preparation, if any:** Samples were cleaned with ethanol using ultrasonication.

TECHNIQUE 2 (if applicable): [NOTE: Please copy/paste this section and repeat for each technique used]

(*Text only, please. We cannot accept citations in this document.)*

- **Common name of technique:** AFM
- **Type of technique:** atomic force microscope, tapping mode
- **Instrument manufacturer:** Nanosurf, Switzerland
- **Instrument model:** DriveAFM
- **Tip size (if known):** ~40 nm
- **Expected maximum lateral resolution:** 20 nm
- **Data post-processing:** No
- **Sample preparation, if any:** Samples were cleaned with ethanol using ultrasonication.

AUTHOR AND TECHNIQUE INFORMATION FOR THE SURFACE-TOPOGRAPHY CHALLENGE

Submit to: SurfaceTopographyChallenge@gmail.com ***(***Deadline: 29 Feb 2024***)***

- We created this template to standardize the information we get from each group.
- Information submitted will be published *verbatim* in the Supplementary Information section.
- ***Please fill out all yellow-highlighted fields to the best of your ability.***

AUTHORSHIP INFORMATION (*Text only, please. We cannot accept citations in this document.)*

- **Author information (please ONLY include authors that directly contributed)**
    - **Author 1:** Greiner, Christian
        - **ORCID ID:** 0000-0001-8079-336X
        - **Address, Line 1:** Institute for Applied Materials (IAM), Karlsruhe Institute of Technology (KIT)
        - **Address, Line 2:** Kaiserstr.12, 76131, Karlsruhe, Germany
    - **Author 2:** Li, Yulong
        - **ORCID ID:** 0000-0001-9899-483X
        - **Address, Line 1:** Institute for Applied Materials (IAM), Karlsruhe Institute of Technology (KIT)
        - **Address, Line 2:** Kaiserstr.12, 76131, Karlsruhe, Germany
    - *(NOTE: For three or more authors, please copy/paste additional Author lines)*

- **Funding information, if any** *(how it should appear in the acknowledgements)*
    - **Funder:** European Research Council; **Grant number:** 771237
        - *(NOTE: For more than one funder, please copy/paste additional Funding lines)*

DESCRIPTION OF TECHNIQUES USED FOR DATA COLLECTION

(For reproducibility purposes, the publishing journal requires that we specify all details of each technique used. If you have any questions or concerns, contact SurfaceTopographyChallenge@gmail.com)

- **Number of techniques used:** 4

TECHNIQUE 1: [NOTE: Please copy/paste this section and repeat for each technique used]

(*Text only, please. We cannot accept citations in this document.)*

- **Common name of technique:** Stylus
- **Type of technique:** Stylus profilometer
- **Instrument manufacturer:** Hommel-Etamic, Villingen-Schwenningen, Germany
- **Instrument model:** T8000 R120-400
- **Tip size (if known):** Diamond 5 µm 90°
- **Expected maximum lateral resolution:** 0.1 µm
- **Data post-processing:** No data processing
- **Sample preparation, if any:** After undergoing two rounds of ultrasonic cleaning in isopropanol, each for a duration of 10 minutes, the samples were subsequently tested using all four methods within a single day.

TECHNIQUE 2 (if applicable): [NOTE: Please copy/paste this section and repeat for each technique used]

(*Text only, please. We cannot accept citations in this document.)*

- **Common name of technique:** Confocal Microscope
- **Type of technique:** Confocal laser scanning microscope
- **Instrument manufacturer:** Sensofar, Barcelona, Spain
- **Instrument model:** Plµ Neox
- **Tip size (if known):** Non-contact instrument
- **Expected maximum lateral resolution:**
    - 0.46 µm for 10x magnification objective
    - 0.31 µm for 20x magnification objective
    - 0.17 µm for 50x magnification objective
    - 0.15 µm for 150x magnification objective
- **Data post-processing:** No data processing
- **Sample preparation, if any:** After undergoing two rounds of ultrasonic cleaning in isopropanol, each for a duration of 10 minutes, the samples were subsequently tested using all four methods within a single day.

TECHNIQUE 3 (if applicable): [NOTE: Please copy/paste this section and repeat for each technique used]

AUTHOR AND TECHNIQUE INFORMATION FOR THE SURFACE-TOPOGRAPHY CHALLENGE

Submit to: SurfaceTopographyChallenge@gmail.com ***(***Deadline: 29 Feb 2024***)***

(*Text only, please. We cannot accept citations in this document.)*

- **Common name of technique:** WLI
- **Type of technique:** White light interferometer
- **Instrument manufacturer:** Sensofar, Barcelona, Spain
- **Instrument model:** Plµ Neox
- **Tip size (if known):** Non-contact instrument
- **Expected maximum lateral resolution:**
    - 0.46 µm for 10x magnification objective
    - 0.25 µm for 50x magnification objective
- **Data post-processing:** No data processing
- **Sample preparation, if any:** After undergoing two rounds of ultrasonic cleaning in isopropanol, each for a duration of 10 minutes, the samples were subsequently tested using all four methods within a single day.

TECHNIQUE 4 (if applicable): [NOTE: Please copy/paste this section and repeat for each technique used]

(*Text only, please. We cannot accept citations in this document.)*

- **Common name of technique:** Chromatic aberration
- **Type of technique:** Chromatic aberration
    - [atomic force microscope; white light interferometer chromatic aberration; digital 3D optical microscope; confocal laser scanning microscope; holographic measurement; angle-resolved spectroscopy; scanning electron microscope; reconstruction from scanning electron microscope; stylus profilometer/tactile microscope; transmission electron microscope; triboindenter; other (specify)]
- **Instrument manufacturer:** Fries Research and Technology GmbH (now owned by FormFactor), Bergisch Gladbach, Germany
- **Instrument model:** MicroProf
- **Tip size (if known):** Non-contact instrument
- **Expected maximum lateral resolution:** 1 µm
- **Data post-processing:** No data processing
- **Sample preparation, if any:** After undergoing two rounds of ultrasonic cleaning in isopropanol, each for a duration of 10 minutes, the samples were subsequently tested using all four methods within a single day.

AUTHOR AND TECHNIQUE INFORMATION FOR THE SURFACE-TOPOGRAPHY CHALLENGE

Submit to: SurfaceTopographyChallenge@gmail.com ***(***Deadline: 29 Feb 2024***)***

- We created this template to standardize the information we get from each group.
- Information submitted will be published *verbatim* in the Supplementary Information section.
- ***Please fill out all yellow-highlighted fields to the best of your ability.***

AUTHORSHIP INFORMATION (*Text only, please. We cannot accept citations in this document.*)

- **Author information**
  - **Author 1:** Hasan, Mushfiq
    - **ORCID ID:** 0009-0008-7511-4906
    - **Address, Line 1:** Division of Machine Elements, Department of Engineering Science and Mathematics,
    - **Address, Line 2:** Luleå University of Technology, SE-97187 Luleå, Sweden
  - **Author 2:** Björling, Marcus
    - **ORCID ID:** 0000-0002-4271-0380
    - **Address, Line 1:** Division of Machine Elements, Department of Engineering Science and Mathematics
    - **Address, Line 2:** Luleå University of Technology, SE-97187 Luleå, Sweden
  - **Author 3:** Larsson, Roland
    - **ORCID ID:** 0000-0001-9110-2819
    - **Address, Line 1:** Division of Machine Elements, Department of Engineering Science and Mathematics,
    - **Address, Line 2:** Luleå University of Technology, SE-97187 Luleå, Sweden

- **Funding information, if any** *(how it should appear in the acknowledgements)*
  - **Funder:** Swedish Energy Agency (FFI); **Grant number:** 2020-024802
    - *(NOTE: For more than one funder, please copy/paste additional Funding lines)*

DESCRIPTION OF TECHNIQUES USED FOR DATA COLLECTION

(For reproducibility purposes, the publishing journal requires that we specify all details of each technique used. If you have any questions or concerns, contact SurfaceTopographyChallenge@gmail.com)

- **Number of techniques used:** 1.

TECHNIQUE 1: [NOTE: Please copy/paste this section and repeat for each technique used]

(*Text only, please. We cannot accept citations in this document.*)

- **Common name of technique:** Coherence Scanning Interferometry
- **Type of technique**: White Light Interferometer
- **Instrument manufacturer:** Zygo Corporation, USA
- **Instrument model:** ZYGO, Newview 9000
- **Tip size (if known):** N/A
- **Expected maximum lateral resolution:** As per manual 0.34 µm for 100X objective. (measurements were taken using 2.75X and 10X objectives)
- **Data post-processing:** MX software provided with the Zygo instrument (Form removal and tilt correction)
- **Sample preparation, if any:** Cleaned with ethanol wipes before measurements

AUTHOR AND TECHNIQUE INFORMATION FOR THE SURFACE-TOPOGRAPHY CHALLENGE

Submit to: SurfaceTopographyChallenge@gmail.com ***(***Deadline: 29 Feb 2024***)***

- We created this template to standardize the information we get from each group.
- Information submitted will be published *verbatim* in the Supplementary Information section.
- ***Please fill out all yellow-highlighted fields to the best of your ability.***

AUTHORSHIP INFORMATION (*Text only, please. We cannot accept citations in this document.)*

- **Author information (please ONLY include authors that directly contributed)**
  - **Author 1:** Hoppe, Svenja
    - **ORCID ID:** 0009-0003-8264-0529
    - **Address, Line 1:** Currenta GmbH & Co. OHG, Surface and Solid-State Analytics
    - **Address, Line 2:** CHEMPARK Leverkusen, 51368 Leverkusen, Germany
  - **Author 2:** Dr. Gabriel, Dina
    - **ORCID ID:** N/A
    - **Address, Line 1:** Currenta GmbH & Co. OHG, Surface and Solid-State Analytics
    - **Address, Line 2:** CHEMPARK Leverkusen, 51368 Leverkusen, Germany
  - *(NOTE: For three or more authors, please copy/paste additional Author lines)*

- **Funding information, if any** *(how it should appear in the acknowledgements)*
  - **Funder:** N/A ; **Grant number:** N/A
    - *(NOTE: For more than one funder, please copy/paste additional Funding lines)*

DESCRIPTION OF TECHNIQUES USED FOR DATA COLLECTION

(For reproducibility purposes, the publishing journal requires that we specify all details of each technique used. If you have any questions or concerns, contact SurfaceTopographyChallenge@gmail.com)

- **Number of techniques used:** 1

TECHNIQUE 1: [NOTE: Please copy/paste this section and repeat for each technique used]

(*Text only, please. We cannot accept citations in this document.)*

- **Common name of technique:** atomic force microscopy
- **Type of technique:** atomic force microscope
  - [atomic force microscope; white light interferometer chromatic aberration; digital 3D optical microscope; confocal laser scanning microscope; holographic measurement; angle-resolved spectroscopy; scanning electron microscope; reconstruction from scanning electron microscope; stylus profilometer/tactile microscope; transmission electron microscope; triboindenter; other (specify)]
- **Instrument manufacturer:** Asylum Research, Oxford Instruments, Santa Barbara, CA
- **Instrument model:** Jupiter XR
- **Tip size (if known):** 8nm radius
- **Expected maximum lateral resolution:** 97,8nm/Pixel (by 50µm scan size and 512 points & lines)
- **Data post-processing:** Data were calculated with the Asylum Research software. On the images from the smoother probes, I proceed a flatting, Histogram order, as well surface contamination was filtered out. Only empty lines were deleted from the images of the rough samples.
- **Sample preparation, if any:** none extra sample preparation

AUTHOR AND TECHNIQUE INFORMATION FOR THE SURFACE-TOPOGRAPHY CHALLENGE

Submit to: SurfaceTopographyChallenge@gmail.com ***(***Deadline: 29 Feb 2024***)***

- We created this template to standardize the information we get from each group.
- Information submitted will be published *verbatim* in the Supplementary Information section.
- ***Please fill out all yellow-highlighted fields to the best of your ability.***

AUTHORSHIP INFORMATION (*Text only, please. We cannot accept citations in this document.)*

- **Author information (please ONLY include authors that directly contributed)**

  - **Author 1:** Huang, De
    - **ORCID ID:** 0000-0002-3085-2031
    - **Address, Line 1:** SAINT-GOBAIN Omniseal Solutions
    - **Address, Line 2:** Heiveldekens 22, 2550 Kontich, Belgium

  - *(NOTE: For three or more authors, please copy/paste additional Author lines)*
- **Funding information, if any** *(how it should appear in the acknowledgements)*

DESCRIPTION OF TECHNIQUES USED FOR DATA COLLECTION

(For reproducibility purposes, the publishing journal requires that we specify all details of each technique used. If you have any questions or concerns, contact SurfaceTopographyChallenge@gmail.com)

- **Number of techniques used:** 2

TECHNIQUE 1: [NOTE: Please copy/paste this section and repeat for each technique used]

(*Text only, please. We cannot accept citations in this document.)*

- **Common name of technique:** Stylus
- **Type of technique:** Stylus Profilometer
- **Instrument manufacturer:** Mitutoyo Europe GmbH, Borsigstraße 8-10 D-41469 Neuss,Germany
- **Instrument model:** Mitutoyo SJ-500P
- **Tip size (if known):** 2 µm radius
- **Expected maximum lateral resolution:** 0.05 µm
- **Data post-processing:** Inclination compensation
- **Sample preparation, if any:** Sample cleaning with ethanol when significant contamination is observed, otherwise, sample is measured as is.

TECHNIQUE 2 (if applicable): [NOTE: Please copy/paste this section and repeat for each technique used]

(*Text only, please. We cannot accept citations in this document.)*

- **Common name of technique:** Microscope
- **Type of technique:** Confocal laser scanning microscope
- **Instrument manufacturer:** OLYMPUS
- **Instrument model:** LEXT OLS4100
- **Tip size (if known):** N/A
- **Expected maximum lateral resolution:** 0.12 µm
- **Data post-processing:** Inclination compensation
- **Sample preparation, if any:** Sample cleaning with ethanol when significant contamination is observed, otherwise, sample is measured as is.

AUTHOR AND TECHNIQUE INFORMATION FOR THE SURFACE-TOPOGRAPHY CHALLENGE

Submit to: SurfaceTopographyChallenge@gmail.com ***(***Deadline: 29 Feb 2024***)***

- We created this template to standardize the information we get from each group.
- Information submitted will be published *verbatim* in the Supplementary Information section.
- ***Please fill out all yellow-highlighted fields to the best of your ability.***

AUTHORSHIP INFORMATION (*Text only, please. We cannot accept citations in this document.)*

- **Author information (please ONLY include authors that directly contributed)**
  - **Author 1:** Baugh, Loren
    - **ORCID ID:** 0009-0009-7620-9218
    - **Address, Line 1:** Department of Mechanical Engineering, Auburn University
    - **Address, Line 2:** 354 War Eagle Way, Wiggins Hall, Auburn, AL 36849
  - **Author 2:** Mahmood, Samsul
    - **ORCID ID:** 0000-0003-3595-3891
    - **Address, Line 1:** Department of Mechanical Engineering, Auburn University
    - **Address, Line 2:** 311 W Magnolia Ave, Gavin Research Laboratory, Auburn, AL, 36849
  - **Author 3:** Schulze, Kyle D.
    - **ORCID ID:** 0000-0001-8433-0581
    - **Address, Line 1:** Department of Mechanical Engineering, Auburn University
    - **Address, Line 2:** 354 War Eagle Way, Wiggins Hall, Auburn, AL 36849
  - **Author 4:** Jackson, Robert L.
    - **ORCID ID:** 0000-0002-3316-9510
    - **Address, Line 1:** Department of Mechanical Engineering, Auburn University
    - **Address, Line 2:** 354 War Eagle Way, Wiggins Hall, Auburn, AL 36849
  - *(NOTE: For three or more authors, please copy/paste additional Author lines)*

- **Funding information, if any** *(how it should appear in the acknowledgements)*
  - **Funder:** FAA; **Grant number:**12-C-AM-AU-005
    - *(NOTE: For more than one funder, please copy/paste additional Funding lines)*

DESCRIPTION OF TECHNIQUES USED FOR DATA COLLECTION

(For reproducibility purposes, the publishing journal requires that we specify all details of each technique used. If you have any questions or concerns, contact SurfaceTopographyChallenge@gmail.com)

- **Number of techniques used:** 2

TECHNIQUE 1: [NOTE: Please copy/paste this section and repeat for each technique used]

(*Text only, please. We cannot accept citations in this document.)*

- **Common name of technique:** Optical
- **Type of technique:** Scanning white light interferometer
- **Instrument manufacturer:** Bruker, Billerica, MA
- **Instrument model:** Contour GTK
- **Tip size (if known):** N/A
- **Expected maximum lateral resolution:** 280 nm (manufacturer specification).
- **Data post-processing:** Tilt correction/ geometric plane fit
- **Sample preparation, if any:** Samples scanned as received but handled as such no contamination occurs to the surface

---

TECHNIQUE 2 (if applicable): [NOTE: Please copy/paste this section and repeat for each technique used]

(*Text only, please. We cannot accept citations in this document.)*

- **Common name of technique:** Contact profilometer
- **Type of technique:** stylus profilometer/tactile microscope
- **Instrument manufacturer:** Veeco, Plainview, NY (Now Bruker)
- **Instrument model:** Dektak 150
- **Tip size (if known):** 2µm
- **Expected maximum lateral resolution:** ~ 1µm (Depends on the size of the tip which can be exchanged)
- **Data post-processing:** Tilt correction/ curvature correction using polynomial fit

<u>AUTHOR AND TECHNIQUE INFORMATION FOR THE SURFACE-TOPOGRAPHY CHALLENGE</u>

Submit to: SurfaceTopographyChallenge@gmail.com ***(***Deadline: 29 Feb 2024***)***

- **Sample preparation, if any:** samples were handled minimally to avoid surface contamination and were scanned as received

AUTHOR AND TECHNIQUE INFORMATION FOR THE SURFACE-TOPOGRAPHY CHALLENGE

Submit to: SurfaceTopographyChallenge@gmail.com ***(***Deadline: 29 Feb 2024***)***

- We created this template to standardize the information we get from each group.
- Information submitted will be published *verbatim* in the Supplementary Information section.
- ***Please fill out all yellow-highlighted fields to the best of your ability.***

AUTHORSHIP INFORMATION (*Text only, please. We cannot accept citations in this document.)*

- **Author information (please ONLY include authors that directly contributed)**
  - **Author 1:** Felix Cassin
    - **ORCID ID:** 000-0002-4349-2848
    - **Address, Line 1:** Mechanical and Material Science Department, University of Pittsburgh
    - **Address, Line 2:** 3700 O'Hara St., Pittsburgh, PA 15261, United States
  - **Author 2:** Amit Kumar Prasad
    - **ORCID ID:** 0000-0002-6984-9112
    - **Address, Line 1:** Mechanical and Material Science Department, University of Pittsburgh
    - **Address, Line 2:** 3700 O'Hara St., Pittsburgh, PA 15261, United States
  - **Author 3:** Arushi Pradhan
    - **ORCID ID:** 0000-0001-8169-7900
    - **Address, Line 1:** Mechanical and Material Science Department, University of Pittsburgh
    - **Address, Line 2:** 3700 O'Hara St., Pittsburgh, PA 15261, United States
  - **Author 4:** Tevis D.B. Jacobs
    - **ORCID ID:** 0000-0001-8576-914X
    - **Address, Line 1:** Mechanical and Material Science Department, University of Pittsburgh
    - **Address, Line 2:** 3700 O'Hara St., Pittsburgh, PA 15261, United States
  - *(NOTE: For three or more authors, please copy/paste additional Author lines)*

- **Funding information, if any** *(how it should appear in the acknowledgements)*
  - **Funder:** National Science Foundation; **Grant number:** CAREER-1844739 and CMMI-2400999
    - *(NOTE: For more than one funder, please copy/paste additional Funding lines)*

DESCRIPTION OF TECHNIQUES USED FOR DATA COLLECTION

(For reproducibility purposes, the publishing journal requires that we specify all details of each technique used. If you have any questions or concerns, contact SurfaceTopographyChallenge@gmail.com)

- **Number of techniques used:** 5

TECHNIQUE 1: [NOTE: Please copy/paste this section and repeat for each technique used]

(*Text only, please. We cannot accept citations in this document.)*

- **Common name of technique:** Atomic Force Microscopy (AFM)
- **Type of technique:** Atomic Force Microscopy, tapping mode
- **Instrument manufacturer:** Bruker
- **Instrument model:** Dimension Icon
- **Tip size (if known):** 20 nm
- **Expected maximum lateral resolution:**
- **Data post-processing:** Tilt correction and 1st degree polynomial background removal for scans 20 and 50 µm
- **Sample preparation, if any:** sample cleaned with First Contact

---

TECHNIQUE 2: [NOTE: Please copy/paste this section and repeat for each technique used]

(*Text only, please. We cannot accept citations in this document.)*

- **Common name of technique:** Stylus
- **Type of technique:** Stylus Profilometer
- **Instrument manufacturer:** Bruker
- **Instrument model:** Surface Profiler DektakXT
- **Tip size (if known):** 2.59 µm
- **Expected maximum lateral resolution:**
- **Data post-processing:** none

- **Sample preparation, if any:** sample cleaned with First Contact

TECHNIQUE 3: [NOTE: Please copy/paste this section and repeat for each technique used]
(*Text only, please. We cannot accept citations in this document.)*

- **Common name of technique:** TEM
- **Type of technique:** Transmission Electron Microcopy
- **Instrument manufacturer:** JEOL, Tokyo, Japan
- **Instrument model:** 2100F, JEOL
- **Tip size (if known):**
- **Expected maximum lateral resolution:** 0.23 nm
- **Data post-processing:** data traced from TEM image using custom MATLAB script
- **Sample preparation, if any:** samples were cross-sectioned and prepped for TEM using standard metallographic practices for creating cross-sections

TECHNIQUE 4: [NOTE: Please copy/paste this section and repeat for each technique used]
(*Text only, please. We cannot accept citations in this document.)*

- **Common name of technique:** SEM
- **Type of technique:** Scanning Electron Microscopy
- **Instrument manufacturer:** Zeiss, Oberkochen, Germany
- **Instrument model:** Sigma VP
- **Tip size (if known):**
- **Expected maximum lateral resolution:** 10kx – 250 nm, 50kx – 50 nm, 250kx – 10 nm, 500kx – 5 nm
- **Data post-processing:** data traced from SEM image using custom MATLAB script
- **Sample preparation, if any:** sample cross-sectioned and prepped for SEM using standard procedure

TECHNIQUE 5: [NOTE: Please copy/paste this section and repeat for each technique used]
(*Text only, please. We cannot accept citations in this document.)*

- **Common name of technique:** Scanning White light Interferometer
- **Type of technique:** White Light Interferometry
- **Instrument manufacturer:** Bruker, Billerica, MA
- **Instrument model:** Contour GT-I
- **Tip size (if known):**
- **Expected maximum lateral resolution:** 2 µm
- **Data post-processing:** none
- **Sample preparation, if any:** sample cleaned with First Contact

AUTHOR AND TECHNIQUE INFORMATION FOR THE SURFACE-TOPOGRAPHY CHALLENGE

Submit to: SurfaceTopographyChallenge@gmail.com ***(***Deadline: 29 Feb 2024***)***

- We created this template to standardize the information we get from each group.
- Information submitted will be published *verbatim* in the Supplementary Information section.
- ***Please fill out all yellow-highlighted fields to the best of your ability.***

AUTHORSHIP INFORMATION (*Text only, please. We cannot accept citations in this document.)*

- **Author information (please ONLY include authors that directly contributed)**
  - **Author 1:** Kaiser, Fabian
    - **ORCID ID:** 0009-0003-7525-7062
    - **Address, Line 1:** Tribology Department, Freudenberg Technology Innovation SE & Co.KG
    - **Address, Line 2:** Hoehnerweg 2-4, 69469 Weinheim, Germany
  - **Author 2:** Savio, Daniele
    - **ORCID ID:** 0000-0003-1908-2379
    - **Address, Line 1:** Tribology Department, Freudenberg Technology Innovation SE & Co.KG
    - **Address, Line 2:** Hoehnerweg 2-4, 69469 Weinheim, Germany
  - **Author 3:** Miller, Theresa
    - **ORCID ID:** not available
    - **Address, Line 1:** Characterization Department, Freudenberg Technology Innovation SE & Co.KG
    - **Address, Line 2:** Hoehnerweg 2-4, 69469 Weinheim, Germany

- **Funding information, if any** *(how it should appear in the acknowledgements)*
    - **Funder:** not applicable

DESCRIPTION OF TECHNIQUES USED FOR DATA COLLECTION

(For reproducibility purposes, the publishing journal requires that we specify all details of each technique used. If you have any questions or concerns, contact SurfaceTopographyChallenge@gmail.com)

- **Number of techniques used:** 3

TECHNIQUE 1: [NOTE: Please copy/paste this section and repeat for each technique used]

(*Text only, please. We cannot accept citations in this document.)*

- **Common name of technique:** AFM
- **Type of technique:** atomic force microscope, tapping mode
- **Instrument manufacturer:** Park Systems, Suwon, South-Korea
- **Instrument model:** Park Systems NX10
- **Tip size (if known):** 30 nm radius
- **Expected maximum lateral resolution:** not known
- **Data post-processing:** none, raw instrument data was provided
- **Sample preparation, if any:** Sprayed with acetone, dried with a lint-free cloth

---

TECHNIQUE 2 (if applicable): [NOTE: Please copy/paste this section and repeat for each technique used]

(*Text only, please. We cannot accept citations in this document.)*

- **Common name of technique:** WLI
- **Type of technique:** white light interferometer
- **Instrument manufacturer:** Bruker, Billerica, MA, USA
- **Instrument model:** Bruker NPFlex
- **Tip size (if known):** N/A
- **Expected maximum lateral resolution:** 0.638 µm
- **Data post-processing:** none, raw instrument data was provided
- **Sample preparation, if any:** Sprayed with acetone, dried with a lint-free cloth

AUTHOR AND TECHNIQUE INFORMATION FOR THE SURFACE-TOPOGRAPHY CHALLENGE

Submit to: SurfaceTopographyChallenge@gmail.com ***(***Deadline: 29 Feb 2024***)***

TECHNIQUE 3 (if applicable): [NOTE: Please copy/paste this section and repeat for each technique used]

(*Text only, please. We cannot accept citations in this document.)*

- **Common name of technique:** Stylus
- **Type of technique:** stylus profilometer
- **Instrument manufacturer:** Mitutoyo, Kawasaki, Japan
- **Instrument model:** Mitutoyo Surftest SJ-500
- **Tip size (if known):** 2 µm
- **Expected maximum lateral resolution:** unknown
- **Data post-processing:** none, raw instrument data was provided
- **Sample preparation, if any:** Sprayed with acetone, dried with a lint-free cloth

AUTHOR AND TECHNIQUE INFORMATION FOR THE SURFACE-TOPOGRAPHY CHALLENGE

Submit to: SurfaceTopographyChallenge@gmail.com ***(***Deadline: 29 Feb 2024***)***

- We created this template to standardize the information we get from each group.
- Information submitted will be published *verbatim* in the Supplementary Information section.
- ***Please fill out all yellow-highlighted fields to the best of your ability.***

AUTHORSHIP INFORMATION (*Text only, please. We cannot accept citations in this document.)*

- **Author information (please ONLY include authors that directly contributed)**
    - **Author 1:** Požar, Tomaž
        - **ORCID ID:** 0000-0003-2524-3650
        - **Address, Line 1:** Laboratory for Tribology and Interface Nanotechnology (TINT), Faculty of Mechanical Engineering, University of Ljubljana
        - **Address, Line 2:** Aškerčeva cesta 6, Ljubljana, 1000, Slovenia
    - **Author 2:** Polajnar, Marko
        - **ORCID ID:** 0000-0002-1753-1655
        - **Address, Line 1:** Laboratory for Tribology and Interface Nanotechnology (TINT), Faculty of Mechanical Engineering, University of Ljubljana
        - **Address, Line 2:** Aškerčeva cesta 6, Ljubljana, 1000, Slovenia
    - **Author 3:** Samodurova, Anastasia
        - **ORCID ID:** 0009-0006-4799-7970
        - **Address, Line 1:** Laboratory for Tribology and Interface Nanotechnology (TINT), Faculty of Mechanical Engineering, University of Ljubljana
        - **Address, Line 2:** Aškerčeva cesta 6, Ljubljana, 1000, Slovenia
    - **Author 4:** Kalin, Mitjan
        - **ORCID ID:** 0000-0002-5938-1106
        - **Address, Line 1:** Laboratory for Tribology and Interface Nanotechnology (TINT), Faculty of Mechanical Engineering, University of Ljubljana
        - **Address, Line 2:** Aškerčeva cesta 6, Ljubljana, 1000, Slovenia

- **Funding information, if any** *(how it should appear in the acknowledgements)*
    - **Funder:** Slovenian Research and Innovation Agency; **Grant number:** P2-0231
        - *(NOTE: For more than one funder, please copy/paste additional Funding lines)*

DESCRIPTION OF TECHNIQUES USED FOR DATA COLLECTION

(For reproducibility purposes, the publishing journal requires that we specify all details of each technique used. If you have any questions or concerns, contact SurfaceTopographyChallenge@gmail.com)

- **Number of techniques used:** 3

TECHNIQUE 1: [NOTE: Please copy/paste this section and repeat for each technique used]

(*Text only, please. We cannot accept citations in this document.)*

- **Common name of technique:** Stylus
- **Type of technique:** Stylus profilometer
- **Instrument manufacturer:** Taylor Hobson, Leicester, UK
- **Instrument model:** Form Talysurf® i-Series PRO (1 mm gauge range)
- **Tip size (if known):** tip radius 2.0 µm (diamond conisphere stylus tip 112-2009)
- **Expected maximum lateral resolution:** approximately 3 µm (for smooth samples) and 7 µm (for rough samples), (line separation for surface scans: 0.1 µm)
- **Data post-processing:** None
- **Sample preparation, if any:** No cleaning. All samples were stored and measured under ambient conditions (25.0 ± 0.5) °C and relative humidity of (40 ± 10) %. All samples were bonded to an object glass to ensure to remain fixed during the measurements.

TECHNIQUE 2 (if applicable): [NOTE: Please copy/paste this section and repeat for each technique used]

(*Text only, please. We cannot accept citations in this document.)*

- **Common name of technique:** Optical interferometer
- **Type of technique:** Coherence scanning interferometry (for rough samples) and Phase-shifting interferometer (for smooth samples)

AUTHOR AND TECHNIQUE INFORMATION FOR THE SURFACE-TOPOGRAPHY CHALLENGE

Submit to: SurfaceTopographyChallenge@gmail.com ***(***Deadline: 29 Feb 2024***)***

- **Instrument manufacturer:** Bruker, Billerica, MA, USA
- **Instrument model:** ContourGT-K0 (Light source: green diode at 515.0 nm)
- **Tip size (if known):** N/A
- **Expected maximum lateral resolution:** 3.8 µm for 2.5x objective and 0.49 µm for 50x objective (Sparrow criterion)
- **Data post-processing:** A reference plane was constructed using a supersmooth SiC reference mirror (Bruker) averaging over 16 positions using 16 repetitions at each position to further reduce noise. This reference plane was subtracted from each measurement. Each sample was measured at two different positions either as a single shot or 100x acquisition using both objectives. In case of 100x acquisition, averaging over 100 repetitions was performed to obtain a single surface.
- **Sample preparation, if any:** No cleaning. All samples were stored and measured under ambient conditions (25.0 ± 0.5) °C and relative humidity of (40 ± 10) %. All samples were bonded to an object glass to ensure to remain fixed during the measurements.

TECHNIQUE 3: [NOTE: Please copy/paste this section and repeat for each technique used]

(*Text only, please. We cannot accept citations in this document.)*

- **Common name of technique:** AFM
- **Type of technique:** Atomic force microscope (contact mode)
- **Instrument manufacturer:** Asylum Research, Santa Barbara, CA, USA
- **Instrument model:** MFP-3D Origin
- **Tip size (if known):** nominal tip radius 7 nm (tapered rectangular AC240TS-R3 silicon probe)
- **Expected maximum lateral resolution:** approximately 10 nm
- **Data post-processing:** None
- **Sample preparation, if any:** No cleaning. All samples were stored and measured under ambient conditions (25.0 ± 0.5) °C and relative humidity of (40 ± 10) %. All samples were bonded to an object glass to ensure to remain fixed during the measurements.

AUTHOR AND TECHNIQUE INFORMATION FOR THE SURFACE-TOPOGRAPHY CHALLENGE

Submit to: SurfaceTopographyChallenge@gmail.com ***(***Deadline: 29 Feb 2024***)***

- We created this template to standardize the information we get from each group.
- Information submitted will be published *verbatim* in the Supplementary Information section.
- ***Please fill out all yellow-highlighted fields to the best of your ability.***

AUTHORSHIP INFORMATION (*Text only, please. We cannot accept citations in this document.)*

- **Author information (please ONLY include authors that directly contributed)**

  - **Author 1:** Yu-Sheng Li
    - **ORCID ID:** 0000-0002-1472-8153
    - **Address, Line 1:** Department of Chemical Engineering and Materials Research Institute, Pennsylvania State University
    - **Address, Line 2:** University Park, PA 16802, United States
  - **Author 2:** Seokhoon Jang
    - **ORCID ID:** 0000-0002-9198-7853
    - **Address, Line 1:** Department of Chemical Engineering and Materials Research Institute, Pennsylvania State University
    - **Address, Line 2:** University Park, PA 16802, United States
  - **Author 3:** Seong H. Kim
    - **ORCID ID:** 0000-0002-8575-7269
    - **Address, Line 1:** Department of Chemical Engineering and Materials Research Institute, Pennsylvania State University
    - **Address, Line 2:** University Park, PA 16802, United States

  - *(NOTE: For three or more authors, please copy/paste additional Author lines)*

- **Funding information, if any** *(how it should appear in the acknowledgements)*
  - **Funder:** National Science Foundation; **Grant number:** Grant No. CMMI-2038494, CMMI-2038499, and CMMI-1912199
    - *(NOTE: For more than one funder, please copy/paste additional Funding lines)*

DESCRIPTION OF TECHNIQUES USED FOR DATA COLLECTION

(For reproducibility purposes, the publishing journal requires that we specify all details of each technique used. If you have any questions or concerns, contact SurfaceTopographyChallenge@gmail.com)

- **Number of techniques used:** 1

TECHNIQUE 1: [NOTE: Please copy/paste this section and repeat for each technique used]

(*Text only, please. We cannot accept citations in this document.)*

- **Common name of technique:** Atomic Force Microscopy (AFM)
- **Type of technique:** Atomic Force Microscopy, tapping mode
- **Instrument manufacturer:** Digital instrument, USA
- **Instrument model:** Digital instrument, Multimode
- **Tip size (if known):** 8 nm
- **Expected maximum lateral resolution:** 8nm
- **Data post-processing:** Tilt correction
- **Sample preparation, if any:** None

AUTHOR AND TECHNIQUE INFORMATION FOR THE SURFACE-TOPOGRAPHY CHALLENGE

Submit to: SurfaceTopographyChallenge@gmail.com ***(***Deadline: 29 Feb 2024***)***

- We created this template to standardize the information we get from each group.
- Information submitted will be published *verbatim* in the Supplementary Information section.
- ***Please fill out all yellow-highlighted fields to the best of your ability.***

AUTHORSHIP INFORMATION (*Text only, please. We cannot accept citations in this document.)*

- **Author information (please ONLY include authors that directly contributed)**
  - **Author 1:** Nečas, David
    - **ORCID ID:** 0000-0001-7731-8453
    - **Address, Line 1:** CEITEC, Brno University of Technology
    - **Address, Line 2:** Purkyňova 123, Brno, 61200 Czech Republic
  - **Author 2:** Klapetek, Petr
    - **ORCID ID:** 0000-0001-5241-9178
    - **Address, Line 1:** Czech Metrology Institute
    - **Address, Line 2:** Okružní 31, Brno, 63800, Czech Republic
    - &
    - **Address, Line 1:** CEITEC, Brno University of Technology
    - **Address, Line 2:** Purkyňova 123, Brno, 61200 Czech Republic
  - **Author 3:** Valtr, Miroslav
    - **ORCID ID:** 0000-0002-7628-9184
    - **Address, Line 1:** Czech Metrology Institute
    - **Address, Line 2:** Okružní 31, Brno, 63800, Czech Republic
    - &
    - **Address, Line 1:** CEITEC, Brno University of Technology
    - **Address, Line 2:** Purkyňova 123, Brno, 61200 Czech Republic
  - *(NOTE: For three or more authors, please copy/paste additional Author lines)*

- **Funding information, if any** *(how it should appear in the acknowledgements)*
  - **Funder:** Czech Science Foundation; **Grant number:** GACR 21-12132J
  - **Funder:** Technology Agency of the Czech Republic, **Grant number:** TN02000020
    - *(NOTE: For more than one funder, please copy/paste additional Funding lines)*

DESCRIPTION OF TECHNIQUES USED FOR DATA COLLECTION

(For reproducibility purposes, the publishing journal requires that we specify all details of each technique used. If you have any questions or concerns, contact SurfaceTopographyChallenge@gmail.com)

- **Number of techniques used:** 2

TECHNIQUE 1: [NOTE: Please copy/paste this section and repeat for each technique used]

(*Text only, please. We cannot accept citations in this document.)*

- **Common name of technique:** Scanning Probe Microscopy
- **Type of technique:** atomic force microscope
- **Instrument manufacturer:** SIOS, Ilmenau, Germany
- **Instrument model:** NMM-1
- **Tip size (if known):** *not measured*
- **Expected maximum lateral resolution:** 50 nm (for this particular measurement)
- **Data post-processing:** moderate downsampling and rasterisation (during data conversion from raw NMM format to a format accepted by contact.enginnering)
- **Sample preparation, if any:** None

TECHNIQUE 2 (if applicable): [NOTE: Please copy/paste this section and repeat for each technique used]

(*Text only, please. We cannot accept citations in this document.)*

- **Common name of technique:** Scanning Probe Microscopy
- **Type of technique:** atomic force microscope
- **Instrument manufacturer:** Bruker, Santa Barbara, USA
- **Instrument model:** Dimension ICON
- **Tip size (if known):** *not measured*

AUTHOR AND TECHNIQUE INFORMATION FOR THE SURFACE-TOPOGRAPHY CHALLENGE

Submit to: SurfaceTopographyChallenge@gmail.com ***(***Deadline: 29 Feb 2024***)***

- **Expected maximum lateral resolution:** 8 nm (for this particular measurement)
- **Data post-processing:** None
- **Sample preparation, if any:** None

AUTHOR AND TECHNIQUE INFORMATION FOR THE SURFACE-TOPOGRAPHY CHALLENGE

Submit to: SurfaceTopographyChallenge@gmail.com ***(***Deadline: 29 Feb 2024***)***

- We created this template to standardize the information we get from each group.
- Information submitted will be published *verbatim* in the Supplementary Information section.
- ***Please fill out all yellow-highlighted fields to the best of your ability.***

AUTHORSHIP INFORMATION (*Text only, please. We cannot accept citations in this document.)*

- **Author information (please ONLY include authors that directly contributed)**
  - **Author 1:** Van Meter, Kylie E.
    - **ORCID ID:** 0000-0002-1458-8664
    - **Address, Line 1:** FAMU-FSU College of Engineering, Mech. Eng. Dept.
    - **Address, Line 2:** 2003 Levy Ave, Tallahassee FL, 32310
  - **Author 2:** Krick, Brandon A.
    - **ORCID ID:** 0000-0003-3191-5433
    - **Address, Line 1:** FAMU-FSU College of Engineering, Mech. Eng. Dept.
    - **Address, Line 2:** 2003 Levy Ave, Tallahassee FL, 32310
  - *(NOTE: For three or more authors, please copy/paste additional Author lines)*

- **Funding information, if any** *(how it should appear in the acknowledgements)*
  - **Funder:** NSF CMMI MEP Career **Grant number:** #2027029
  - **Funder:** NSF CMMI MEP GOALI **Grant number:** #1463141
  - **Funder:** NSF GRFP **Grant number:** #1449440

DESCRIPTION OF TECHNIQUES USED FOR DATA COLLECTION

(For reproducibility purposes, the publishing journal requires that we specify all details of each technique used. If you have any questions or concerns, contact SurfaceTopographyChallenge@gmail.com)

- **Number of techniques used:** 1

TECHNIQUE 1: [NOTE: Please copy/paste this section and repeat for each technique used]

(*Text only, please. We cannot accept citations in this document.)*

- **Common name of technique:** Optical Profilometry
- **Type of technique:** Scanning White Light Interferometry
- **Instrument manufacturer:** Bruker, Billerica, MA, USA
- **Instrument model:** Bruker Contour GT
- **Tip size (if known):** N/A
- **Expected maximum lateral resolution:** 0.197 μm at 50x magnification.
- **Data post-processing:** Tilt correction.
- **Sample preparation, if any:** Sample surface cleaned with IPA.

AUTHOR AND TECHNIQUE INFORMATION FOR THE SURFACE-TOPOGRAPHY CHALLENGE

Submit to: SurfaceTopographyChallenge@gmail.com ***(***Deadline: 29 Feb 2024***)***

(*Text only, please. We cannot accept citations in this document.)*

- **Common name of technique:** WLI
- **Type of technique:** White light interferometer
- **Instrument manufacturer:** Sensofar, Terrassa, Spain
- **Instrument model:** Plμ Neox
- **Tip size (if known):** Non-contact instrument
- **Expected maximum lateral resolution:**
  - 0.46 μm for 10x magnification objective
  - 0.25 μm for 50x magnification objective
- **Data post-processing:** No data processing
- **Sample preparation, if any:** After undergoing two rounds of ultrasonic cleaning in isopropanol, each for a duration of 10 minutes, the samples were subsequently tested using all four methods within a single day.

TECHNIQUE 4 (if applicable): [NOTE: Please copy/paste this section and repeat for each technique used]

(*Text only, please. We cannot accept citations in this document.)*

- **Common name of technique:** Chromatic aberration
- **Type of technique:** Chromatic aberration
- **Instrument manufacturer:** Fries Research and Technology GmbH (now owned by Camtek), Bergisch Gladbach, Germany
- **Instrument model:** MicroProf
- **Tip size (if known):** Non-contact instrument
- **Expected maximum lateral resolution:** 2 μm
- **Data post-processing:** No data processing
- **Sample preparation, if any:** After undergoing two rounds of ultrasonic cleaning in isopropanol, each for a duration of 10 minutes, the samples were subsequently tested using all four methods within a single day.

AUTHOR AND TECHNIQUE INFORMATION FOR THE SURFACE-TOPOGRAPHY CHALLENGE

Submit to: SurfaceTopographyChallenge@gmail.com ***(***Deadline: 29 Feb 2024***)***

- We created this template to standardize the information we get from each group.
- Information submitted will be published *verbatim* in the Supplementary Information section.
- ***Please fill out all yellow-highlighted fields to the best of your ability.***

AUTHORSHIP INFORMATION (*Text only, please. We cannot accept citations in this document.)*

- **Author information (please ONLY include authors that directly contributed)**
  - **Author 1:** Sainsot, Philippe
    - **ORCID ID:** 0000-0001-6913-9770
    - **Address, Line 1:** Univ Lyon, INSA Lyon, CNRS, LaMCoS, UMR 5259
    - **Address, Line 2:** 20 Av A Einstein, 69621 Villeurbanne, France
  - **Author 2:** Lubrecht, Ton A
    - **ORCID ID:** 0000-0003-1274-6304
    - **Address, Line 1:** Univ Lyon, INSA Lyon, CNRS, LaMCoS, UMR 5259
    - **Address, Line 2:** 20 Av A Einstein, 69621 Villeurbanne, France
  - *(NOTE: For three or more authors, please copy/paste additional Author lines)*

- **Funding information, if any** *(how it should appear in the acknowledgements)*
  - **Funder:** none **Grant number:**
    - *(NOTE: For more than one funder, please copy/paste additional Funding lines)*

DESCRIPTION OF TECHNIQUES USED FOR DATA COLLECTION

(For reproducibility purposes, the publishing journal requires that we specify all details of each technique used. If you have any questions or concerns, contact SurfaceTopographyChallenge@gmail.com)

- **Number of techniques used:** 1

TECHNIQUE 1: [NOTE: Please copy/paste this section and repeat for each technique used]

(*Text only, please. We cannot accept citations in this document.)*

- **Common name of technique:** interferometry
- **Type of technique:** white light interferometry, phase shifting
- **Instrument manufacturer:** Sensofar, Terrassa, Spain
- **Instrument model:** S-NEOX
- **Tip size (if known):** N/A
- **Expected maximum lateral resolution:** 0.28 µm
- **Data post-processing:** No
- **Sample preparation, if any:** mounted as flat as possible

---

TECHNIQUE 2 (if applicable): [NOTE: Please copy/paste this section and repeat for each technique used]

(*Text only, please. We cannot accept citations in this document.)*

- **Common name of technique:** <Generic name; not brand names>
- **Type of technique:** <Choose one of the following categories>
  - [atomic force microscope; white light interferometer chromatic aberration; digital 3D optical microscope; confocal laser scanning microscope; holographic measurement; angle-resolved spectroscopy; scanning electron microscope; reconstruction from scanning electron microscope; stylus profilometer/tactile microscope; transmission electron microscope; triboindenter; other (specify)]
- **Instrument manufacturer:** <Name, city, state>
- **Instrument model:** <Brand name and model number>
- **Tip size (if known):** <Insert "N/A" for non-contact instruments>
- **Expected maximum lateral resolution:** <Based on manufacturer specs or technique info>
- **Data post-processing:** <Briefly describe any processing you did (such as tilt-correction or artifact removal) prior to submission of the data – either on the collection instrument or in other software.>
- **Sample preparation, if any:** <Briefly describe any sample preparation (cleaning, hot-mounting, etc.)>

AUTHOR AND TECHNIQUE INFORMATION FOR THE SURFACE-TOPOGRAPHY CHALLENGE

Submit to: SurfaceTopographyChallenge@gmail.com ***(***Deadline: 29 Feb 2024***)***

- We created this template to standardize the information we get from each group.
- Information submitted will be published *verbatim* in the Supplementary Information section.
- ***Please fill out all yellow-highlighted fields to the best of your ability.***

AUTHORSHIP INFORMATION (*Text only, please. We cannot accept citations in this document.)*

- **Author information (please ONLY include authors that directly contributed)**
  - **Author 1:** Ma, Chengfu
    - **ORCID ID:** 0000-0002-6813-1459
    - **Address 1, Line 1:** Department of Precision Machinery and Precision Instrumentation, University of Science and Technology of China
    - **Address 1, Line 2:** Jinzhai Road 96, Hefei, Anhui, 230226, China
    - **Address 2, Line 1:** Chair of Materials Science and Nanotechnology, Technical University Dresden
    - **Address 2, Line 2:** Budapester Str. 27, Dresden, Saxony, 01069, Germany
  - *(NOTE: For three or more authors, please copy/paste additional Author lines)*

- **Funding information, if any** *(how it should appear in the acknowledgements)*
  - **Funder:** National Natural Science Foundation of China; **Grant number:** 52375556
    - *(NOTE: For more than one funder, please copy/paste additional Funding lines)*

DESCRIPTION OF TECHNIQUES USED FOR DATA COLLECTION

(For reproducibility purposes, the publishing journal requires that we specify all details of each technique used. If you have any questions or concerns, contact SurfaceTopographyChallenge@gmail.com)

- **Number of techniques used:** 1

TECHNIQUE 1: [NOTE: Please copy/paste this section and repeat for each technique used]

(*Text only, please. We cannot accept citations in this document.)*

- **Common name of technique:** AFM
- **Type of technique:** atomic force microscope, tapping mode
  - [atomic force microscope; white light interferometer chromatic aberration; digital 3D optical microscope; confocal laser scanning microscope; holographic measurement; angle-resolved spectroscopy; scanning electron microscope; reconstruction from scanning electron microscope; stylus profilometer/tactile microscope; transmission electron microscope; triboindenter; other (specify)]
- **Instrument manufacturer:** Bruker/Veeco, Santa Barbara, California
- **Instrument model:** Veeco Multimode IIIA, J Scanner
- **Tip size (if known):** 10 nm curvature radius (nominal)
- **Expected maximum lateral resolution:** ~ 1 nm
- **Data post-processing:** No
- **Sample preparation, if any:** No

AUTHOR AND TECHNIQUE INFORMATION FOR THE SURFACE-TOPOGRAPHY CHALLENGE

Submit to: SurfaceTopographyChallenge@gmail.com ***(***Deadline: 29 Feb 2024***)***

- We created this template to standardize the information we get from each group.
- Information submitted will be published *verbatim* in the Supplementary Information section.
- ***Please fill out all yellow-highlighted fields to the best of your ability.***

AUTHORSHIP INFORMATION (*Text only, please. We cannot accept citations in this document.*)

- **Author information (please ONLY include authors that directly contributed)**
  - **Author 1:** Farouk Maaboudallah
    - **ORCID ID:** 0000-0003-4777-9835
    - **Address, Line 1:** Department of mechanical engineering, Université de Sherbrooke
    - **Address, Line 2:** 2500 Bd de l'Université, QC J1K 2R1, Canada
  - **Author 2:** Mohamed Najah
    - **ORCID ID:** 0000-0003-0632-1174
    - **Address, Line 1:** Department of electrical engineering, Université de Sherbrooke
    - **Address, Line 2:** 2500 Bd de l'Université, QC J1K 2R1, Canada

- **Funding information, if any** *(how it should appear in the acknowledgements)*
  - **Funder:** The project is unfunded

DESCRIPTION OF TECHNIQUES USED FOR DATA COLLECTION

(For reproducibility purposes, the publishing journal requires that we specify all details of each technique used. If you have any questions or concerns, contact SurfaceTopographyChallenge@gmail.com)

- **Number of techniques used:** 2

TECHNIQUE 1: [NOTE: Please copy/paste this section and repeat for each technique used]

(*Text only, please. We cannot accept citations in this document.*)

- **Common name of technique:** Contact-based microscope - Atomic Force Microscopy
- **Type of technique:** Atomic Force Microscopy (AFM), Tapping Mode.
- **Instrument manufacturer:** Park System Inc., Santa Clara, California.
- **Instrument model:** Park System NX20.
- **Tip size (if known):** SSS-NCHR probes – R < 2 nm (< 5 nm guaranteed).
- **Expected maximum lateral resolution:** 4096 x 4096 pixels.
- **Data post-processing:** *N/A*
- **Sample preparation, if any:** Samples were cleaned by immersion for 1 min in acetone, isopropanol, and deionized water and dried using N_2 gun prior to the surface topography measurements.

TECHNIQUE 2 (if applicable): [NOTE: Please copy/paste this section and repeat for each technique used]

(*Text only, please. We cannot accept citations in this document.*)

- **Common name of technique:** Microscope based.
- **Type of technique:** Confocal Laser Scanning Microscopy (CLSM).
- **Instrument manufacturer:** Keyence, Osaka, Japan.
- **Instrument model:** Keyence VK-X 1100 series
- **Tip size (if known):** N/A
- **Expected maximum lateral resolution:** 150X lens allowing a pixel size of 47.5 nm.
- **Data post-processing:** *N/A*
- **Sample preparation, if any:** Samples were cleaned by immersion for 1 min in acetone, isopropanol, and deionized water and dried using N_2 gun prior to the surface topography measurements.

AUTHOR AND TECHNIQUE INFORMATION FOR THE SURFACE-TOPOGRAPHY CHALLENGE

Submit to: SurfaceTopographyChallenge@gmail.com ***(***Deadline: 29 Feb 2024***)***

- We created this template to standardize the information we get from each group.
- Information submitted will be published *verbatim* in the Supplementary Information section.
- ***Please fill out all yellow-highlighted fields to the best of your ability.***

AUTHORSHIP INFORMATION (*Text only, please. We cannot accept citations in this document.)*

- **Author information (please ONLY include authors that directly contributed)**
  - **Author 1:** Edwards, Camille
    - **ORCID ID:** 0000-0002-3610-5264
    - **Address, Line 1:** Walker Department of Mechanical Engineering, The University of Texas at Austin
    - **Address, Line 2:** 204 E. Dean Keeton, Austin, TX, 78712-1591, USA
  - **Author 2:** Johnson, Owen
    - **ORCID ID:** 0009-0007-3025-7068
    - **Address, Line 1:** Walker Department of Mechanical Engineering, The University of Texas at Austin
    - **Address, Line 2:** 204 E. Dean Keeton, Austin, TX, 78712-1591, USA
  - **Author 3:** Mangolini, Filippo
    - **ORCID ID:** 0000-0003-3360-9122
    - **Address, Line 1:** Walker Department of Mechanical Engineering & Texas Materials Institute, The University of Texas at Austin
    - **Address, Line 2:** 204 E. Dean Keeton, Austin, TX, 78712-1591, USA

- **Funding information, if any** *(how it should appear in the acknowledgements)*
  - **Funder: National Science Foundation**; **Grant number:** 2042304
  - **Funder: Welch Foundation**; **Grant number:** F-2151-2023040

DESCRIPTION OF TECHNIQUES USED FOR DATA COLLECTION

(For reproducibility purposes, the publishing journal requires that we specify all details of each technique used. If you have any questions or concerns, contact SurfaceTopographyChallenge@gmail.com)

- **Number of techniques used:** 1

TECHNIQUE 1: [NOTE: Please copy/paste this section and repeat for each technique used]

(*Text only, please. We cannot accept citations in this document.)*

- **Common name of technique:** AFM
- **Type of technique:** Atomic force microscope operated in repulsive tapping mode
- **Instrument manufacturer:** Asylum Research, Santa Barbara, CA, USA
- **Instrument model:** Asylum Research MFP-3D AFM
- **Tip size (if known):** 16±3 nm (HQ:NSC14/Hard/Al BS. DLC-coated tips were used to minimize tip wear during imaging. The tip radius was determined using the blind tip reconstruction method with a ultrananocrystallize diamond surface (UNCD, Aqua 25, Advanced Diamond Technologies, USA) as testing substrate).
- **Expected maximum lateral resolution:** ~20 nm
- **Data post-processing:** Raw images were submitted without post-processing.
- **Sample preparation, if any:** A52 and P78 were cleaned in a UV ozone cleaner for 1 minute. P78 was sonicated for an additional 5 minutes in ethanol.

AUTHOR AND TECHNIQUE INFORMATION FOR THE SURFACE-TOPOGRAPHY CHALLENGE

Submit to: SurfaceTopographyChallenge@gmail.com ***(***Deadline: 29 Feb 2024***)***

- We created this template to standardize the information we get from each group.
- Information submitted will be published *verbatim* in the Supplementary Information section.
- ***Please fill out all yellow-highlighted fields to the best of your ability.***

AUTHORSHIP INFORMATION (*Text only, please. We cannot accept citations in this document.)*

- **Author information (please ONLY include authors that directly contributed)**

  - **Author 1:** Zhang, Xuhui
    - **ORCID ID:** 0009-0000-7280-6060
    - **Address, Line 1:** Department of Civil and Environmental Engineering, University of Illinois at Urbana-Champaign
    - **Address, Line 2:** 205 N. Mathews Ave, Urbana, IL 61801, USA
  - **Author 2:** Yus, Joaquin
    - **ORCID ID:** 0000-0002-9457-4042
    - **Address, Line 1:** Carl R. Woese Institute for Genomic Biology & Department of Mechanical Science and Engineering, University of Illinois at Urbana Champaign
    - **Address, Line 2:** 1206 W Gregory Dr, Urbana, IL 61801, USA
  - **Author 3:** Lee, Ming Jun
    - **ORCID ID:** 0000-0002-8385-9720
    - **Address, Line 1:** Department of Materials Science and Engineering, University of Illinois at Urbana-Champaign
    - **Address, Line 2:** 1304 W. Green St., Urbana, IL 61801, USA
  - **Author 4:** Sun, Kangdi
    - **ORCID ID:** 0000-0002-7919-0673
    - **Address, Line 1:** Department of Materials Science and Engineering, University of Illinois at Urbana-Champaign
    - **Address, Line 2:** 1304 W. Green St., Urbana, IL 61801, USA
  - **Author 5:** Deptula, Alexander J.
    - **ORCID ID:** 0000-0002-4811-247X
    - **Address, Line 1:** Department of Materials Science and Engineering, University of Illinois at Urbana-Champaign
    - **Address, Line 2:** 1304 W. Green St., Urbana, IL 61801, USA
  - **Author 6:** Greenwood, Gus
    - **ORCID ID:** 0000-0001-8878-0467
    - **Address, Line 1:** Department of Civil and Environmental Engineering, University of Illinois at Urbana-Champaign
    - **Address, Line 2:** 205 N. Mathews Ave, Urbana, IL 61801, USA
  - **Author 7:** Li, Jingyu
    - **ORCID ID:** 0009-0006-9633-9114
    - **Address, Line 1:** Department of Civil and Environmental Engineering, University of Illinois at Urbana-Champaign
    - **Address, Line 2:** 205 N. Mathews Ave, Urbana, IL 61801, USA
  - **Author 8:** Zheng, Qianlu
    - **ORCID ID:** 0009-0005-3337-3115
    - **Address, Line 1:** Department of Civil and Environmental Engineering, University of Illinois at Urbana-Champaign
    - **Address, Line 2:** 205 N. Mathews Ave, Urbana, IL 61801, USA
  - **Author 9:** Ma, Yongjian
    - **ORCID ID:** 0000-0001-8374-9401
    - **Address, Line 1:** Department of Civil and Environmental Engineering, University of Illinois at Urbana-Champaign
    - **Address, Line 2:** 205 N. Mathews Ave, Urbana, IL 61801, USA
  - **Author 10:** Espinosa-Marzal, Rosa M.
    - **ORCID ID:** 0000-0003-3442-2511

AUTHOR AND TECHNIQUE INFORMATION FOR THE SURFACE-TOPOGRAPHY CHALLENGE

Submit to: SurfaceTopographyChallenge@gmail.com ***(***Deadline: 29 Feb 2024***)***

- **Address, Line 1:** Department of Materials Science and Engineering, University of Illinois at Urbana-Champaign
- **Address, Line 2:** 1304 W. Green St., Urbana, IL 61801, USA
- **Address, Line 3:** Department of Civil and Environmental Engineering, University of Illinois at Urbana-Champaign
- **Address, Line 4:** 205 N. Matthews Ave, Urbana, IL 61801, USA

DESCRIPTION OF TECHNIQUES USED FOR DATA COLLECTION

(For reproducibility purposes, the publishing journal requires that we specify all details of each technique used. If you have any questions or concerns, contact SurfaceTopographyChallenge@gmail.com)

- **Number of techniques used:** 6

TECHNIQUE 1:

- **Common name of technique:** AFM
- **Type of technique:** Atomic force microscope, tapping mode
- **Instrument manufacturer:** JPK Instruments, Berlin, Germany
- **Instrument model:** Nanowizard
- **Tip size (if known):** 10 nm (Tap300Al-G, BudgetSensors)
- **Expected maximum lateral resolution:** 10 nm
- **Data post-processing:** All data files were uploaded to contact.engineering as-provided by the instrument with no post-processing.
- **Sample preparation, if any:** Samples were cleaned by immersion in acetone, isopropanol, and ethanol in a ultrasonic bath for 15 min each.

TECHNIQUE 2:

- **Common name of technique:** 3D optical profilometry
- **Type of technique:** LASER Profilometer
- **Instrument manufacturer:** Keyence, Itasca, IL 60143, USA
- **Instrument model:** Keyence VK-X1000 3D Laser Scanning Confocal Microscope
- **Tip Size (if known):** 404 nm laser
- **Expected maximum resolution:** 20 nm
- **Data post-processing:** Curvature and tilt correction.
- **Sample preparation, if any:** Samples were cleaned by immersion in acetone, isopropanol, and ethanol in a ultrasonic bath for 15 min each.

TECHNIQUE 3:

- **Common name of technique:** AFM
- **Type of technique:** Atomic force microscope, Quantitative imaging (fast force-mapping)
- **Instrument manufacturer:** JPK Instruments, Berlin, Germany
- **Instrument model:** Nanowizard
- **Tip size (if known):** 8 nm (HQ:CSC37/No Al, MikroMash, nominal spring constant 0.6-1.2 N m−1)
- **Expected maximum lateral resolution:** 8 nm
- **Data post-processing:** All data files were uploaded to contact.engineering as-provided by the instrument with no post-processing.
- **Sample preparation, if any:** Samples were cleaned by immersion in acetone, isopropanol, and ethanol in a ultrasonic bath for 15 min each.

TECHNIQUE 4:

- **Common name of technique:** AFM
- **Type of technique:** Atomic force microscope, contact mode
- **Instrument manufacturer:** JPK Instruments, Berlin, Germany
- **Instrument model:** Nanowizard

- **Tip size (if known):** 20 nm (HQ:CSC37/No Al, MikroMash, nominal spring constant 0.6-1.2 N m^{-1})
- **Expected maximum lateral resolution:** 20 nm
- **Data post-processing:** All data files were uploaded to contact.engineering as-provided by the instrument with no post-processing.
- **Sample preparation, if any:** Samples were cleaned by immersion in acetone, isopropanol, and ethanol in a ultrasonic bath for 15 min each.

TECHNIQUE 5:

- **Common name of technique:** ChemiSEM
- **Type of technique:** Scanning electron microscopy
- **Instrument manufacturer:** ThermoFisher Scientific, US
- **Instrument model:** Axia ChemiSEM
- **Tip Size (if known):** N/A
- **Expected maximum resolution:** 3.0nm
- **Data post-processing:** All data files were uploaded as provided by the instrument with no post-processing.
- **Sample preparation, if any:** Samples were cleaned by immersion in acetone, isopropanol, and ethanol in a ultrasonic bath for 15 min each.

TECHNIQUE 6:

- **Common name of technique:** SEM
- **Type of technique:** Scanning electron microscope
- **Instrument manufacturer:** Hitachi, Japan
- **Instrument model:** HITACHI S-4800
- **Tip size (if known):** N/A
- **Expected maximum lateral resolution:** 1 nm
- **Data post-processing:** All data files were uploaded to contact.engineering as-provided by the instrument with no post-processing.
- **Sample preparation, if any:** Samples were cleaned by immersion in acetone, isopropanol, and ethanol in a ultrasonic bath for 15 min each.

AUTHORSHIP INFORMATION (*Text only, please. We cannot accept citations in this document.)*

- **Author information (please ONLY include authors that directly contributed)**
  - **Author 1:** Yonggang Meng
    - **ORCID ID:** 0000-0002-8274-5131
    - **Address, Line 1:** Department of Mechanical Engineering, Tsinghua University
    - **Address, Line 2:** No.1, Section 4, Chengfu Road, Haidian District, Beijing, 100084, China
  - **Author 2:** Tianbao Ma
    - **ORCID ID:** 0000-0001-8016-9241
    - **Address, Line 1:** Department of Mechanical Engineering, Tsinghua University
    - **Address, Line 2:** No.1, Section 4, Chengfu Road, Haidian District, Beijing, 100084, China

DESCRIPTION OF TECHNIQUES USED FOR DATA COLLECTION

(For reproducibility purposes, the publishing journal requires that we specify all details of each technique used.

If you have any questions or concerns, contact SurfaceTopographyChallenge@gmail.com)

- **Number of techniques used:** 5

TECHNIQUE 1: [NOTE: Please copy/paste this section and repeat for each technique used]

(*Text only, please. We cannot accept citations in this document.)*

- **Common name of technique:** AFM
- **Type of technique:** atomic force microscope, tapping mode
- **Instrument manufacturer:** Bruker, Billerica, MA, USA
- **Instrument model:** Dimension Icon
- **Tip size (if known):** 20 nm radius
- **Expected maximum lateral resolution:** 20 nm
- **Data post-processing:** Tilt correction and 1st degree polynomial background removal.
- **Sample preparation, if any:** Samples were cleaned with First Contact.

TECHNIQUE 2: [NOTE: Please copy/paste this section and repeat for each technique used]

(*Text only, please. We cannot accept citations in this document.)*

- **Common name of technique:** AFM
- **Type of technique:** atomic force microscope, tapping mode
- **Instrument manufacturer:** Oxford Instruments
- **Instrument model:** Cypher s
- **Tip size (if known):** 20 nm radius
- **Expected maximum lateral resolution:** 0.06 nm
- **Data post-processing:** Tilt correction and 1st degree flatten.
- **Sample preparation, if any:** Samples were cleaned with First Contact.

TECHNIQUE 3: [NOTE: Please copy/paste this section and repeat for each technique used]

(*Text only, please. We cannot accept citations in this document.)*

- **Common name of technique:** Confocal
- **Type of technique:** confocal laser scanning microscope
- **Instrument manufacturer:** Leica
- **Instrument model:** LSM900
- **Tip size (if known):** N/A
- **Expected maximum lateral resolution:** 0.125 µm
- **Data post-processing:** Remove curvature and tilt correction.
- **Sample preparation, if any:** Samples were cleaned with First Contact.

TECHNIQUE 4: [NOTE: Please copy/paste this section and repeat for each technique used]

(*Text only, please. We cannot accept citations in this document.)*

- **Common name of technique:** Confocal
- **Type of technique:** confocal laser scanning microscope
- **Instrument manufacturer:** RTEC
- **Instrument model:** UP-Lambda2
- **Tip size (if known):** N/A
- **Expected maximum lateral resolution:** 0.255 µm
- **Data post-processing:** Remove curvature and tilt correction.
- **Sample preparation, if any:** Samples were cleaned with First Contact.

TECHNIQUE 5: [NOTE: Please copy/paste this section and repeat for each technique used]

(*Text only, please. We cannot accept citations in this document.)*

- **Common name of technique:** Whitelight
- **Type of technique:** white light interferometer
- **Instrument manufacturer:** ZYGO
- **Instrument model:** Netview
- **Tip size (if known):** N/A
- **Expected maximum lateral resolution:** 0.081 µm
- **Data post-processing:** Remove curvature and tilt correction.
- **Sample preparation, if any:** Samples were cleaned with First Contact.

AUTHOR AND TECHNIQUE INFORMATION FOR THE SURFACE-TOPOGRAPHY CHALLENGE

Submit to: SurfaceTopographyChallenge@gmail.com ***(***Deadline: 29 Feb 2024***)***

- We created this template to standardize the information we get from each group.
- Information submitted will be published *verbatim* in the Supplementary Information section.
- ***Please fill out all yellow-highlighted fields to the best of your ability.***

AUTHORSHIP INFORMATION (*Text only, please. We cannot accept citations in this document.)*

- **Author information (please ONLY include authors that directly contributed)**
  - **Author 1:** Kumar, Nityanshu
    - **ORCID ID:** 0000-0002-4655-4653
    - **Address, Line 1:** Science and Technology Division, Corning Incorporated
    - **Address, Line 2:** 184 Science Center Dr, Painted Post, NY 14870, USA
  - **Author 2:** Kumar, Shubhendu
    - **ORCID ID:** 0000-0003-2658-2988
    - **Address, Line 1:** School of Polymer Science and Polymer Engineering, University of Akron
    - **Address, Line 2:** 170 University Ave., Akron, OH, 44325, USA
  - *(NOTE: For three or more authors, please copy/paste additional Author lines)*

- **Funding information, if any** *(how it should appear in the acknowledgements)*
  - **Funder:** N/A; **Grant number:** N/A
    - *(NOTE: For more than one funder, please copy/paste additional Funding lines)*

DESCRIPTION OF TECHNIQUES USED FOR DATA COLLECTION

(For reproducibility purposes, the publishing journal requires that we specify all details of each technique used. If you have any questions or concerns, contact SurfaceTopographyChallenge@gmail.com)

- **Number of techniques used:** 3

TECHNIQUE 1: [NOTE: Please copy/paste this section and repeat for each technique used]

(*Text only, please. We cannot accept citations in this document.)*

- **Common name of technique:** Stylus
- **Type of technique:** Stylus Profilometer
- **Instrument manufacturer:** Bruker, Billerica, MA, USA
- **Instrument model:** Bruker Dektak XT profilometer
- **Tip size (if known):** 2.00 µm radius (as per manufacturer)
- **Expected maximum lateral resolution:** 2.00 µm
- **Data post-processing:** No Post Processing before submission on contact.engineering
- **Sample preparation, if any:** Rinsed with acetone and then ethanol, Blow dried with dry N_2 gas before any measurement on the samples.

TECHNIQUE 2 (if applicable): [NOTE: Please copy/paste this section and repeat for each technique used]

(*Text only, please. We cannot accept citations in this document.)*

- **Common name of technique:** AFM
- **Type of technique:** Atomic Force Microscope (Tapping Mode)
- **Instrument manufacturer:** Asylum Research, Oxford Instruments, Santa Barbara, CA, USA
- **Instrument model:** MFP-3D Infinity
- **Tip size (if known):** 7 nm nominal radius (typical radius below 10 nm, Model: AC160TSA-R3)
- **Expected maximum lateral resolution:** 10 nm
- **Data post-processing:** No Post Processing before submission on contact.engineering
- **Sample preparation, if any:** Rinsed with acetone and then ethanol, Blow dried with dry N_2 gas before any measurement on the samples.

TECHNIQUE 3 (if applicable): [NOTE: Please copy/paste this section and repeat for each technique used]

(*Text only, please. We cannot accept citations in this document.)*

- **Common name of technique:** 3D Surface Profiler
- **Type of technique:** Laser Scanning Confocal Microscopy
- **Instrument manufacturer:** Keyence, Osaka, Japan

AUTHOR AND TECHNIQUE INFORMATION FOR THE SURFACE-TOPOGRAPHY CHALLENGE

Submit to: SurfaceTopographyChallenge@gmail.com ***(***Deadline: 29 Feb 2024***)***

- **Instrument model:** Keyence VK-X200
- **Tip size (if known):** NA
- **Expected maximum lateral resolution:** 0.5 µm - 2 µm (Based on magnification used, 0.5 µm for 150x)
- **Data post-processing:** Mean filtering was performed with a 3×3 square kernel to reduce the noise in the images.
- **Sample preparation, if any:** Rinsed with acetone and then ethanol, Blow dried with dry N_2 gas before any measurement on the samples.

AUTHOR AND TECHNIQUE INFORMATION FOR THE SURFACE-TOPOGRAPHY CHALLENGE

Submit to: SurfaceTopographyChallenge@gmail.com ***(***Deadline: 29 Feb 2024***)***

- We created this template to standardize the information we get from each group.
- Information submitted will be published *verbatim* in the Supplementary Information section.
- ***Please fill out all yellow-highlighted fields to the best of your ability.***

AUTHORSHIP INFORMATION

- **Author information**
  - **Author 1:** Giordano, Goffredo
    - **ORCID ID:** 0000-0002-5844-1496
    - **Address, Line 1:** Polytechnic University of Bari, Department of Mechanics Mathematics and Management
    - **Address, Line 2:** via Orabona 4, Bari 70125, Italy
  - **Author 2:** Ciavarella Michele
    - **ORCID ID:** 0000-0001-6271-0081
    - **Address, Line 1:** Polytechnic University of Bari, Department of Mechanics Mathematics and Management
    - **Address, Line 2:** via Orabona 4, Bari 70125, Italy
  - **Author 3:** Papangelo, Antonio
    - **ORCID ID:** 0000-0002-0214-904X
    - **Address, Line 1:** Polytechnic University of Bari, Department of Mechanics Mathematics and Management
    - **Address, Line 2:** via Orabona 4, Bari 70125, Italy

- **Funding information**
  - **Funder:** European Research Council; **Grant number:** 101039198
    - *A.P. and G.G. were supported by the European Union (ERC-2021-STG, "Towards Future Interfaces With Tuneable Adhesion By Dynamic Excitation" - SURFACE, Project ID: 101039198, Grant No. CUP: D95F22000430006). Views and opinions expressed are however those of the authors only and do not necessarily reflect those of the European Union or the European Research Council. Neither the European Union nor the granting authority can be held responsible for them.*
  - **Funder:** Italian Ministry of University and Research under the Programme "Department of Excellence" L. 232/2016; **Grant number:** CUP - D93C23000100001
    - *G.G., M.C., A.P. were partly supported by the Italian Ministry of University and Research under the Programme "Department of Excellence" Legge 232/2016 (Grant No. CUP - D93C23000100001).*

DESCRIPTION OF TECHNIQUES USED FOR DATA COLLECTION

(For reproducibility purposes, the publishing journal requires that we specify all details of each technique used. If you have any questions or concerns, contact SurfaceTopographyChallenge@gmail.com)

- **Number of techniques used:** 1

TECHNIQUE 1:

- **Common name of technique:** Digital Microscopy
- **Type of technique:** digital 3D optical microscope;
- **Instrument manufacturer:** Keyence, Osaka, Japan
- **Instrument model:** Keyence, Digital Microscope,VHX-7000N
- **Tip size (if known):** N/A
- **Expected maximum lateral resolution:** Limited by light diffraction, > 1 μm
- **Data post-processing:** The z-coordinate acquired with the microscope together with the length of each side of the scanned field were extracted and saved in a Matlab® workspace without any processing. The data

were uploaded on the web app “contact engineering”, where we have indicated the reliability cutoff equal to 1 μm and we used the function “remove tilt”.

- **Sample preparation, if any:**

  Only the rougher samples, labelled Q32 and Q33, were measured. The experimental procedure is detailed as follows:

  a. The sample was blown with air to remove particle dust and was placed on the XYθ dark plate with plastic tweezers to avoid scratches.
  b. The microscope was set in reflection mode and adopted a fully coaxial illumination.
  c. For the 3-dimensional reconstruction the magnification 700x was used, corresponding to {L_x,L_y }={330 μm,440 μm} and vertical working distance from the sample equal to 6.3 mm.
  d. For the acquisition we randomly set a starting point on the sample and performed a 3-dimensional automatic sticking of about 300 μm width and 2000 μm length.
  e. For every sample 4 measurements were performed.

To increase the level of detail of the image we used a coaxial polarized light, the differential interference contrast (DIC) prism to increases the image contrast and performed the acquisition with the option “glare removal”. The acquisition time for each topographic scan was around 15 minutes.

AUTHOR AND TECHNIQUE INFORMATION FOR THE SURFACE-TOPOGRAPHY CHALLENGE

Submit to: SurfaceTopographyChallenge@gmail.com ***(***Deadline: 29 Feb 2024***)***

- We created this template to standardize the information we get from each group.
- Information submitted will be published *verbatim* in the Supplementary Information section.
- ***Please fill out all yellow-highlighted fields to the best of your ability.***

AUTHORSHIP INFORMATION (*Text only, please. We cannot accept citations in this document.)*

- **Author information (please ONLY include authors that directly contributed)**

    - **Author 1:** <Pauli, Matthias>
        - **ORCID ID:** 0009-0000-6987-0950
        - **Address, Line 1:** <Polytec GmbH>
        - **Address, Line 2:** <Polytecplatz 1-7, 76337, Waldbronn, Germany>

- **Funding information, if any** *(how it should appear in the acknowledgements)*
    - **Funder:** <Insert funding agency>; **Grant number:** <Insert grant number >
        - *(NOTE: For more than one funder, please copy/paste additional Funding lines)*

DESCRIPTION OF TECHNIQUES USED FOR DATA COLLECTION

(For reproducibility purposes, the publishing journal requires that we specify all details of each technique used. If you have any questions or concerns, contact SurfaceTopographyChallenge@gmail.com)

- **Number of techniques used:** <1>

TECHNIQUE 1: [NOTE: Please copy/paste this section and repeat for each technique used]

(*Text only, please. We cannot accept citations in this document.)*

- **Common name of technique:** <CSI (coherence scanning interferometry) >
- **Type of technique:** < white light interferometer >
    - [atomic force microscope; white light interferometer chromatic aberration; digital 3D optical microscope; confocal laser scanning microscope; holographic measurement; angle-resolved spectroscopy; scanning electron microscope; reconstruction from scanning electron microscope; stylus profilometer/tactile microscope; transmission electron microscope; triboindenter; other (specify)]
- **Instrument manufacturer:** <Polytec GmbH, Waldbronn, Germany>
- **Instrument model:** <TopMap Micro.View+>
- **Tip size (if known):** < N/A >
- **Expected maximum lateral resolution:** <Pixel resolution: 0.12µm; optical resolution: 0.58µm >
- **Data post-processing:** <We submitted raw data without any filtering and filtered like follows:

D43_50x_CSIs_250x250:
Linear Regression
S-Filter [0.5 µm, Robust]
Spike Removal [5x5, 10, RMS, Spikes]
Fill [15x15, Rectangular, Median]
Median [7x7, ±0 m]

D44_50x_CSIs_250x250:
Linear Regression
S-Filter [0.5 µm, Robust]
Spike Removal [5x5, 10, RMS, Spikes]
Fill [15x15, Rectangular, Median]
Median [7x7, ±0 m]

S73_50x_CSIr_250x250:
Linear Regression
S-Filter [1 µm]
Fill [15x15, Median]
Median [3x3, ±0 m]

AUTHOR AND TECHNIQUE INFORMATION FOR THE SURFACE-TOPOGRAPHY CHALLENGE

Submit to: SurfaceTopographyChallenge@gmail.com ***(***Deadline: 29 Feb 2024***)***

S74_50x_CSIr_250x250:
Linear Regression
S-Filter [1 µm]
Fill [15x15, Median]
Median [3x3, ±0 m]
>

- **Sample preparation, if any:** <Dust removal by air>

TECHNIQUE 2 (if applicable): [NOTE: Please copy/paste this section and repeat for each technique used]

(*Text only, please. We cannot accept citations in this document.)*

- **Common name of technique:** <Generic name; not brand names>
- **Type of technique:** <Choose one of the following categories>
    - [atomic force microscope; white light interferometer chromatic aberration; digital 3D optical microscope; confocal laser scanning microscope; holographic measurement; angle-resolved spectroscopy; scanning electron microscope; reconstruction from scanning electron microscope; stylus profilometer/tactile microscope; transmission electron microscope; triboindenter; other (specify)]
- **Instrument manufacturer:** <Name, city, state>
- **Instrument model:** <Brand name and model number>
- **Tip size (if known):** <Insert "N/A" for non-contact instruments>
- **Expected maximum lateral resolution:** <Based on manufacturer specs or technique info>
- **Data post-processing:** <Briefly describe any processing you did (such as tilt-correction or artifact removal) prior to submission of the data – either on the collection instrument or in other software.>
- **Sample preparation, if any:** <Briefly describe any sample preparation (cleaning, hot-mounting, etc.)>

AUTHOR AND TECHNIQUE INFORMATION FOR THE SURFACE-TOPOGRAPHY CHALLENGE

Submit to: SurfaceTopographyChallenge@gmail.com ***(***Deadline: 29 Feb 2024***)***

- We created this template to standardize the information we get from each group.
- Information submitted will be published *verbatim* in the Supplementary Information section.
- ***Please fill out all yellow-highlighted fields to the best of your ability.***

AUTHORSHIP INFORMATION (*Text only, please. We cannot accept citations in this document.)*

- **Author information (please ONLY include authors that directly contributed)**
    - **Author** Persson, Bo N. J.
        - **ORCID ID:** 0000-0003-1535-738X
        - **Address, Line 1:** Peter Grünberg Institute (PGI-1),
        - **Address, Line 2:** Forschungszentrum Jülich, 52425, Jülich, Germany
    - **Author 2:** Rodriguez, Nestor
        - **ORCID ID:** 0000-0002-6182-3428
        - **Address, Line 1:** BD Medical-Pharmaceutical Systems
        - **Address, Line 2:** 1 Becton Drive, Franklin Lakes, NY, 07417, USA
    - **Author 3:** Gontard, Lucile
        - **ORCID ID:** 0009-0006-6028-2785
        - **Address, Line 1:** BD Medical-Pharmaceutical Systems
        - **Address, Line 2:** 11 Rue Aristide-Verges, Le Pont de Claix 38801, France

- **Funding information, if any** *(how it should appear in the acknowledgements)*
    - **None**

DESCRIPTION OF TECHNIQUES USED FOR DATA COLLECTION

(For reproducibility purposes, the publishing journal requires that we specify all details of each technique used. If you have any questions or concerns, contact SurfaceTopographyChallenge@gmail.com)

- **Number of techniques used:** 6

TECHNIQUE 1: [NOTE: Please copy/paste this section and repeat for each technique used]

(*Text only, please. We cannot accept citations in this document.)*

- **Common name of technique:** Stylus
- **Type of technique:** Stylus Profilometer
- **Instrument manufacturer:** Bruker, Billerica, MA, USA
- **Instrument model:** Bruker Dektak XT
- **Tip Size (if known):** 0.7 µm radius (PN 838-030-3)
- **Contact Force:** 9.8E-3 mN (1 mgf)
- **Tip Velocity:** 30-44 µm/s
- **Expected scan vertical resolution:** 1Å max. (@ 6.55µm range)
- **Expected maximum lateral resolution:** 0.5 µm
- **Data post-processing:** None, all data processing algorithms disabled.
- **Sample preparation, if any:** Samples were cleaned liquid HPLC grade ethanol stream and let dry under clean laboratory environment before the measurement session.

AUTHOR AND TECHNIQUE INFORMATION FOR THE SURFACE-TOPOGRAPHY CHALLENGE

Submit to: SurfaceTopographyChallenge@gmail.com ***(***Deadline: 29 Feb 2024***)***

TECHNIQUE 2: [NOTE: Please copy/paste this section and repeat for each technique used]

(*Text only, please. We cannot accept citations in this document.)*

- **Common name of technique:** Stylus
- **Type of technique:** Stylus Profilometer
- **Instrument manufacturer:** Mitutoyo America Corporation, Aurora, IL, USA
- **Instrument model:** Surftest SJ-411
- **Tip size (if known):** 1.0 µm radius
- **Contact Force:** 0.75 mN
- **Tip Velocity:** 50 µm/s
- **Expected scan vertical resolution:** 0.01 µm
- **Expected maximum lateral resolution (straightness):** 18 nm/mm.
- **Data post-processing:** None, all data processing algorithms disabled.
- **Sample preparation, if any:** Samples were cleaned liquid distilled water stream and let dry under normal laboratory environment before the measurement session.

TECHNIQUE 3: [NOTE: Please copy/paste this section and repeat for each technique used]

(*Text only, please. We cannot accept citations in this document.)*

- **Common name of technique:** AFM
- **Type of technique:** Atomic force microscope, tapping mode
- **Instrument manufacturer:** Bruker, Billerica, MA, USA
- **Instrument model:** Dimension 3100
- **Tip size (if known):** 8 nm radius (RSTESPA-300)
- **Tip Velocity:** 6.00 µm/s
- **Expected maximum lateral resolution:** 20 nm
- **Data post-processing:** None, all data processing algorithms disabled.
- **Sample preparation, if any:** Samples were cleaned liquid HPLC grade ethanol stream and let dry under clean laboratory environment before the measurement session.

TECHNIQUE 4: [NOTE: Please copy/paste this section and repeat for each technique used]

(*Text only, please. We cannot accept citations in this document.)*

- **Common name of technique:** Optical Profilometer
- **Type of technique:** Green Light Interferometric Profilometer
- **Instrument manufacturer:** Bruker, Billerica, MA, USA
- **Instrument model:** Wyko NT9100
- **Green Light wavelength:** 535 nm
- **Magnification:** 50X
- **Field of View:** 125 µm x 93 µm (640 x 480 pixels)
- **Expected maximum vertical resolution:** 0.1 nm
- **Expected maximum lateral spatial resolution:** 0.1 µm
- **Data post-processing:** None, all data processing algorithms disabled.
- **Sample preparation, if any:** Samples were cleaned liquid HPLC grade ethanol stream and let dry under clean laboratory environment before the measurement session.

AUTHOR AND TECHNIQUE INFORMATION FOR THE SURFACE-TOPOGRAPHY CHALLENGE

Submit to: SurfaceTopographyChallenge@gmail.com ***(***Deadline: 29 Feb 2024***)***

TECHNIQUE 5: [NOTE: Please copy/paste this section and repeat for each technique used]

(*Text only, please. We cannot accept citations in this document.)*

- **Common name of technique:** Laser Profilometer
- **Type of technique:** Confocal Laser Profilometer.
- **Instrument manufacturer:** Keyence, MA, USA
- **Instrument model:** VK-X105
- **Light wavelength:** 658 nm
- **Magnification:** 10X to 100 X
- **Field of View:** 1.35 x 1.012 to 0.135 x 0.101 mm^{2mm}
- **Expected maximum heigh resolution:** 5 nm
- **Expected maximum lateral/width resolution:** 20 nm
- **Data post-processing:** None, all data processing algorithms disabled.
- **Sample preparation, if any:** Samples were cleaned liquid HPLC grade ethanol stream and let dry under clean laboratory environment before the measurement session.

TECHNIQUE 6: [NOTE: Please copy/paste this section and repeat for each technique used]

(*Text only, please. We cannot accept citations in this document.)*

- **Common name of technique:** White Light Profilometer
- **Type of technique:** White Light Confocal Profilometer.
- **Instrument manufacturer:** Keyence, MA, USA
- **Instrument model:** VR 5200
- **Magnification:** 12 to 160X
- **Field of View**: 24 x 18 to 1.9 x 1.4 mm^2
- **Expected maximum heigh resolution (without stitching):** 0.4 µm
- **Expected maximum lateral/width resolution:** 2.0 µm
- **Data post-processing:** None, all data processing algorithms disabled.
- **Sample preparation, if any:** Samples were cleaned liquid HPLC grade ethanol stream and let dry under clean laboratory environment before the measurement session.

AUTHOR AND TECHNIQUE INFORMATION FOR THE SURFACE-TOPOGRAPHY CHALLENGE

Submit to: SurfaceTopographyChallenge@gmail.com ***(***Deadline: 29 Feb 2024***)***

- We created this template to standardize the information we get from each group.
- Information submitted will be published *verbatim* in the Supplementary Information section.
- ***Please fill out all yellow-highlighted fields to the best of your ability.***

AUTHORSHIP INFORMATION (*Text only, please. We cannot accept citations in this document.)*

- **Author information (please ONLY include authors that directly contributed)**
  - **Author 1:** Peterson, Amy
    - **ORCID ID:** 0000-0002-4612-0062
    - **Address, Line 1:** Department of Plastics Engineering, University of Massachusetts Lowell
    - **Address, Line 2:** 1 University Ave., Ball 213, Lowell, MA, 01854, USA
  - **Author 2:** Tripathi, Sandeep
    - **ORCID ID:** 0009-0007-1799-6923
    - **Address, Line 1:** Department of Plastics Engineering, University of Massachusetts Lowell
    - **Address, Line 2:** 40 University Ave., ETIC 215, Lowell, MA, 01854, USA

- **Funding information, if any** *(how it should appear in the acknowledgements)*
  - **Funder:** N/A

DESCRIPTION OF TECHNIQUES USED FOR DATA COLLECTION

(For reproducibility purposes, the publishing journal requires that we specify all details of each technique used. If you have any questions or concerns, contact SurfaceTopographyChallenge@gmail.com)

- **Number of techniques used:** 1

TECHNIQUE 1: [NOTE: Please copy/paste this section and repeat for each technique used]

(*Text only, please. We cannot accept citations in this document.)*

- **Common name of technique:** AFM
- **Type of technique:** atomic force microscopy, contact mode
- **Instrument manufacturer:** Park systems, Santa Clara, CA, USA
- **Instrument model:** XE-100
- **Tip size (if known):** 8 nm
- **Expected maximum lateral resolution:** 117 nm
- **Data post-processing:** NA
- **Sample preparation, if any:** NA

AUTHOR AND TECHNIQUE INFORMATION FOR THE SURFACE-TOPOGRAPHY CHALLENGE

Submit to: SurfaceTopographyChallenge@gmail.com ***(***Deadline: 29 Feb 2024***)***

AUTHORSHIP INFORMATION

- **Author information (please ONLY include authors that directly contributed)**
  - **Author 1:** Lee, Peter M.
    - **ORCID ID:** 0000-0001-5582-1998
    - **Address, Line 1:** Southwest Research Institute
    - **Address, Line 2:** 6220 Culebra Rd, San Antonio, Texas, 78238, United States
  - **Author 2:** Reyes, Isaiah J.
    - **ORCID ID:** 0009-0008-0666-4664
    - **Address, Line 1:** Southwest Research Institute
    - **Address, Line 2:** 6220 Culebra Rd, San Antonio, Texas, 78238, United States

- **Funding information, if any**
  - **Funder:** Southwest Research Institute; **Grant number:** 1.08.01

DESCRIPTION OF TECHNIQUES USED FOR DATA COLLECTION

- **Number of techniques used:** 2

TECHNIQUE 1: [NOTE: Please copy/paste this section and repeat for each technique used]

- **Common name of technique:** Optical
- **Type of technique:** Digital 3D Optical Microscope
- **Instrument manufacturer:** Keyence, Osaka, Japan
- **Instrument model:** Keyence VR-5200
- **Tip size (if known):** N/A
- **Expected maximum lateral resolution:** 1mm
- **Data post-processing:** Tilt Correction
- **Sample preparation, if any:** N/A

TECHNIQUE 2 (if applicable): [NOTE: Please copy/paste this section and repeat for each technique used]

- **Common name of technique:** Optical
- **Type of technique:** White Light Interferometer
- **Instrument manufacturer:** Bruker, Billerica, MA
- **Instrument model:** Bruker NP Flex 3D Surface Meteorology System
- **Tip size (if known):** N/A
- **Expected maximum lateral resolution:** <15nm
- **Data post-processing:** N/A
- **Sample preparation, if any:** N/A

AUTHOR AND TECHNIQUE INFORMATION FOR THE SURFACE-TOPOGRAPHY CHALLENGE

Submit to: SurfaceTopographyChallenge@gmail.com ***(***Deadline: 29 Feb 2024***)***

- We created this template to standardize the information we get from each group.
- Information submitted will be published *verbatim* in the Supplementary Information section.
- ***Please fill out all yellow-highlighted fields to the best of your ability.***

AUTHORSHIP INFORMATION (*Text only, please. We cannot accept citations in this document.)*

- **Author information (please ONLY include authors that directly contributed)**
    - **Author 1:** Prieto, G.
        - **ORCID ID:** 0000-0002-8673-0375
        - **Address, Line 1:** IFISUR, Universidad Nacional del Sur/CONICET
        - **Address, Line 2:** Av. Alem 1253, Bahía Blanca, Buenos Aires, CP 8000, Argentina.
    - **Author 2:** Abdelnabe, J. P.
        - **ORCID ID:** 0009-0008-8077-2196
        - **Address, Line 1:** IFISUR, Universidad Nacional del Sur/CONICET
        - **Address, Line 2:** Av. Alem 1253, Bahía Blanca, Buenos Aires, CP 8000, Argentina.
    - **Author 3:** Tuckart, W. R.
        - **ORCID ID:** -
        - **Address, Line 1:** IFISUR, Universidad Nacional del Sur/CONICET
        - **Address, Line 2:** Av. Alem 1253, Bahía Blanca, Buenos Aires, CP 8000, Argentina.

- **Funding information, if any** *(how it should appear in the acknowledgements)*
    - **Funder:** Agencia Nacional de Promoción Científica y Tecnológica – ANPCyT – Argentina; **Grant number:** PICT-2021-I-A-00458

DESCRIPTION OF TECHNIQUES USED FOR DATA COLLECTION

(For reproducibility purposes, the publishing journal requires that we specify all details of each technique used. If you have any questions or concerns, contact SurfaceTopographyChallenge@gmail.com)

- **Number of techniques used:** 1.

TECHNIQUE 1: [NOTE: Please copy/paste this section and repeat for each technique used]

(*Text only, please. We cannot accept citations in this document.)*

- **Common name of technique:** Laser confocal microscopy.
- **Type of technique:** confocal laser scanning microscope.
    - [atomic force microscope; white light interferometer chromatic aberration; digital 3D optical microscope; confocal laser scanning microscope; holographic measurement; angle-resolved spectroscopy; scanning electron microscope; reconstruction from scanning electron microscope; stylus profilometer/tactile microscope; transmission electron microscope; triboindenter; other (specify)]
- **Instrument manufacturer:** Carl Zeiss, Jena, Thuringia.
- **Instrument model:** Smartproof 5.
- **Tip size (if known):** N/A.
- **Expected maximum lateral resolution:** 0.13 µm.
- **Data post-processing:** None.
- **Sample preparation, if any:** Ultrasonic cleaning with acetone during 10 minutes.

AUTHOR AND TECHNIQUE INFORMATION FOR THE SURFACE-TOPOGRAPHY CHALLENGE

Submit to: SurfaceTopographyChallenge@gmail.com ***(***Deadline: 29 Feb 2024***)***

- We created this template to standardize the information we get from each group.
- Information submitted will be published *verbatim* in the Supplementary Information section.
- ***Please fill out all yellow-highlighted fields to the best of your ability.***

AUTHORSHIP INFORMATION (*Text only, please. We cannot accept citations in this document.)*

- **Author information (please ONLY include authors that directly contributed)**
  - **Author 1:** <Afferrante, L.>
    - **ORCID ID:** <0000-0003-2745-1453>
    - **Address, Line 1:** <Smart Tribology Lab, Department of Mechanics, Mathematics and Management, Polytechnic University of Bari>
    - **Address, Line 2:** <Via E. Orabona 4, 70125 Bari, Italy>
  - **Author 2:** <Violano, G.>
    - **ORCID ID:** <0000-0002-9092-4930>
    - **Address, Line 1:** < Smart Tribology Lab, Department of Mechanics, Mathematics and Management, Polytechnic University of Bari>
    - **Address, Line 2:** <Via E. Orabona 4, 70125 Bari, Italy>
  - **Author 3:** <Putignano, C.>
    - **ORCID ID:** <0000-0001-6225-9630>
    - **Address, Line 1:** < Smart Tribology Lab, Department of Mechanics, Mathematics and Management, Polytechnic University of Bari>
    - **Address, Line 2:** <Via E. Orabona 4, 70125 Bari, Italy>
  - **Author 4:** <Bottiglione, F.>
    - **ORCID ID:** <0000-0002-0953-352X>
    - **Address, Line 1:** < Smart Tribology Lab, Department of Mechanics, Mathematics and Management, Polytechnic University of Bari>
    - **Address, Line 2:** <Via E. Orabona 4, 70125 Bari, Italy>
  - **Author 5:** <Menga, N.>
    - **ORCID ID:** <0000-0002-4728-1773 >
    - **Address, Line 1:** < Smart Tribology Lab, Department of Mechanics, Mathematics and Management, Polytechnic University of Bari>
    - **Address, Line 2:** <Via E. Orabona 4, 70125 Bari, Italy>
  - **Author 6:** <Carbone, G.>
    - **ORCID ID:** <0000-0002-8919-6796>
    - **Address, Line 1:** < Smart Tribology Lab, Department of Mechanics, Mathematics and Management, Polytechnic University of Bari>
    - **Address, Line 2:** <Via E. Orabona 4, 70125 Bari, Italy>

- **Funding information, if any** *(how it should appear in the acknowledgements)*
  - **Funder:** <Italian Ministry of University and Research under the Programme "Department of Excellence" L. 232/2016> **Grant number:** <CUP - D93C23000100001>

DESCRIPTION OF TECHNIQUES USED FOR DATA COLLECTION

(For reproducibility purposes, the publishing journal requires that we specify all details of each technique used. If you have any questions or concerns, contact SurfaceTopographyChallenge@gmail.com)

- **Number of techniques used:** <2>

TECHNIQUE 1: [NOTE: Please copy/paste this section and repeat for each technique used]

(*Text only, please. We cannot accept citations in this document.)*

- **Common name of technique:** <Atomic Force Microscopy (AFM)>
- **Type of technique:** <atomic force microscope>
  - [atomic force microscope; white light interferometer chromatic aberration; digital 3D optical microscope; confocal laser scanning microscope; holographic measurement; angle-resolved spectroscopy; scanning electron microscope; reconstruction from scanning electron microscope; stylus profilometer/tactile microscope; transmission electron microscope; triboindenter; other (specify)]

AUTHOR AND TECHNIQUE INFORMATION FOR THE SURFACE-TOPOGRAPHY CHALLENGE

Submit to: SurfaceTopographyChallenge@gmail.com ***(***Deadline: 29 Feb 2024***)***

- **Instrument manufacturer:** < NT-MDT Co., Sutton 11A, 7327 AB Apeldoorn, The Netherlands>
- **Instrument model:** <NT-MDT NTEGRA Prima>>
- **Tip size (if known):** <2 nm (guaranteed < 5 nm)>
- **Expected maximum lateral resolution:** <~10 nm>
- **Data post-processing:** <The raw data were post-processed with the following steps:
    - Tilt compensation: This step was carried out to remove the overall inclination of the surface.
    - Windowing of the surface: We applied a Hann window to the surface to reduce the effects of spectral leakage.
    - Oversampling: An oversampling factor of two was utilized to reduce aliasing.>
- **Sample preparation, if any:** <The samples were cleaned before each measurement. The cleaning procedure involved a 60-second isopropanol bath, followed by approximately 5 minutes air drying in a fume hood.>

TECHNIQUE 2 (if applicable): [NOTE: Please copy/paste this section and repeat for each technique used]

(*Text only, please. We cannot accept citations in this document.)*

- **Common name of technique:** <Confocal Microscopy>
- **Type of technique:** <confocal laser scanning microscope>
    - [atomic force microscope; white light interferometer chromatic aberration; digital 3D optical microscope; confocal laser scanning microscope; holographic measurement; angle-resolved spectroscopy; scanning electron microscope; reconstruction from scanning electron microscope; stylus profilometer/tactile microscope; transmission electron microscope; triboindenter; other (specify)]
- **Instrument manufacturer:** <CSM Instruments-SA Rue de la Gare 4 CH-2034 Peseux Switzerland>
- **Instrument model:** < CSM ConScan CL1>
- **Tip size (if known):** <N/A>
- **Expected maximum lateral resolution:** <1 micron>
- **Data post-processing:** <The raw data were post-processed with the following steps:
    - Tilt compensation: This step was carried out to remove the overall inclination of the surface.
    - Windowing of the surface: We applied a Hann window to the surface to reduce the effects of spectral leakage.
    - Oversampling: An oversampling factor of two was utilized to reduce aliasing.>
- **Sample preparation, if any:** <The samples were cleaned before each measurement. The cleaning procedure involved a 60-second isopropanol bath, followed by approximately 5 minutes air drying in a fume hood.>

AUTHOR AND TECHNIQUE INFORMATION FOR THE SURFACE-TOPOGRAPHY CHALLENGE

Submit to: SurfaceTopographyChallenge@gmail.com ***(***Deadline: 29 Feb 2024***)***

- We created this template to standardize the information we get from each group.
- Information submitted will be published *verbatim* in the Supplementary Information section.
- ***Please fill out all yellow-highlighted fields to the best of your ability.***

AUTHORSHIP INFORMATION (*Text only, please. We cannot accept citations in this document.)*

- **Author information (please ONLY include authors that directly contributed)**
  - **Author 1:** Ramisetti, Srinivasa B.
    - **ORCID ID:** 0000-0002-2927-5257
    - **Address, Line 1:** Independent Researcher
    - **Address, Line 2:** Chennai, TN, 600041, India
  - **Author 2:** Yadav, Anshul
    - **ORCID ID:** 0000-0003-1380-2662
    - **Address, Line 1:** CSIR-Central Salt & Marine Chemicals Research Institute
    - **Address, Line 2:** Bhavnagar, Gujrat, 364002, India
  - *(NOTE: For three or more authors, please copy/paste additional Author lines)*

- **Funding information, if any** *(how it should appear in the acknowledgements)*
  - **Funder:** ; **Grant number:**
    - *(NOTE: For more than one funder, please copy/paste additional Funding lines)*

DESCRIPTION OF TECHNIQUES USED FOR DATA COLLECTION

(For reproducibility purposes, the publishing journal requires that we specify all details of each technique used. If you have any questions or concerns, contact SurfaceTopographyChallenge@gmail.com)

- **Number of techniques used: 1**

TECHNIQUE 1: [NOTE: Please copy/paste this section and repeat for each technique used]

(*Text only, please. We cannot accept citations in this document.)*

- **Common name of technique: AFM**
- **Type of technique:** atomic force microscope
- **Instrument manufacturer:** NT-MDT Spectrum Instruments, Ireland
- **Instrument model:** NTEGRA PRIMA
- **Tip size (if known):** 6 nm radius
- **Expected maximum lateral resolution:** 30 nm
- **Data post-processing:** Tilt correction with a 3rd order polynomial is applied
- **Sample preparation, if any:** No cleaning or other processing of the samples were carried out

AUTHOR AND TECHNIQUE INFORMATION FOR THE SURFACE-TOPOGRAPHY CHALLENGE

Submit to: SurfaceTopographyChallenge@gmail.com ***(***Deadline: 29 Feb 2024***)***

- We created this template to standardize the information we get from each group.
- Information submitted will be published *verbatim* in the Supplementary Information section.
- ***Please fill out all yellow-highlighted fields to the best of your ability.***

AUTHORSHIP INFORMATION (*Text only, please. We cannot accept citations in this document.)*

- **Author information (please ONLY include authors that directly contributed)**
  - **Author 1:** Raumel, Selina
    - **ORCID ID:** https://orcid.org/0000-0003-0666-2817
    - **Address, Line 1:** Institute for Micro Production Technology, Leibniz University Hanover
    - **Address, Line 2:** An der Universität 2, Garbsen, Lower Saxony, 30823, Germany
  - **Author 2:** Steinhoff, Lukas
    - **ORCID ID:** https://orcid.org/0000-0003-3411-5802
    - **Address, Line 1:** Institute for Micro Production Technology, Leibniz University Hanover
    - **Address, Line 2:** An der Universität 2, Garbsen, Lower Saxony, 30823, Germany

- **Funding information, if any** *(how it should appear in the acknowledgements)*
  - **Funder:** <Insert funding agency>; **Grant number:** <Insert grant number >
    - *(NOTE: For more than one funder, please copy/paste additional Funding lines)*

DESCRIPTION OF TECHNIQUES USED FOR DATA COLLECTION

(For reproducibility purposes, the publishing journal requires that we specify all details of each technique used. If you have any questions or concerns, contact SurfaceTopographyChallenge@gmail.com)

- **Number of techniques used:** 5

TECHNIQUE 1:

- **Common name of technique:** Confocal Laser Scanning Microscopy
- **Type of technique:** confocal laser scanning microscope
- **Instrument manufacturer:** Keyence, Osaka, Japan
- **Instrument model:** VK9700
- **Tip size (if known):** N/A
- **Expected maximum lateral resolution:** 0.001 µm
- **Data post-processing:** Besides submitting raw data, we did firstly tilt correction and then chose cut-off filters λc and λs according to ISO 4288-1997 to acquire the anlyse reports.
- **Sample preparation, if any:** Cleaned with pressurized air

---

TECHNIQUE 2.

- **Common name of technique:** Scanning Probe Microscopy
- **Type of technique:** triboindenter
- **Instrument manufacturer:** Bruker, Billerica, Massachusetts, USA
- **Instrument model:** Hysitron TI 900
- **Tip size (if known):** 50 nm
- **Expected maximum lateral resolution:** 50 nm
- **Data post-processing:** with and without linear regression
- **Sample preparation, if any:** Cleaned with pressurized air, magnetic mounting

---

TECHNIQUE 3:

- **Common name of technique:** Stylus
- **Type of technique:** Stylus profilometer

AUTHOR AND TECHNIQUE INFORMATION FOR THE SURFACE-TOPOGRAPHY CHALLENGE

Submit to: SurfaceTopographyChallenge@gmail.com ***(***Deadline: 29 Feb 2024***)***

- **Instrument manufacturer:** Bruker, Billerica, Massachusetts, USA
- **Instrument model:** DektakXT
- **Tip size (if known):** 2 µm
- **Expected maximum lateral resolution:** 2 µm
- **Data post-processing:** --
- **Sample preparation, if any:** Cleaned with pressurized air

---

TECHNIQUE 4:

- **Common name of technique:** White light microscopy
- **Type of technique:** white light interferometer
- **Instrument manufacturer:** Bruker (Wyko), Billerica, Massachusetts, USA
- **Instrument model:** RST+
- **Tip size (if known):** N/A
- **Expected maximum lateral resolution:** 1.5 µm
- **Data post-processing:** Besides submitting raw data, we did firstly tilt correction and then chose cut-off filters λc and λs according to ISO 4288-1997 to acquire the anlyse reports.
- **Sample preparation, if any:** Cleaned with pressurized air

---

TECHNIQUE 5:

- **Common name of technique:** Atomic force microscopy
- **Type of technique:** atomic force microscope
- **Instrument manufacturer:** Oxford Instruments, Abington, England
- **Instrument model:** Jupiter XR
- **Tip size (if known):** 7 nm (AC160)
- **Expected maximum lateral resolution:** 7 nm
- **Data post-processing:** chose cut-off filters λc according to ISO 4288-1997
- **Sample preparation, if any:** None

AUTHOR AND TECHNIQUE INFORMATION FOR THE SURFACE-TOPOGRAPHY CHALLENGE

Submit to: SurfaceTopographyChallenge@gmail.com ***(***Deadline: 29 Feb 2024***)***

- We created this template to standardize the information we get from each group.
- Information submitted will be published *verbatim* in the Supplementary Information section.
- ***Please fill out all yellow-highlighted fields to the best of your ability.***

AUTHORSHIP INFORMATION (*Text only, please. We cannot accept citations in this document.*)

- **Author information (please ONLY include authors that directly contributed)**
  - **Author 1:** Rojacz, Harald
    - **ORCID ID:** 0000-0003-1810-0854
    - **Address, Line 1:** AC2T research GmbH
    - **Address, Line 2:** Viktor-Kaplan-Strasse 2/C, Wiener Neustadt, 2700, Austria
  - **Author 2:** Vorlaufer, Georg
    - **ORCID ID:** 0000-0003-0707-4697
    - **Address, Line 1:** AC2T research GmbH
    - **Address, Line 2:** Viktor-Kaplan-Strasse 2/C, Wiener Neustadt, 2700, Austria
  - **Author 3:** Rodríguez Ripoll, Manel
    - **ORCID ID:** 0000-0001-9024-9587
    - **Address, Line 1:** AC2T research GmbH
    - **Address, Line 2:** Viktor-Kaplan-Strasse 2/C, Wiener Neustadt, 2700, Austria
  - *(NOTE: For three or more authors, please copy/paste additional Author lines)*

- **Funding information, if any** *(how it should appear in the acknowledgements)*
  - **Funder:** Austrian COMET-Programme, Project K2 InTribology2; **Grant number:** 906860
    - *(NOTE: For more than one funder, please copy/paste additional Funding lines)*

DESCRIPTION OF TECHNIQUES USED FOR DATA COLLECTION

(For reproducibility purposes, the publishing journal requires that we specify all details of each technique used. If you have any questions or concerns, contact SurfaceTopographyChallenge@gmail.com)

- **Number of techniques used:** 5

TECHNIQUE 1: [NOTE: Please copy/paste this section and repeat for each technique used]

(*Text only, please. We cannot accept citations in this document.*)

- **Common name of technique:** Focus variation
- **Type of technique:** digital 3D optical microscope
- **Instrument manufacturer:** Bruker Alicona, Raaba/Graz, Austria
- **Instrument model:** InfiniteFocus G5
- **Tip size (if known):** N/A
- **Expected maximum lateral resolution:** 880 nm (20x objective) and 640 nm (50x objective)
- **Data post-processing:** No data post-processing done
- **Sample preparation, if any:** The samples were cleaned using isoproylic alcohol and subsequent drying in an air flow. No swabs, cotton balls or other mechanical cleaning was utilised for cleaning.

TECHNIQUE 2: [NOTE: Please copy/paste this section and repeat for each technique used]

(*Text only, please. We cannot accept citations in this document.*)

- **Common name of technique:** Confocal
- **Type of technique:** confocal laser LED microscope
- **Instrument manufacturer:** Leica Microsystems GmbH, Wetzlar, Germany
- **Instrument model:** Leica DCM 8
- **Tip size (if known):** N/A
- **Expected maximum lateral resolution:** 280 nm (20x objective), 160 nm (50 objective) and 140 nm (100x objective)
- **Data post-processing:** No data post-processing done
- **Sample preparation, if any:** The samples were cleaned using isoproylic alcohol and subsequent drying in an air flow. No swabs, cotton balls or other mechanical cleaning was utilised for cleaning.

TECHNIQUE 3: [NOTE: Please copy/paste this section and repeat for each technique used]

(*Text only, please. We cannot accept citations in this document.*)

AUTHOR AND TECHNIQUE INFORMATION FOR THE SURFACE-TOPOGRAPHY CHALLENGE

Submit to: SurfaceTopographyChallenge@gmail.com ***(***Deadline: 29 Feb 2024***)***

- **Common name of technique:** White light interferometry
- **Type of technique:** white light interferometer chromatic aberration
- **Instrument manufacturer:** Leica Microsystems GmbH, Wetzlar, Germany
- **Instrument model:** Leica DCM 8
- **Tip size (if known):** N/A
- **Expected maximum lateral resolution:** 250 nm (50x objective)
- **Data post-processing:** No data post-processing done
- **Sample preparation, if any:** The samples were cleaned using isoproylic alcohol and subsequent drying in an air flow. No swabs, cotton balls or other mechanical cleaning was utilised for cleaning.

TECHNIQUE 4: [NOTE: Please copy/paste this section and repeat for each technique used]

(*Text only, please. We cannot accept citations in this document.)*

- **Common name of technique:** Triboindenter
- **Type of technique:** Triboindenter
- **Instrument manufacturer:** Bruker Corporation, MA, USA
- **Instrument model:** Hysitron Triboindenter TI 950 (PerforMech II transducer)
- **Tip size (if known):** Berkovich diamond indenter with a tip radius of 40 nm
- **Expected maximum lateral resolution:** 50 nm
- **Data post-processing:** No data post-processing done
- **Sample preparation, if any:** The samples were cleaned using isoproylic alcohol and subsequent drying in an air flow. No swabs, cotton balls or other mechanical cleaning was utilised for cleaning.

TECHNIQUE 5: [NOTE: Please copy/paste this section and repeat for each technique used]

(*Text only, please. We cannot accept citations in this document.)*

- **Common name of technique:** Scanning electron microscope
- **Type of technique:** Scanning electron microscope
- **Instrument manufacturer:** JEOL Ltd., Tokyo, Japan
- **Instrument model:** Jeol JIB-4700 F
- **Tip size (if known):** N/A
- **Expected maximum lateral resolution:** 1.2 nm
- **Data post-processing:** The FIB cuts were analyzed using standard image processing routines (in this case via the open source package "OpenCV" and the programming language "Python"). In a first step, the region around an individual FIB cut was selected manually. The edge was detected using a threshold on the grey level of the SEM image, where the threshold was automatically determined from the intensity distribution (using the local minimum of the bimodal distribution – dark and light areas). Then, the pixels representing the edge were used to calculate the line profile parameters Ra, Rq and Rz. The pixel dimensions were scaled according to the scale bar in the images and the aspect ratio was corrected according to the view angle (53° angle between cut plane and optical axis).
- **Sample preparation, if any:** Three focused ion beam (FIB) cuts of 200 µm length, 15 µm width and a depth of ~20 µm were prepared. Prior to cutting a masking with 0.4 µm carbon was applied, to prevent curtaining and for better contrasting.

AUTHOR AND TECHNIQUE INFORMATION FOR THE SURFACE-TOPOGRAPHY CHALLENGE

Submit to: SurfaceTopographyChallenge@gmail.com ***(***Deadline: 29 Feb 2024***)***

- We created this template to standardize the information we get from each group.
- Information submitted will be published *verbatim* in the Supplementary Information section.
- ***Please fill out all yellow-highlighted fields to the best of your ability.***

AUTHORSHIP INFORMATION

- **Author information**
  - **Author 1:** Scaraggi, Michele
    - **ORCID ID:** 0000-0001-8964-9970
    - **Address, Line 1:** Department of Engineering for Innovation, University of Salento
    - **Address, Line 2:** Centro Ecotekne Pal. O - S.P. 6, 73100 Monteroni-Lecce, Italy
    - **Address, Line 1BIS:** Center for Biomolecular Nanotechnologies, Istituto Italiano di Tecnologia
    - **Address, Line 2BIS:** Via Barsanti 14, Arnesano 73010, Italy
  - **Author 2:** Algieri, Luciana
    - **ORCID ID:** 0000-0003-3667-5432
    - **Address, Line 1:** Department of Engineering for Innovation, University of Salento
    - **Address, Line 2:** Centro Ecotekne Pal. O - S.P. 6, 73100 Monteroni-Lecce, Italy

- **Funding information**
  - **Funder:** MUR - Italian Minster of University and Research; **Grant number:** P2022MAZHX, under research project PRIN2022 PNRR TRIBOSCORE.

DESCRIPTION OF TECHNIQUES USED FOR DATA COLLECTION

- **Number of techniques used:** 2

TECHNIQUE 1:

- **Common name of technique:** AFM
- **Type of technique**:_Atomic force microscope, contact mode
- **Instrument manufacturer**: CSI – Concept Scientific Instruments, Les ULIS, France
- **Instrument model:** CSI-Nano-Observer AFM
- **Tip size (if known):** Two different probes were used, with radius of curvature 6 nm
- **Expected maximum lateral resolution:** Two different lateral resolutions were used, respectively 5/511 µm, 20/511 µm
- **Data post-processing:** No
- **Sample preparation, if any:** Isopropanol followed by N_2 gas flow just before starting the measurement

---

TECHNIQUE 2:

- **Common name of technique:** Stylus
- **Type of technique:**_Stylus profilometer
- **Instrument manufacturer:** Bruker, Billerica, MA, USA
- **Instrument model:** Bruker Dektak XT profilometer
- **Tip size (if known):** Radius of curvature 2µm
- **Expected maximum lateral resolution:** 1.00 µm
- **Data post-processing:** No
- **Sample preparation, if any:** Isopropanol followed by N2 gas flow just before starting the measurement

AUTHOR AND TECHNIQUE INFORMATION FOR THE SURFACE-TOPOGRAPHY CHALLENGE

Submit to: SurfaceTopographyChallenge@gmail.com ***(***Deadline: 29 Feb 2024***)***

- We created this template to standardize the information we get from each group.
- Information submitted will be published *verbatim* in the Supplementary Information section.
- ***Please fill out all yellow-highlighted fields to the best of your ability.***

AUTHORSHIP INFORMATION (*Text only, please. We cannot accept citations in this document.*)

- **Author information (please ONLY include authors that directly contributed)**
  - **Author 1:** Shaffer, Kathryn E.
    - **ORCID ID:** 0009-0004-6840-4295
    - **Address, Line 1:** Materials Department, University of California, Santa Barbara
    - **Address, Line 2:** Materials Research Laboratory, Santa Barbara, CA, 93106, USA
  - **Author 2:** Pitenis, Angela A.
    - **ORCID ID:** 0000-0002-9697-7291
    - **Address, Line 1:** Materials Department, University of California, Santa Barbara
    - **Address, Line 2:** Materials Research Laboratory, Santa Barbara, CA, 93106, USA

- **Funding information, if any** *(how it should appear in the acknowledgements)*
  - **Funder:** Department of Energy; **Grant number:** DE-SC0024149

DESCRIPTION OF TECHNIQUES USED FOR DATA COLLECTION

(For reproducibility purposes, the publishing journal requires that we specify all details of each technique used. If you have any questions or concerns, contact SurfaceTopographyChallenge@gmail.com)

- **Number of techniques used:** 3

TECHNIQUE 1: [NOTE: Please copy/paste this section and repeat for each technique used]

(*Text only, please. We cannot accept citations in this document.*)

- **Common name of technique:** Scanning electron microscopy
- **Type of technique:** Scanning electron microscope
- **Instrument manufacturer:** FEI (Thermo Fisher Scientific), Hillsboro, Oregon
- **Instrument model:** Nova Nano 650 FEG SEM
- **Tip size (if known):** N/A
- **Expected maximum lateral resolution:** Unknown due to multiple filters, likely 10s of nm.
- **Data post-processing:** ImageJ was used to convert cross sections of specimen edges into binary images, of which the boundry lines were extracted as roughness profiles.
- **Sample preparation, if any:** Sample was rinsed with DI water and dish soap was massaged onto sample with gloved hands. The sample was then rinsed in DI water, then acetone, then isopropanol. The sample was then sonicated in methanol for 30 min and dried with nitrogen.

TECHNIQUE 2 (if applicable): [NOTE: Please copy/paste this section and repeat for each technique used]

(*Text only, please. We cannot accept citations in this document.*)

- **Common name of technique:** Atomic force microscopy
- **Type of technique:** Atomic force microscope
- **Instrument manufacturer:** Asylum Instruments, Santa Barbara, California
- **Instrument model:** MFP3D-Bio
- **Tip size (if known):** 7 nm radius
- **Expected maximum lateral resolution:** < 7nm
- **Data post-processing:** images save with a tilt correction
- **Sample preparation, if any:** Sample was rinsed with DI water and dish soap was massaged onto sample with gloved hands. The sample was then rinsed in DI water, then acetone, then isopropanol. The sample was then sonicated in methanol for 30 min and dried with nitrogen

AUTHOR AND TECHNIQUE INFORMATION FOR THE SURFACE-TOPOGRAPHY CHALLENGE

Submit to: SurfaceTopographyChallenge@gmail.com ***(***Deadline: 29 Feb 2024***)***

TECHNIQUE 3: [NOTE: Please copy/paste this section and repeat for each technique used]

(*Text only, please. We cannot accept citations in this document.)*

- **Common name of technique:** Laser scanning confocal microscopy
- **Type of technique:** Confocal laser scanning microscope
- **Instrument manufacturer:** Olympus, Center Valley, PA
- **Instrument model:** LEXT OLS4000
- **Tip size (if known):** N/A
- **Expected maximum lateral resolution:** 10 nm
- **Data post-processing:** N/A
- **Sample preparation, if any:** Sample was rinsed with DI water and dish soap was massaged onto sample with gloved hands. The sample was then rinsed in DI water, then acetone, then isopropanol. The sample was then sonicated in methanol for 30 min and dried with nitrogen

AUTHOR AND TECHNIQUE INFORMATION FOR THE SURFACE-TOPOGRAPHY CHALLENGE

Submit to: SurfaceTopographyChallenge@gmail.com ***(***Deadline: 29 Feb 2024***)***

- We created this template to standardize the information we get from each group.
- Information submitted will be published *verbatim* in the Supplementary Information section.
- ***Please fill out all yellow-highlighted fields to the best of your ability.***

AUTHORSHIP INFORMATION (*Text only, please. We cannot accept citations in this document.)*

- **Author information (please ONLY include authors that directly contributed)**
  - **Author 1:** Scherrer, Simon W.
    - **ORCID ID:** 0000-0001-5479-8765
    - **Address, Line 1:** Department of Materials, ETH Zürich
    - **Address, Line 2:** Leopold-Ruzicka-Weg 4, Zürich, Zürich, 8093, Switzerland
  - **Author 2:** Isa, Lucio
    - **ORCID ID:** 0000-0001-6731-9620
    - **Address, Line 1:** Department of Materials, ETH Zürich
    - **Address, Line 2:** Leopold-Ruzicka-Weg 4, Zürich, Zürich, 8093, Switzerland
  - *(NOTE: For three or more authors, please copy/paste additional Author lines)*

- **Funding information, if any** *(how it should appear in the acknowledgements)*
  - **Funder:** <Insert funding agency>; **Grant number:** <Insert grant number >
    - *(NOTE: For more than one funder, please copy/paste additional Funding lines)*

DESCRIPTION OF TECHNIQUES USED FOR DATA COLLECTION

(For reproducibility purposes, the publishing journal requires that we specify all details of each technique used. If you have any questions or concerns, contact SurfaceTopographyChallenge@gmail.com)

- **Number of techniques used:** 2

TECHNIQUE 1: [NOTE: Please copy/paste this section and repeat for each technique used]

(*Text only, please. We cannot accept citations in this document.)*

- **Common name of technique:** AFM (Atomic Force Microscopy) in tapping mode
- **Type of technique: atomic force microscope**
- **Instrument manufacturer:** Bruker, Billerica, MA, USA
- **Instrument model:** Bruker Dimension Icon
- **Tip size (if known):** 7 nm radius
- **Expected maximum lateral resolution:** 0.1 nm
- **Data post-processing:** No post-processing of the obtained scans was performed prior to submission.
- **Sample preparation, if any:** The samples were UV/ozone (185 nm and 254 nm LED, 15 mW/cm^2, Ossila Ltd., UK) cleaned for 10 min before each measurement.

TECHNIQUE 2 (if applicable): [NOTE: Please copy/paste this section and repeat for each technique used]

(*Text only, please. We cannot accept citations in this document.)*

- **Common name of technique:** AFM (Atomic Force Microscopy) in tapping mode
- **Type of technique: atomic force microscope**
- **Instrument manufacturer:** Asylum Research, Oxford Instruments, Santa Barbara, USA
- **Instrument model:** Asylum Research MFP 3D
- **Tip size (if known):** 7 nm radius
- **Expected maximum lateral resolution:** 6 pm noise level
- **Data post-processing:** No post-processing of the obtained scans was performed prior to submission.
- **Sample preparation, if any:** The samples were UV/ozone (185 nm and 254 nm LED, 15 mW/cm^2, Ossila Ltd., UK) cleaned for 10 min before each measurement.

AUTHOR AND TECHNIQUE INFORMATION FOR THE SURFACE-TOPOGRAPHY CHALLENGE

Submit to: SurfaceTopographyChallenge@gmail.com ***(***Deadline: 29 Feb 2024***)***

- We created this template to standardize the information we get from each group.
- Information submitted will be published *verbatim* in the Supplementary Information section.
- ***Please fill out all yellow-highlighted fields to the best of your ability.***

AUTHORSHIP INFORMATION (*Text only, please. We cannot accept citations in this document.)*

- **Author information (please ONLY include authors that directly contributed)**
  - **Author 1:** Berberich, Eliot A.
    - **ORCID ID:** 0009-0009-7398-3670
    - **Address, Line 1:** Department of Mechanical and Manufacturing Engineering, Miami University
    - **Address, Line 2:** 650 E High St, Oxford, OH 45056
  - **Author 2:** Sidebottom, Mark A.
    - **ORCID ID:** 0000-0001-8217-6429
    - **Address, Line 1:** Department of Mechanical and Manufacturing Engineering, Miami University
    - **Address, Line 2:** 650 E High St, Oxford, OH 45056

- **Funding information, if any** *(how it should appear in the acknowledgements)*
  - **Funder:** N/A; **Grant number:** N/A

DESCRIPTION OF TECHNIQUES USED FOR DATA COLLECTION

(For reproducibility purposes, the publishing journal requires that we specify all details of each technique used. If you have any questions or concerns, contact SurfaceTopographyChallenge@gmail.com)

- **Number of techniques used:** 1

TECHNIQUE 1: [NOTE: Please copy/paste this section and repeat for each technique used]

(*Text only, please. We cannot accept citations in this document.)*

- **Common name of technique:** Scanning White Light Interferometry
- **Type of technique:** white light interferometer
- **Instrument manufacturer:** Bruker, Billerica, MA, USA
- **Instrument model:** Bruker Contour GT-I
- **Tip size (if known):** N/A
- **Expected maximum lateral resolution:** 0.38 µm (based on manufacturer's specifications)
- **Data post-processing:** For all samples, a curvature and tilt filter was used to remove the observed tilt and curvature within the sample. The average surface height was set to zero as well.
- **Sample preparation, if any:** We did not perform any surface cleaning or treatment on our samples, just removed them from the plastic containers they were shipped in and went straight to measurement.

AUTHOR AND TECHNIQUE INFORMATION FOR THE SURFACE-TOPOGRAPHY CHALLENGE

Submit to: SurfaceTopographyChallenge@gmail.com ***(***Deadline: 29 Feb 2024***)***

- We created this template to standardize the information we get from each group.
- Information submitted will be published *verbatim* in the Supplementary Information section.
- ***Please fill out all yellow-highlighted fields to the best of your ability.***

AUTHORSHIP INFORMATION (*Text only, please. We cannot accept citations in this document.)*

- **Author information (please ONLY include authors that directly contributed)**
  - **Author 1:** Skaltsas, Dimitrios
    - **ORCID ID:** 0000-0001-5892-3607
    - **Address, Line 1:** School of Naval Architecture and Marine Engineering, National Technical University of Athens
    - **Address, Line 2:** 9 Heroon Polytechniou Street, 5710 Zografos, Greece
  - **Author 2:** Papadopoulos, Christos I.
    - **ORCID ID:** 0000-0002-9818-3495
    - **Address, Line 1:** School of Naval Architecture and Marine Engineering, National Technical University of Athens
    - **Address, Line 2:** 9 Heroon Polytechniou Street, 5710 Zografos, Greece

DESCRIPTION OF TECHNIQUES USED FOR DATA COLLECTION

(For reproducibility purposes, the publishing journal requires that we specify all details of each technique used. If you have any questions or concerns, contact SurfaceTopographyChallenge@gmail.com)

- **Number of techniques used: 1**

TECHNIQUE 1:

- **Common name of technique:** Optical profilometry
- **Type of technique:** digital 3D optical microscope
- **Instrument manufacturer:** Bruker, Billerica, MA, USA
- **Instrument model:** GT-KO Optical Profilometer
- **Tip size (if known):** N/A
- **Expected maximum lateral resolution:** 0.1 nm
- **Data post-processing:** Remove curvature and tilt correction

AUTHOR AND TECHNIQUE INFORMATION FOR THE SURFACE-TOPOGRAPHY CHALLENGE

Submit to: SurfaceTopographyChallenge@gmail.com ***(***Deadline: 29 Feb 2024***)***

- We created this template to standardize the information we get from each group.
- Information submitted will be published *verbatim* in the Supplementary Information section.
- ***Please fill out all yellow-highlighted fields to the best of your ability.***

AUTHORSHIP INFORMATION (*Text only, please. We cannot accept citations in this document.)*

- **Author information (please ONLY include authors that directly contributed)**
  - **Author 1:** Kumar, Shubhendu
    - **ORCID ID:** 0000-0003-2658-2988
    - **Address, Line 1:** School of Polymer Science and Polymer Engineering, University of Akron
    - **Address, Line 2**: 170 University Ave, Akron, OH 44325 USA
  - **Author 2:** Karanjkar , Prachi H.
    - **ORCID ID:** 0009-0000-5354-7454
    - **Address, Line 1:** School of Polymer Science and Polymer Engineering, University of Akron
    - **Address, Line 2:** 170 University Ave, Akron, OH 44325 USA
  - **Author 3:** Dhinojwala, Ali
    - **ORCID ID:** 0009-0002-6466-9419
    - **Address, Line 1:** School of Polymer Science and Polymer Engineering, University of Akron
    - **Address, Line 2:** 170 University Ave, Akron, OH 44325 USA

- **Funding information, if any** *(how it should appear in the acknowledgements)*
  - **Funder:** National Science Foundation; **Grant number:** DMR-*2208464)*
    - *(NOTE: For more than one funder, please copy/paste additional Funding lines)*

DESCRIPTION OF TECHNIQUES USED FOR DATA COLLECTION

(For reproducibility purposes, the publishing journal requires that we specify all details of each technique used. If you have any questions or concerns, contact SurfaceTopographyChallenge@gmail.com)

- **Number of techniques used:** 3

TECHNIQUE 1: [NOTE: Please copy/paste this section and repeat for each technique used]

(*Text only, please. We cannot accept citations in this document.)*

- **Common name of technique:** Stylus
- **Type of technique:** Stylus profilometer
- **Instrument manufacturer:** Bruker, Billerica, MA, USA
- **Instrument model:** Bruker Dektak XT profilometer
- **Tip size (if known):** 2.00 µm radius (as per manufacturer)
- **Expected maximum lateral resolution:** around 2.00 µm
- **Data post-processing:** No Post Processing before submission on contact.engineering
- **Sample preparation, if any:** Rinsed with acetone and ethanol, blown dried with stream of dry N_2 gas before 1st measurement for each sample

---

TECHNIQUE 2 (if applicable): [NOTE: Please copy/paste this section and repeat for each technique used]

(*Text only, please. We cannot accept citations in this document.)*

- **Common name of technique:** AFM
- **Type of technique:**atomic force microscope, tapping mode
- **Instrument manufacturer:** Bruker, Billerica, MA, USA
- **Instrument model:** Dimension Icon
- **Tip size (if known):** less than 15 nm (as per manufacturer)
- **Expected maximum lateral resolution:** 15 nm
- **Data post-processing:** No Post Processing before submission on contact.engineering
- **Sample preparation, if any:** Rinsed with acetone and ethanol, blown dried with stream of dry N_2 gas before 1st measurement for each sample

AUTHOR AND TECHNIQUE INFORMATION FOR THE SURFACE-TOPOGRAPHY CHALLENGE

Submit to: SurfaceTopographyChallenge@gmail.com ***(***Deadline: 29 Feb 2024***)***

TECHNIQUE 2 (if applicable): [NOTE: Please copy/paste this section and repeat for each technique used]

(*Text only, please. We cannot accept citations in this document.)*

- **Common name of technique:** optical 3D profilometer
- **Type of technique:** white light interferometry
- **Instrument manufacturer:** Zygo Corp., Middlefield, CT, USA
- **Instrument model:** Zygo NewView 7300
- **Tip size (if known):**
- **Expected maximum lateral resolution:** 1.128 µm
- **Data post-processing:** Interpolation was used to fill in the missing point using MetroPro 8.3.5 software by of Zygo.
- **Sample preparation, if any:** Rinsed with acetone and ethanol, blown dried with stream of dry N_2 gas before 1st measurement for each sample.

AUTHOR AND TECHNIQUE INFORMATION FOR THE SURFACE-TOPOGRAPHY CHALLENGE

AUTHORSHIP INFORMATION

- **Author information**
  - **Author 1:** Fatemi, Arshia
    - **ORCID ID:** 0000-0003-1923-1784
    - **Address, Line 1:** Robert Bosch GmbH
    - **Address, Line 2:** Robert-Bosch-Campus 1, 71272 Renningen, Germany
  - **Author 2:** Vdovak, Jürgen
    - **ORCID ID:** 0009-0006-4396-7088
    - **Address, Line 1:** Robert Bosch GmbH
    - **Address, Line 2:** Robert-Bosch-Campus 1, 71272 Renningen, Germany
  - **Author 3:** Spies, Charlotte
    - **ORCID ID:** 0000-0003-1653-7286
    - **Address, Line 1:** Robert Bosch GmbH
    - **Address, Line 2:** Robert-Bosch-Campus 1, 71272 Renningen, Germany
    - **Address 2, Line 1:** Department of Microsystems Engineering, University of Freiburg
    - **Address 2, Line 2:** Georges-Köhler-Allee 103, 79110 Freiburg, Germany

DESCRIPTION OF TECHNIQUES USED FOR DATA COLLECTION

- **Number of techniques used:** 3

TECHNIQUE 1:

- **Common name of technique:** Stylus instrument
- **Type of technique:** stylus profilometer/tactile microscope
- **Instrument manufacturer:** Jenoptik Industrial Metrology Germany GmbH, Villingen-Schwenningen, Germany
- **Instrument model:** Waveline W900RC Nanoscan
- **Tip size (if known):** respectively 2µm, 5µm
- **Expected maximum lateral resolution:** respectively 0.05µm, 0.1µm, 0.5µm
- **Data post-processing:** Tilt correction of a 1st order polynominal
- **Sample preparation, if any:** none

TECHNIQUE 2

- **Common name of technique:** Confocal Microscopy
- **Type of technique:** Confocal laser scanning microscope
- **Instrument manufacturer:** NanoFocus AG, Oberhausen, Germany
- **Instrument model:** µsurf custom L
- **Tip size (if known):** N/A
- **Expected maximum lateral resolution:** respectively 0.16µm, 0.325µm
- **Data post-processing:** Tilt correction of a 1st order polynominal (for confocal measurements on Surfaces C62, C71 and S21), no correction (for confocal measurements on Surface S22)
- **Sample preparation, if any:** none in general, for one measurement of Surface C62 cleaning with acetone/isopropanol 1:1 and a lint-free cloth

---

TECHNIQUE 3:

- **Common name of technique:** AFM in tapping mode
- **Type of technique:** atomic force microscope
- **Instrument manufacturer:** DIGITAL INSTRUMENTS, Santa Barbara, CA
- **Instrument model:** Dimension 3100
- **Tip size (if known):** N/A, Tip OTESPA with a force constant of 26 N/m
- **Expected maximum lateral resolution:** 256x256 pixels
- **Data post-processing:** Flattening of 1st order and Plane Fitting of 2nd
- **Sample preparation, if any:** none

AUTHOR AND TECHNIQUE INFORMATION FOR THE SURFACE-TOPOGRAPHY CHALLENGE

Submit to: SurfaceTopographyChallenge@gmail.com ***(***Deadline: 29 Feb 2024***)***

- We created this template to standardize the information we get from each group.
- Information submitted will be published *verbatim* in the Supplementary Information section.
- ***Please fill out all yellow-highlighted fields to the best of your ability.***

AUTHORSHIP INFORMATION (*Text only, please. We cannot accept citations in this document.)*

- **Author information (please ONLY include authors that directly contributed)**
  - **Author 1:** Ugar, Susanta
    - **ORCID ID:** 0009-0002-1167-1726
    - **Address, Line 1:** Department of Mechanical Engineering, Indian Institute of Science, Bengaluru
    - **Address, Line 2:** S78, B Block, IISc Bengaluru, C V Raman Rd, Karnataka,560012, India
  - **Author 2:** Bobji, M. S
    - **ORCID ID:** 0000-0001-8703-928X
    - **Address, Line 1:** Department of Mechanical Engineering, Indian Institute of Science, Bengaluru
    - **Address, Line 2:** C V Raman Ave, Bengaluru, Karnataka,560012, India

DESCRIPTION OF TECHNIQUES USED FOR DATA COLLECTION

(For reproducibility purposes, the publishing journal requires that we specify all details of each technique used. If you have any questions or concerns, contact SurfaceTopographyChallenge@gmail.com)

- **Number of techniques used:** 1

TECHNIQUE 1: [NOTE: Please copy/paste this section and repeat for each technique used]

(*Text only, please. We cannot accept citations in this document.)*

- **Common name of technique:** AFM
- **Type of technique:** Atomic force microscopy, Tapping and Contact mode
- **Instrument manufacturer:** Veeco, Brukar, New York, USA
- **Instrument model:** veeco diinnova
- **Tip size (if known):** 20 nm radius
- **Expected maximum lateral resolution:** 20 nm
- **Data post-processing:** NA
- **Sample preparation, if any:** NA

AUTHOR AND TECHNIQUE INFORMATION FOR THE SURFACE-TOPOGRAPHY CHALLENGE

Submit to: SurfaceTopographyChallenge@gmail.com ***(***Deadline: 29 Feb 2024***)***

- We created this template to standardize the information we get from each group.
- Information submitted will be published *verbatim* in the Supplementary Information section.
- ***Please fill out all yellow-highlighted fields to the best of your ability.***

AUTHORSHIP INFORMATION (*Text only, please. We cannot accept citations in this document.)*

- **Author information (please ONLY include authors that directly contributed)**
  - **Author 1:** Leriche, Cyrian
    - **ORCID ID:** 0000-0001-5012-304X
    - **Address, Line 1:** Advanced Research Center for NanoLithography, University of Amsterdam
    - **Address, Line 2:** Science Park 106, 1098 XG, Amsterdam, The Netherlands
  - **Author 2:** Weber, Bart
    - **ORCID ID:** 0000-0003-4756-4666
    - **Address, Line 1:** Advanced Research Center for NanoLithography, University of Amsterdam
    - **Address, Line 2:** Science Park 106, 1098 XG, Amsterdam, The Netherlands

- **Funding information, if any** *(how it should appear in the acknowledgements)*
  - **Funder:** This work was conducted at the Advanced Research Center for Nanolithography, a public-private partnership between the University of Amsterdam (UvA), Vrije Universiteit Amsterdam (VU), Rijksuniversiteit Groningen (RUG), the Netherlands Organization for Scientific Research (NWO), and the semiconductor equipment manufacturer ASML.

DESCRIPTION OF TECHNIQUES USED FOR DATA COLLECTION

(For reproducibility purposes, the publishing journal requires that we specify all details of each technique used. If you have any questions or concerns, contact SurfaceTopographyChallenge@gmail.com)

- **Number of techniques used:** <Atomic Force Microscopy, used in tapping mode>

TECHNIQUE 1: [NOTE: Please copy/paste this section and repeat for each technique used]

(*Text only, please. We cannot accept citations in this document.)*

- **Common name of technique:** AFM
- **Type of technique:** Atomic Force Microscope, tapping mode
- **Instrument manufacturer:** Bruker, Billerica, MA, USA
- **Instrument model:** Dimension ICON
- **Tip size (if known):** 20 nm radius
- **Expected maximum lateral resolution:** 20 nm
- **Data post-processing:** Tilt correction, automatic rows alignment to correct (rare) measurement scars
- **Sample preparation, if any:** The sample was subsequently sonicated in each of the following solvent for 30 min per solvent Acetone, Isopropanol, Ethanol and Distillated water

AUTHOR AND TECHNIQUE INFORMATION FOR THE SURFACE-TOPOGRAPHY CHALLENGE

Submit to: SurfaceTopographyChallenge@gmail.com ***(***Deadline: 29 Feb 2024***)***

- We created this template to standardize the information we get from each group.
- Information submitted will be published *verbatim* in the Supplementary Information section.
- ***Please fill out all yellow-highlighted fields to the best of your ability.***

AUTHORSHIP INFORMATION (*Text only, please. We cannot accept citations in this document.)*

- **Author information (please ONLY include authors that directly contributed)**
  - **Author 1:** Lee, Wonhyeok
    - **ORCID ID:** 0000-0002-6825-1506
    - **Address, Line 1:** Department of Mechanical Engineering, University of Wisconsin-Madison
    - **Address, Line 2:** 1513 University Ave, Madison, WI, 53706, USA
  - **Author 2:** Eriten, Melih
    - **ORCID ID:** 0000-0003-3961-6235
    - **Address, Line 1:** Department of Mechanical Engineering, University of Wisconsin-Madison
    - **Address, Line 2:** 1513 University Ave, Madison, WI, 53706, USA
  - *(NOTE: For three or more authors, please copy/paste additional Author lines)*

- **Funding information, if any** *(how it should appear in the acknowledgements)*
  - **Funder:** US National Science Foundation; **Grant number:** CMMI-2224380
    - *(NOTE: For more than one funder, please copy/paste additional Funding lines)*

DESCRIPTION OF TECHNIQUES USED FOR DATA COLLECTION

(For reproducibility purposes, the publishing journal requires that we specify all details of each technique used. If you have any questions or concerns, contact SurfaceTopographyChallenge@gmail.com)

- **Number of techniques used:** 1

TECHNIQUE 1: [NOTE: Please copy/paste this section and repeat for each technique used]

(*Text only, please. We cannot accept citations in this document.)*

- **Common name of technique:** Optical Profilometer
- **Type of technique:** white light interferometer chromatic aberration
  - [atomic force microscope; white light interferometer chromatic aberration; digital 3D optical microscope; confocal laser scanning microscope; holographic measurement; angle-resolved spectroscopy; scanning electron microscope; reconstruction from scanning electron microscope; stylus profilometer/tactile microscope; transmission electron microscope; triboindenter; other (specify)]
- **Instrument manufacturer:** Zygo Corporation, Middlefield, CT
- **Instrument model:** Zygo NewView 9000
- **Tip size (if known):** N/A
- **Expected maximum lateral resolution:** 0.43 µm
- **Data post-processing:** Piecewise cubic interpolation for void data
- **Sample preparation, if any:** Sample cleaned before conducting tests by a cotton swab soaked with alcohol

AUTHOR AND TECHNIQUE INFORMATION FOR THE SURFACE-TOPOGRAPHY CHALLENGE

Submit to: SurfaceTopographyChallenge@gmail.com ***(***Deadline: 29 Feb 2024***)***

- We created this template to standardize the information we get from each group.
- Information submitted will be published *verbatim* in the Supplementary Information section.
- ***Please fill out all yellow-highlighted fields to the best of your ability.***

AUTHORSHIP INFORMATION (*Text only, please. We cannot accept citations in this document.)*

- **Author information (please ONLY include authors that directly contributed)**
    - **Author 1:** Wolski, Marcin
        - **ORCID ID:** 0000-0003-0501-069X
        - **Address, Line 1:** Tribology Laboratory, School of Civil and Mechanical Engineering, Curtin University
        - **Address, Line 2:** GPO Box U1987, Perth, WA 6845, Australia
    - **Author 2:** Woloszynski, Tomasz
        - **ORCID ID:** 0000-0001-7822-9348
        - **Address, Line 1:** Tribology Laboratory, School of Civil and Mechanical Engineering, Curtin University
        - **Address, Line 2:** GPO Box U1987, Perth, WA 6845, Australia
    - **Author 3:** Stachowiak, Gwidon W.
        - **ORCID ID:** N/A
        - **Address, Line 1:** Tribology Laboratory, School of Civil and Mechanical Engineering, Curtin University
        - **Address, Line 2:** GPO Box U1987, Perth, WA 6845, Australia
    - **Author 4:** Podsiadlo, Pawel
        - **ORCID ID:** 0000-0002-0386-7908
        - **Address, Line 1:** Tribology Laboratory, School of Civil and Mechanical Engineering, Curtin University
        - **Address, Line 2:** GPO Box U1987, Perth, WA 6845, Australia

- **Funding information, if any** *(how it should appear in the acknowledgements)*
    - **Funder:** N/A; **Grant number:** N/A
        - *(NOTE: For more than one funder, please copy/paste additional Funding lines)*

DESCRIPTION OF TECHNIQUES USED FOR DATA COLLECTION

(For reproducibility purposes, the publishing journal requires that we specify all details of each technique used. If you have any questions or concerns, contact SurfaceTopographyChallenge@gmail.com)

- **Number of techniques used:** 1

TECHNIQUE 1: [NOTE: Please copy/paste this section and repeat for each technique used]

(*Text only, please. We cannot accept citations in this document.)*

- **Common name of technique:** profilometer
- **Type of technique:** chromatic confocal surface profilometer
- **Instrument manufacturer:** Altimet, Marin, France
- **Instrument model:** AltiSurf 530
- **Tip size (if known):** N/A
- **Expected maximum lateral resolution:** 0.1 µm
- **Data post-processing:** Directional blanket covering (DBC) method
- **Sample preparation, if any:** standard sample cleaning

AUTHOR AND TECHNIQUE INFORMATION FOR THE SURFACE-TOPOGRAPHY CHALLENGE

Submit to: SurfaceTopographyChallenge@gmail.com ***(***Deadline: 29 Feb 2024***)***

- We created this template to standardize the information we get from each group.
- Information submitted will be published *verbatim* in the Supplementary Information section.
- ***Please fill out all yellow-highlighted fields to the best of your ability.***

AUTHORSHIP INFORMATION (*Text only, please. We cannot accept citations in this document.*)

- **Author information (please ONLY include authors that directly contributed)**
  - **Author 1:** Yastrebov, Vladislav A.
    - **ORCID ID:** 0000-0002-4052-3557
    - **Address, Line 1:** Centre des matériaux, MINES Paris – PSL, CNRS UMR 7633
    - **Address, Line 2:** BP 87, 91003 Evry, France
  - **Author 2:** Gaslain, Fabrice
    - **ORCID ID:** 0000-0001-5187-1613
    - **Address, Line 1:** Centre des matériaux, MINES Paris – PSL, CNRS UMR 7633
    - **Address, Line 2:** BP 87, 91003 Evry, France

- **Funding information, if any** *(how it should appear in the acknowledgements)*
  - **Funder:** N/A; **Grant number:** N/A

DESCRIPTION OF TECHNIQUES USED FOR DATA COLLECTION

(For reproducibility purposes, the publishing journal requires that we specify all details of each technique used. If you have any questions or concerns, contact SurfaceTopographyChallenge@gmail.com)

- **Number of techniques used: 1**

TECHNIQUE 1: [NOTE: Please copy/paste this section and repeat for each technique used]

(*Text only, please. We cannot accept citations in this document.*)

- **Common name of technique:** multi-view SEM based topography reconstruction
- **Type of technique:** reconstruction from scanning electron microscope
  - [atomic force microscope; white light interferometer chromatic aberration; digital 3D optical microscope; confocal laser scanning microscope; holographic measurement; angle-resolved spectroscopy; scanning electron microscope; reconstruction from scanning electron microscope; stylus profilometer/tactile microscope; transmission electron microscope; triboindenter; other (specify)]
- **Instrument manufacturer:** FEI (now Thermo Fisher Scientific Inc.), Hillsboro, Oregon, United States
- **Instrument model:** FEI NOVA NANOSEM 450
- **Tip size (if known):** N/A
- **Expected maximum lateral resolution:** 1 nm at 15 kV
- **Data post-processing:** The data was obtained using a scanning electron microscope (SEM) equipped with a multi-view backscattered electron (BSE) detector. The 3D surfaces *z(x,y)* were reconstructed using a technique [1] from 3 BSE detector signals using (1) Singular Value Decomposition of images to recover principal images, (2) Radon transform to determine the angles to obtain gradients along OX and OY directions, and (3) Frankot and Chellappa FFT-based method [2] was used to to reconstruct the surface from its gradients. Tilts and curvatures were removed. To obtain a z-scaling factor, a Vickers indentation was carried out on a Fe surface (with similar BSE reflective properties) and measured on with the same detector. SEM2Surface code (Python open-source code implemented for this challenge) was used github.com/vyastreb/sem2surface for the reconstruction.
  [1] Neggers, J., Héripré, E., Bonnet, M., Boivin, D., Tanguy, A., Hallais, S., Gaslain, F., Rouesne, E. and Roux, S. (2021). Principal image decomposition for multi-detector backscatter electron topography reconstruction. Ultramicroscopy, 227:113200.
  [2] Frankot, R. T., & Chellappa, R. (1988). A method for enforcing integrability in shape from shading algorithms. IEEE Transactions on pattern analysis and machine intelligence, 10(4):439-451.

- **Sample preparation, if any:** every sample was plasma treated for 2 minutes (Ag O2 (1:1) plasma at 20 Watt and 65 Pa) to remove eventual volatile organic surface contamination

AUTHOR AND TECHNIQUE INFORMATION FOR THE SURFACE-TOPOGRAPHY CHALLENGE

Submit to: SurfaceTopographyChallenge@gmail.com ***(***Deadline: 29 Feb 2024***)***

- We created this template to standardize the information we get from each group.
- Information submitted will be published *verbatim* in the Supplementary Information section.
- ***Please fill out all yellow-highlighted fields to the best of your ability.***

AUTHORSHIP INFORMATION (*Text only, please. We cannot accept citations in this document.)*

- **Author information (please ONLY include authors that directly contributed)**

  - **Author 1:** Esawi, Amal M.K.
    - **ORCID ID:** 0000-0001-9515-400X
    - **Address, Line 1:** Department of Mechanical Engineering, The American University in Cairo
    - **Address, Line 2:** AUC Avenue. P.O. Box 74, New Cairo, 11835, Egypt
  - **Author 2:** Haroun, Amir N.L.
    - **ORCID ID:** 0009-0007-7910-2171
    - **Address, Line 1:** Department of Mechanical Engineering, The American University in Cairo
    - **Address, Line 2:** AUC Avenue. P.O. Box 74, New Cairo, 11835, Egypt

- **Funding information, if any** *(how it should appear in the acknowledgements)*

    - **Funder:** The American University in Cairo; **Grant number:** SSE-MENG-A.E-FY24-MG-2024-May-26-18-08-40

DESCRIPTION OF TECHNIQUES USED FOR DATA COLLECTION

(For reproducibility purposes, the publishing journal requires that we specify all details of each technique used. If you have any questions or concerns, contact SurfaceTopographyChallenge@gmail.com)

- **Number of techniques used:** 1

TECHNIQUE 1: [NOTE: Please copy/paste this section and repeat for each technique used]

(*Text only, please. We cannot accept citations in this document.)*

- **Common name of technique:** AFM
- **Type of technique:** Atomic Force Microscope, contact mode
- **Instrument manufacturer:** Veeco/Bruker, Billerica, MA, USA
- **Instrument model:** Dimension 3100
- **Tip size (if known):** Nominal: 10 nm
- **Expected maximum lateral resolution:** Not known
- **Data post-processing:** Not Applicable
- **Sample preparation, if any:**

  Simple cleaning using Nitrogen gun.

AUTHOR AND TECHNIQUE INFORMATION FOR THE SURFACE-TOPOGRAPHY CHALLENGE

Submit to: SurfaceTopographyChallenge@gmail.com ***(***Deadline: 29 Feb 2024***)***

- We created this template to standardize the information we get from each group.
- Information submitted will be published *verbatim* in the Supplementary Information section.
- ***Please fill out all yellow-highlighted fields to the best of your ability.***

AUTHORSHIP INFORMATION (*Text only, please. We cannot accept citations in this document.)*

- **Author information (please ONLY include authors that directly contributed)**
  - **Author 1:** Marian, Max
    - **ORCID ID:** 0000-0003-2045-6649
    - **Address, Line 1:** Pontificia Universidad Católica de Chile, School of Engineering, Department of Mechanical and Metallurgical Engineering
    - **Address, Line 2:** Vicuña Mackenna 4860, Macul, Santiago 6904411, Chile
    - **&**
    - **Address, Line 1:** Institute of Machine Design and Tribology (IMKT), Leibniz University Hannover
    - **Address, Line 2:** An der Universität 1, 30823 Garbsen, Germany
  - **Author 2:** Walczak, Magdelena
    - **ORCID ID:** 0000-0003-2070-9458
    - **Address, Line 1:** Pontificia Universidad Católica de Chile, School of Engineering, Department of Mechanical and Metallurgical Engineering
    - **Address, Line 2:** Vicuña Mackenna 4860, Macul, Santiago 6904411, Chile

- **Funding information, if any** *(how it should appear in the acknowledgements)*
  - **Funder:** ANID Chile; **Grant number:** DFG220004

DESCRIPTION OF TECHNIQUES USED FOR DATA COLLECTION

(For reproducibility purposes, the publishing journal requires that we specify all details of each technique used. If you have any questions or concerns, contact SurfaceTopographyChallenge@gmail.com)

- **Number of techniques used:** 2

TECHNIQUE 1: [NOTE: Please copy/paste this section and repeat for each technique used]

- **Common name of technique:** White Light Interferometry
- **Type of technique:** White light interferometer
- **Instrument manufacturer:** Rtec
- **Instrument model:** MFT-5000
- **Tip size (if known):** N/A
- **Expected maximum lateral resolution:** N/A (10x, 20x, 50x magnification)
- **Data post-processing:** Raw data was submitted. For calculation of roughness values, tilt and curvature were removed by Mountain®9.
- **Sample preparation, if any:** N/A

TECHNIQUE 2 (if applicable): [NOTE: Please copy/paste this section and repeat for each technique used]

- **Common name of technique:** White Light Interferometry
- **Type of technique:** White light interferometer
- **Instrument manufacturer:** Filmmetrics
- **Instrument model:** Profilm3D
- **Tip size (if known):** N/A
- **Expected maximum lateral resolution:** N/A (10x magnification)
- **Data post-processing:** Raw data was submitted. For calculation of roughness values, tilt and curvature were removed by Mountain®9.
- **Sample preparation, if any:** N/A

AUTHOR AND TECHNIQUE INFORMATION FOR THE SURFACE-TOPOGRAPHY CHALLENGE

Submit to: SurfaceTopographyChallenge@gmail.com ***(***Deadline: 29 Feb 2024***)***

- We created this template to standardize the information we get from each group.
- Information submitted will be published *verbatim* in the Supplementary Information section.
- ***Please fill out all yellow-highlighted fields to the best of your ability.***

AUTHORSHIP INFORMATION (*Text only, please. We cannot accept citations in this document.)*

- **Author information (please ONLY include authors that directly contributed)**
  - **Author 1:** Lyashenko Iakov A.
    - **ORCID ID:** 0000-0001-7511-3163
    - **1-st Affiliation:**
    - **Address, Line 1:** Department of System Dynamics and Friction Physics, Institute of Mechanics, Technische Universität Berlin
    - **Address, Line 2:** Straße des 17. Juni 135, Berlin, 10623, Germany
    - **2-nd Affiliation:**
    - **Address, Line 1:** Department of Theoretical and Applied Mechanics
    - **Address, Line 2:** University blv. 15, Samarkand, 140104, Uzbekistan
  - **Author 2:** Popov Valentin L.
    - **ORCID ID:** 0000-0003-0506-3804
    - **1-st Affiliation:**
    - **Address, Line 1:** Department of System Dynamics and Friction Physics, Institute of Mechanics, Technische Universität Berlin
    - **Address, Line 2:** Straße des 17. Juni 135, Berlin, 10623, Germany
    - **2-nd Affiliation:**
    - **Address, Line 1:** Center of Advanced Studies in Mechanics, Tribology, Bio- and Nanotechnologies, Samarkand State University
    - **Address, Line 2:** University blv. 15, Samarkand, 140104, Uzbekistan

- **Funding information, if any** *(how it should appear in the acknowledgements)*
  - **Funder:** Deutsche Forschungsgemeinschaft; **Grant number:** PO 810/55-3

DESCRIPTION OF TECHNIQUES USED FOR DATA COLLECTION

(For reproducibility purposes, the publishing journal requires that we specify all details of each technique used. If you have any questions or concerns, contact SurfaceTopographyChallenge@gmail.com)

- **Number of techniques used: 1**

TECHNIQUE 1: [NOTE: Please copy/paste this section and repeat for each technique used]

(*Text only, please. We cannot accept citations in this document.)*

- **Common name of technique:** Laser Microscope
- **Type of technique:** confocal laser scanning microscope
  - [atomic force microscope; white light interferometer chromatic aberration; digital 3D optical microscope; confocal laser scanning microscope; holographic measurement; angle-resolved spectroscopy; scanning electron microscope; reconstruction from scanning electron microscope; stylus profilometer/tactile microscope; transmission electron microscope; triboindenter; other (specify)]
- **Instrument manufacturer:** Keyence, Neu-Isenburg, Germany
- **Instrument model:** Keyence VK-X150
- **Tip size (if known):** N/A
- **Expected maximum lateral resolution:** We conducted 2 measurements using a 100X magnification lens: (1) an area of approximately 0.389 x 0.283 mm^2 and (2) an area of approximately 1.256 x 0.903 mm^2. For the smaller area (1), the horizontal distance between neighboring points (discretization) in both x- and y-directions in the resulting data files is about 69.149 nm, whereas for the larger area (2), it is about 276.596 nm.
- **Data post-processing:** N/A
- **Sample preparation, if any:** N/A

AUTHOR AND TECHNIQUE INFORMATION FOR THE SURFACE-TOPOGRAPHY CHALLENGE

Submit to: SurfaceTopographyChallenge@gmail.com ***(***Deadline: 29 Feb 2024***)***

- We created this template to standardize the information we get from each group.
- Information submitted will be published *verbatim* in the Supplementary Information section.
- ***Please fill out all yellow-highlighted fields to the best of your ability.***

AUTHORSHIP INFORMATION (*Text only, please. We cannot accept citations in this document.)*

- **Author information (please ONLY include authors that directly contributed)**
  - **Author 1:** Grejtak, Tomas
    - **ORCID ID:** 0000-0002-0995-1166
    - **Address, Line 1:** Materials Science and Technology Division, Oak Ridge National Laboratory
    - **Address, Line 2:** 1 Bethel Valley Road, Oak Ridge, TN, 37830, USA

- **Funding information, if any** *(how it should appear in the acknowledgements)*
  The research was sponsored by the Feedstock-Conversion Interface Consortium (FCIC) of the Bioenergy Technologies Office, Office of Energy Efficiency and Renewable Energy (EERE), US Department of Energy (DOE).

DESCRIPTION OF TECHNIQUES USED FOR DATA COLLECTION

(For reproducibility purposes, the publishing journal requires that we specify all details of each technique used. If you have any questions or concerns, contact SurfaceTopographyChallenge@gmail.com)

- **Number of techniques used:** 1

TECHNIQUE 1: [NOTE: Please copy/paste this section and repeat for each technique used]

(*Text only, please. We cannot accept citations in this document.)*

- **Common name of technique:** White light interferometer
  - **Type of technique:** white light interferometer chromatic aberration
- **Instrument manufacturer:** Veeco Instruments Inc., Plainview, NY
- **Instrument model:** Wyko NT9100
- **Tip size (if known):**
- **Expected maximum lateral resolution:** 642 nm
- **Data post-processing:** Tilt correction (plane fit with Vision 4.10 software.
- **Sample preparation, if any:** Samples were rinsed in isopropanol.

AUTHOR AND TECHNIQUE INFORMATION FOR THE SURFACE-TOPOGRAPHY CHALLENGE

Submit to: SurfaceTopographyChallenge@gmail.com *(***Deadline: 29 Feb 2024***)*

- We created this template to standardize the information we get from each group.
- Information submitted will be published *verbatim* in the Supplementary Information section.
- ***Please fill out all yellow-highlighted fields to the best of your ability.***

AUTHORSHIP INFORMATION (*Text only, please. We cannot accept citations in this document.)*

- **Author information (please ONLY include authors that directly contributed)**

  - **Author 1:** Schaefer, Florian
    - **ORCID ID:** 0000-0002-7513-4738
    - **Address, Line 1:** Dep. Materials Science and Methods, Saarland University
    - **Address, Line 2:** Campus D2 3, 66123 Saarbruecken, Germany

  - *(NOTE: For three or more authors, please copy/paste additional Author lines)*

- **Funding information, if any** *(how it should appear in the acknowledgements)*
  - **Funder:** no funding was received; **Grant number:** -
    - *(NOTE: For more than one funder, please copy/paste additional Funding lines)*

DESCRIPTION OF TECHNIQUES USED FOR DATA COLLECTION

(For reproducibility purposes, the publishing journal requires that we specify all details of each technique used. If you have any questions or concerns, contact SurfaceTopographyChallenge@gmail.com)

- **Number of techniques used:** 1

TECHNIQUE 1: [NOTE: Please copy/paste this section and repeat for each technique used]

(*Text only, please. We cannot accept citations in this document.)*

- **Common name of technique:** AFM, Atomic Force Microscopy
- **Type of technique:** atomic force microscopy
  - [atomic force microscope; white light interferometer chromatic aberration; digital 3D optical microscope; confocal laser scanning microscope; holographic measurement; angle-resolved spectroscopy; scanning electron microscope; reconstruction from scanning electron microscope; stylus profilometer/tactile microscope; transmission electron microscope; triboindenter; other (specify)]
- **Instrument manufacturer:** Bruker, US
- **Instrument model:** Dimension Icon, Peak Force Tapping
- **Tip size (if known):** <10 nm
- **Expected maximum lateral resolution:** 10 nm
- **Data post-processing:** 2nd order background substraction
- **Sample preparation, if any:** cleaning with isopropanol in ultrasonic bath

---

TECHNIQUE 2 (if applicable): [NOTE: Please copy/paste this section and repeat for each technique used]

(*Text only, please. We cannot accept citations in this document.)*

- **Common name of technique:** <Generic name; not brand names>
- **Type of technique:** <Choose one of the following categories>
  - [atomic force microscope; white light interferometer chromatic aberration; digital 3D optical microscope; confocal laser scanning microscope; holographic measurement; angle-resolved spectroscopy; scanning electron microscope; reconstruction from scanning electron microscope; stylus profilometer/tactile microscope; transmission electron microscope; triboindenter; other (specify)]
- **Instrument manufacturer:** <Name, city, state>
- **Instrument model:** <Brand name and model number>
- **Tip size (if known):** <Insert "N/A" for non-contact instruments>
- **Expected maximum lateral resolution:** <Based on manufacturer specs or technique info>
- **Data post-processing:** <Briefly describe any processing you did (such as tilt-correction or artifact removal) prior to submission of the data – either on the collection instrument or in other software.>
- **Sample preparation, if any:** <Briefly describe any sample preparation (cleaning, hot-mounting, etc.)>

AUTHOR AND TECHNIQUE INFORMATION FOR THE SURFACE-TOPOGRAPHY CHALLENGE

Submit to: SurfaceTopographyChallenge@gmail.com ***(***Deadline: 29 Feb 2024***)***

- We created this template to standardize the information we get from each group.
- Information submitted will be published *verbatim* in the Supplementary Information section.
- ***Please fill out all yellow-highlighted fields to the best of your ability.***

AUTHORSHIP INFORMATION (*Text only, please. We cannot accept citations in this document.)*

- **Author information (please ONLY include authors that directly contributed)**
  - **Author 1:** Brodmann, Boris
    - **ORCID ID:** 0000-0002-1529-5708
    - **Address, Line 1:** OptoSurf GmbH
    - **Address, Line 2:** Nobelstr. 9-13 76275 Ettlingen, Germany

DESCRIPTION OF TECHNIQUES USED FOR DATA COLLECTION

(For reproducibility purposes, the publishing journal requires that we specify all details of each technique used. If you have any questions or concerns, contact SurfaceTopographyChallenge@gmail.com)

- **Number of techniques used:** 1

TECHNIQUE 1: [NOTE: Please copy/paste this section and repeat for each technique used]

(*Text only, please. We cannot accept citations in this document.)*

- **Common name of technique:** light scattering
- **Type of technique:** ARS scatterometer according to VDA2009
- **Instrument manufacturer:** OptoSurf, Germany
- **Instrument model:** OS500-09
- **Tip size (if known):** Spot size 0.9mm
- **Expected maximum lateral resolution:** 2.4µm according to RR Theory
- **Data post-processing:** raw data
- **Sample preparation, if any:** none

AUTHOR AND TECHNIQUE INFORMATION FOR THE SURFACE-TOPOGRAPHY CHALLENGE

Submit to: SurfaceTopographyChallenge@gmail.com ***(***Deadline: 29 Feb 2024***)***

- We created this template to standardize the information we get from each group.
- Information submitted will be published *verbatim* in the Supplementary Information section.
- ***Please fill out all yellow-highlighted fields to the best of your ability.***

AUTHORSHIP INFORMATION (*Text only, please. We cannot accept citations in this document.)*

- **Author information (please ONLY include authors that directly contributed)**

  - **Author 1:** Chowdhury, Md Istiaque
    - **ORCID ID:** 0000-0002-1920-2978
    - **Address, Line 1:** Department of Materials Science and Engineering, Lehigh University
    - **Address, Line 2:** 5 East Packer Avenue, Bethlehem, PA, 18015, USA
  - **Author 2:** Strandwitz, Nicholas C
    - **ORCID ID:** 0000-0001-6159-9430
    - **Address, Line 1:** Department of Materials Science and Engineering, Lehigh University
    - **Address, Line 2:** 5 East Packer Avenue, Bethlehem, PA, 18015, USA

- **Funding information, if any** *(how it should appear in the acknowledgements)*

  - **Funder:** U.S. National Science Foundation; **Grant number:** 1826251

DESCRIPTION OF TECHNIQUES USED FOR DATA COLLECTION

(For reproducibility purposes, the publishing journal requires that we specify all details of each technique used. If you have any questions or concerns, contact SurfaceTopographyChallenge@gmail.com)

- **Number of techniques used:** 1.

TECHNIQUE 1: [NOTE: Please copy/paste this section and repeat for each technique used]

(*Text only, please. We cannot accept citations in this document.)*

- **Common name of technique:** XRR
- **Type of technique:** X-ray reflectivity
- **Instrument manufacturer:** Malvern Panalytical B.V., Almelo, Netherlands
- **Instrument model:** PANalytical, Empyrean
- **Tip size (if known):** N/A
- **Expected maximum lateral resolution:** Averaged over the entire sample surface.
- **Data post-processing:** The data is fitted against a cross-sectional layered model using a proprietary fitting software (X'Pert Reflectivity V1.3a) to determine the roughness of the films.
- **Sample preparation, if any:** None

AUTHOR AND TECHNIQUE INFORMATION FOR THE SURFACE-TOPOGRAPHY CHALLENGE
Submit to: SurfaceTopographyChallenge@gmail.com ***(***Deadline: 29 Feb 2024***)***

- We created this template to standardize the information we get from each group.
- Information submitted will be published *verbatim* in the Supplementary Information section.
- ***Please fill out all yellow-highlighted fields to the best of your ability.***

AUTHORSHIP INFORMATION (*Text only, please. We cannot accept citations in this document.*)

- **Author information (please ONLY include authors that directly contributed)**
  - **Author 1:** Kumar, Charchit
    - **ORCID ID:** 0000-0002-6912-3506
    - **Address, Line 1:** Materials and Manufacturing Research Group, James Watt School of Engineering, University of Glasgow
    - **Address, Line 2:** Glasgow, G12 8QQ, United Kingdom
  - **Author 2:** Mulvihill, Daniel M.
    - **ORCID ID:** 0000-0003-1693-0088
    - **Address, Line 1:** Materials and Manufacturing Research Group, James Watt School of Engineering, University of Glasgow
    - **Address, Line 2:** Glasgow, G12 8QQ, United Kingdom

- **Funding information, if any** *(how it should appear in the acknowledgements)*
  - **Funder:** UK Engineering and Physical Sciences Research Council (EPSRC); **Grant number:** EP/V003380/1

DESCRIPTION OF TECHNIQUES USED FOR DATA COLLECTION
(For reproducibility purposes, the publishing journal requires that we specify all details of each technique used. If you have any questions or concerns, contact SurfaceTopographyChallenge@gmail.com)

- **Number of techniques used:** 2

TECHNIQUE 1: [NOTE: Please copy/paste this section and repeat for each technique used]
(*Text only, please. We cannot accept citations in this document.*)

- **Common name of technique:** 3D optical profiler
- **Type of technique:** White light interferometry
- **Instrument manufacturer:** Bruker USA
- **Instrument model:** Bruker Contour GT-X
- **Tip size (if known):** N/A
- **Expected maximum lateral resolution:** 0.38 μm
- **Data post-processing:** Tilt correction using an open-source platform https://contact.engineering/.
- **Sample preparation, if any:** Samples were cleaned using pressurized air

---

TECHNIQUE 2 (if applicable): [NOTE: Please copy/paste this section and repeat for each technique used]
(*Text only, please. We cannot accept citations in this document.*)

- **Common name of technique:** 3D surface profilometer
- **Type of technique:** 3D optical microscope
- **Instrument manufacturer:** Alicona Imaging GmbH, Austria
- **Instrument model:** Alicona Infinite Focus G4
- **Tip size (if known):** N/A
- **Expected maximum lateral resolution:** 0.6 μm
- **Data post-processing:** Tilt-correction was carried out on the recorded data using an open-source platform https://contact.engineering/.
- **Sample preparation, if any:** Samples were cleaned using pressurized air

AUTHOR AND TECHNIQUE INFORMATION FOR THE SURFACE-TOPOGRAPHY CHALLENGE

Submit to: SurfaceTopographyChallenge@gmail.com ***(***Deadline: 29 Feb 2024***)***

AUTHORSHIP INFORMATION

- **Author information**
  - **Author 1:** Delplanque, Emilie
    - **ORCID ID:**
    - **Address, Line 1:** LTDS, CNRS UMR5513, Ecole centrale de Lyon
    - **Address, Line 2:** 36 avenue guy de collongue, Ecully, 69130, France
  - **Author 2:** Mazuyer, Denis
    - **ORCID ID:** 0000-0001-9828-6884
    - **Address, Line 1:** LTDS, CNRS UMR5513, Ecole centrale de Lyon
    - **Address, Line 2:** 36 avenue guy de collongue, Ecully, 69130, France
  - **Author 3:** Cayer-Barrioz, Juliette*
    - **ORCID ID:** 0000-0002- 3601-2957
    - **Address, Line 1:** LTDS, CNRS UMR5513, Ecole centrale de Lyon
    - **Address, Line 2:** 36 avenue guy de collongue, Ecully, 69130, France

- **Funding information, if any**
  - **Funder:** French agency for the ecological transition ADEME; **Grant number:** IMOTEP
  - **Funder: SURFAB** surface engineering platform, Ecole centrale Lyon

DESCRIPTION OF TECHNIQUES USED FOR DATA COLLECTION

(For reproducibility purposes, the publishing journal requires that we specify all details of each technique used. If you have any questions or concerns, contact SurfaceTopographyChallenge@gmail.com)

- **Number of techniques used: 3**

TECHNIQUE 1:

- **Common name of technique:** Interferometer
- **Type of technique:** 3D optical microscope
- **Instrument manufacturer:** Bruker Nano, Tucson, AZ, USA
- **Instrument model:** Bruker Contour GT-K
- **Tip size (if known):** N/A
- **Expected maximum lateral resolution:** 150 nm
- **Data post-processing:** Remove curvature and tilt correction using DigitalSurf Mountains Map software
- **Sample preparation, if any:** uncleaned and cleaned with isopropanol and dried under Argon flow

---

TECHNIQUE 2:

- **Common name of technique:** AFM
- **Type of technique:** atomic force microscope
- **Instrument manufacturer:** Nanosurf AG, Liestal, Switzerland
- **Instrument model:** Core AFM
- **Tip size (if known):**
- **Expected maximum lateral resolution:** 5 nm if flatness
- **Data post-processing:** Tilt correction and 1st degree polynomial background removal using DigitalSurf Mountains Map software
- **Sample preparation, if any:** uncleaned and cleaned with isopropanol and dried under Argon flow

---

TECHNIQUE 3:

- **Common name of technique:** Interferometer
- **Type of technique:** 3D optical microscope
- **Instrument manufacturer:** Bruker Nano, Tucson, AZ, USA
- **Instrument model:** Bruker NPFlex-1000
- **Tip size (if known):** N/A
- **Expected maximum lateral resolution:** 150 nm
- **Data post-processing:** Remove curvature and tilt correction using DigitalSurf Mountains Map software
- **Sample preparation, if any:** uncleaned and cleaned with isopropanol and dried under Argon flow

AUTHOR AND TECHNIQUE INFORMATION FOR THE SURFACE-TOPOGRAPHY CHALLENGE

Submit to: SurfaceTopographyChallenge@gmail.com ***(***Deadline: 29 Feb 2024***)***

- We created this template to standardize the information we get from each group.
- Information submitted will be published *verbatim* in the Supplementary Information section.
- ***Please fill out all yellow-highlighted fields to the best of your ability.***

AUTHORSHIP INFORMATION (*Text only, please. We cannot accept citations in this document.)*

- **Author information (please ONLY include authors that directly contributed)**
  - **Author 1:** <Last name, Given name and middle initial(s)>
    - **ORCID ID:** <Insert ORCID ID here, please double-check for accuracy>
    - **Address, Line 1:** <Dept, University or Company>
    - **Address, Line 2:** <Street Address, City, State, ZIP code, Country>
  - **Author 2:** <Last name, Given name and middle initial(s)>
    - **ORCID ID:** <Insert ORCID ID here, please double-check for accuracy>
    - **Address, Line 1:** <Dept, University or Company>
    - **Address, Line 2:** <Street Address, City, State, ZIP code, Country>
  - *(NOTE: For three or more authors, please copy/paste additional Author lines)*
- **Funding information, if any** *(how it should appear in the acknowledgements)*
  - **Funder:** <Insert funding agency>; **Grant number:** <Insert grant number >
    - *(NOTE: For more than one funder, please copy/paste additional Funding lines)*

DESCRIPTION OF TECHNIQUES USED FOR DATA COLLECTION

(For reproducibility purposes, the publishing journal requires that we specify all details of each technique used. If you have any questions or concerns, contact SurfaceTopographyChallenge@gmail.com)

- **Number of techniques used:** <Insert the number of separate techniques applied>

TECHNIQUE 1: [NOTE: Please copy/paste this section and repeat for each technique used]

(*Text only, please. We cannot accept citations in this document.)*

- **Common name of technique:** <Generic name; not brand names>
- **Type of technique:** <Choose one of the following categories>
  - [atomic force microscope; white light interferometer chromatic aberration; digital 3D optical microscope; confocal laser scanning microscope; holographic measurement; angle-resolved spectroscopy; scanning electron microscope; reconstruction from scanning electron microscope; stylus profilometer/tactile microscope; transmission electron microscope; triboindenter; other (specify)]
- **Instrument manufacturer:** <Name, city, state>
- **Instrument model:** <Brand name and model number>
- **Tip size (if known):** <Insert "N/A" for non-contact instruments>
- **Expected maximum lateral resolution:** <Based on manufacturer specs or technique info>
- **Data post-processing:** <Briefly describe any processing you did (such as tilt-correction or artifact removal) prior to submission of the data – either on the collection instrument or in other software>
- **Sample preparation, if any:** <Briefly describe any sample preparation (cleaning, hot-mounting, etc.)>

TECHNIQUE 2 (if applicable): [NOTE: Please copy/paste this section and repeat for each technique used]

(*Text only, please. We cannot accept citations in this document.)*

- **Common name of technique:** <Generic name; not brand names>
- **Type of technique:** <Choose one of the following categories>
  - [atomic force microscope; white light interferometer chromatic aberration; digital 3D optical microscope; confocal laser scanning microscope; holographic measurement; angle-resolved spectroscopy; scanning electron microscope; reconstruction from scanning electron microscope; stylus profilometer/tactile microscope; transmission electron microscope; triboindenter; other (specify)]
- **Instrument manufacturer:** <Name, city, state>
- **Instrument model:** <Brand name and model number>
- **Tip size (if known):** <Insert "N/A" for non-contact instruments>
- **Expected maximum lateral resolution:** <Based on manufacturer specs or technique info>
- **Data post-processing:** <Briefly describe any processing you did (such as tilt-correction or artifact removal) prior to submission of the data – either on the collection instrument or in other software.>
- **Sample preparation, if any:** <Briefly describe any sample preparation (cleaning, hot-mounting, etc.)>
